# Hsa_circRNA_0040462: a sensor of cells' response to CAP treatment with double-edged roles on breast cancer malignancy

**DOI:** 10.7150/ijms.66940

**Published:** 2022-03-21

**Authors:** Yue Tian, Zhifa Zhang, Zijing Zhang, Xiaofeng Dai

**Affiliations:** 1 Wuxi School of Medicine, Jiangnan University, Wuxi, 214122, China.; 2 CAPsoul Medical Biotechnology Company, Ltd., Beijing, 100000, China.

**Keywords:** triple negative breast cancer, circular RNA, cold atmospheric plasma, epigenetics

## Abstract

Cold atmospheric plasma (CAP) represents a novel onco-therapeutic approach that has demonstrated its efficacy in many types of tumors. The efficacy of CAP is dose-dependent that determines the panel of tumors feasible for receiving CAP treatment under a certain parameter configuration. Identifying markers for easy and fast prognosis of tumors' sensitivity in response to CAP exposure is of critical value towards optimized therapeutic outcome, the lack of which has largely limited the translation of CAP into clinics. Circular RNAs represent a novel type of biomarkers for disease diagnosis that is featured by easy detection and stability. Through whole transcriptome sequencing, followed by *in vitro* validations, computational predictions and preliminary functional studies, we identified hsa_circRNA_0040462 as a sensor of breast cancer cells' response to CAP treatment. Yet we warrant the use of hsa_circRNA_0040462 as an onco-therapeutic target given its double-edged roles on breast cancer progression, i.e., suppressive on the growth and promotive on the migrative ability of triple negative breast cancer cells. Our study for the first time focused on markers prognostic of CAP's efficacy and tumors' sensitivity to CAP treatment under a certain parameter configuration, and reported hsa_circRNA_0040462 as a sensor of cells' response to CAP treatment. Also, the uncovered dual roles of hsa_circRNA_0040462 further advanced our knowledge on the complex yet critical regulatory functionalities of circular RNAs in cancer progression.

## Introduction

Circular RNAs (circRNAs), a novel type of RNA prevalently present in the eukaryotic transcriptome, form a covalently closed continuous loop 1. CircRNAs are primarily produced from exonic or intronic sequences, where reverse complementary sequences or RNA-binding proteins (RBPs) are needed for their biogenesis 2. CircRNA molecules are enriched with microRNA (miRNA) binding sites and thus could function as a type of competitive endogenous RNA to regulate the expression of target genes 3. Different from the traditional linear RNA, most circRNAs are highly conserved, stably expressed, and cannot be easily degraded by RNA exonuclease given their unique ring-like structures 4, rendering them easily to be detected in the blood and ideal candidate biomarkers for the diagnosis or prognosis of complex diseases including cancers 5.

Cold atmospheric plasma (CAP), being the fourth state of matter and a cocktail of reactive oxygen and nitrogen species (e.g., OH·, H_2_O_2_, O_3_, O, O^2-^, NO, OONO^-^, ONOOH), has demonstrated its efficacy in killing many types of cancer cells such as melanoma 6, triple negative breast cancer (TNBC) 7, bladder cancer 8, prostate cancer 9, liver cancer 10 and pancreatic cancer 11. The most commonly accepted theory attributes the selectivity of CAP against cancer cells to its ability in imposing cells with redox stress that selectively triggers cancer cell death. That is, malignant cells have, in general, a higher redox level than their healthy peers, which renders them more sensitive to redox perturbation and CAP treatment 12. Others also attribute the higher sensitivity of cancer cells in response to CAP treatment to the differential cell membrane features of malignant cells as compared with their healthy state, which include, e.g., over-represented aquaporins that mediates RONS entry 13 and low cholesterol fraction that promotes RONS permission 14. Besides the fast-moving field of plasma oncology in consecutively reporting the varied types of cancers sensitive to CAP exposure and uncovering the mechanism that fosters its anti-cancer selectivity, the clinical translation of CAP as a novel onco-therapeutic approach is also on the way. The success in curing a 75-year-old late-stage pancreatic cancer patients using CAP in 2016 15 has led to the U.S. Food and Drug Administration (FDA) approval on conducting clinical trials using CAP as an onco-therapy in mid-2019. The first trail aimed to save the life of a 33-year-old patient with a relapsed incurable peritoneal sarcoma patient 16.

Through the whole-transcriptome sequencing of different types of breast cancer cells with and without CAP exposure, followed by network construction and a series of experimental validations, we identified a circular RNA, namely hsa_circRNA_0040462, highly sensitive to CAP treatment in TNBC cells. Preliminary dry and wet lab functionality studies unveiled its doubled-edged roles in tumor progression, i.e., halting cancer cell proliferation while promoting their migratory ability. Thus, we propose hsa_circRNA_0040462 as a novel marker diagnostic of cancer cells' sensitivity to CAP treatment and a sensor of CAP's efficacy; in addition, we warrant the use of hsa_circRNA_0040462 in cancer therapeutics provided with its uncovered dual functionalities in cancer progression.

## Materials and Methods

### Home-made CAP source

**Figure 1:**
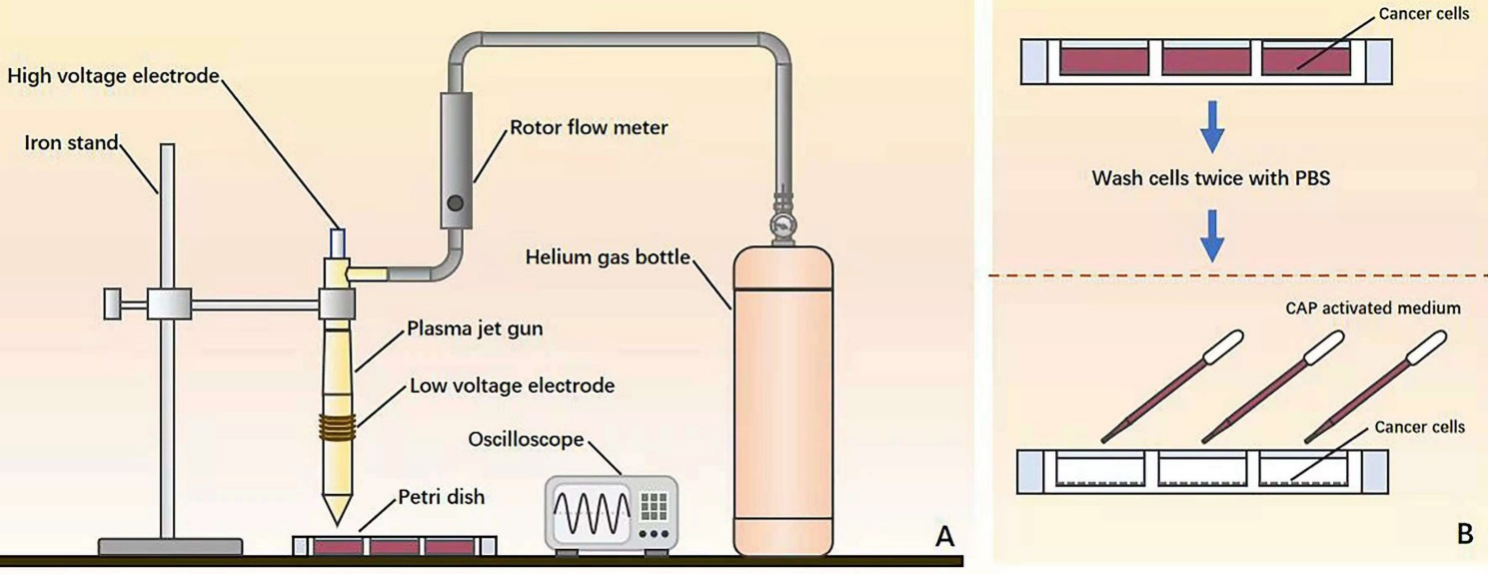
Illustration on the (**A**) home-made CAP source and (**B**) experimental setup.

The device consists of a power controller, helium (He) gas cylinder, rotor flow meter, oscilloscope and plasma jet (**Figure 1A**). The applied voltage ranged from 11V to 12V. The flow rate of He gas was 1 L/min and the distance between the plasma injection and the medium surface was from 0.6 cm to 1cm. The exposure duration was 4 min/well in a 24-well plate setup where each well contained 2 mL CAP-activated medium (PAM). During the experiment, cancer cells were pre-washed using PBS twice before being cultured with PAM (**Figure 1B**).

### Cell culture

The TNBC cell line SUM159PT and luminal A cell line MCF7 were used in this study, both of which were purchased from BeNa Culter Collection (BNCC). The SUM159PT cell line was cultured using Ham's F-12 medium (Damas, C8017) supplemented with 5% fetal bovine serum (FBS) (Gemini-Bio, 900-108), 0.00325% (1IU) insulin, 1% (1M) HEPES (Solarbio, H1090), 138 μL (10 Mm/mL) hydrocortisone and 1% Penicillin Streptomycin (Gibco, 15140-122). The MCF7 cell line was cultured in DMEM/HIGH GLUCOSE medium (Damas, SH30243.01) containing 10% FBS and 1% Penicillin Streptomycin.

### Cell proliferation assay

Cells were grown in 96-well plates at a density of approximately 8×10^6^/well for 48 h. The 96-well plate was refreshed with new media containing 1% thiazolyl blue tetrazolium bromide solution (MTT) (Solarbio, M8180) and cultured in an incubator at 37 °C for 4 h. The media was replaced with 0.1 mL dimethyl sulfoxide (DMSO) (Greagent, G75927B) and subjected to slight shaking for 10 min. OD value was measured by ELIASA at 590 nm.

### Cell scratch assay

Over 5×10^8^/well cells were grown in 6-well plates overnight, where two parallel lines were drawn at the bottom of the plates. Two vertical lines were scratched on cell surface using tips. The plates were washed by phosphate buffered saline (PBS) for 3 times and then cells were cultured using serum-free media. Cell migration was monitored at different time points with images taken using inverted phase contrast microscope (ZEISS). Image J software was used to measure the width of the scratch to calculate the migration rate.

### ROS assay

Cells were cultured in 12-well plates where each well had round coverslip on the bottom. Cells were dyed by ROS Fluorescent Probe-Dihydroethidium (DHE) (Beyotime, S0063) at 37 °C in the darkness for 1 h. The nucleus was dyed using DAPI. The slides were covered using round coverslips. Fluorescence intensity was recorded using fluorescence microscope (ZEISS, Imager.Z2). Software Image J was used to measure the mean fluorescence intensity to reflect the concentration of ROS.

### qPCR

Total RNA was collected from the cells using Ultrapure RNA Kit (Cwbio, CW0581S) and reverse transcribed to cDNA by PrimeScript™ RT reagent Kit with gDNA Eraser following the manufacturer's protocol (Takara, RR047A). 4 μL cDNA samples, 5 μL 2×UltraSYBR MixTure (Cwbio, CW0957M), 0.2 μL forward and 0.2 μL backward primers, 0.6 μL ddH_2_O were mixed and centrifuged. The mixture was placed in Gradient thermal cycler (Eppendorf, Mastercycler nexus GX2), pre-denatured at 95 °C for 10 min, and run with '95 °C for 10 sec, 60 °C for 1 min, 72 °C for 20 sec' for 40 cycles.

### Western blot

Cells were cultured in 6-well plates, washed twice by pre-chilled PBS, supplemented with lysis buffer (RIPA: protease inhibitor: phosphatase inhibitors = 100:1:1), placed on ice for 20 min, and centrifuged at 12,000 g for 20 min to collect the supernatants. BCA Proteib analysis kit (Beyotime, P0010) was used to estimate and quantify protein concentration. Proteins were separated by 10% SDS-PAGE gel at 125 V for 75 min and transferred to a PVDF membrane at 200 mA for 120 min. The membrane was blocked with 5% skim milk in TBST for 1 h followed by incubation with the primary antibody at 4 °C overnight. The membrane was washed 3 times, each for 5 min, and incubated with the secondary antibody for 1 h at the room temperature. The membrane was washed again and added with high sensitivity ECL chemiluminescence detection kit (Vazyme, E412-01) before being visualized in the darkness using Automatic chemiluminescence image analysis system (Tanon, 4600). The signals of bands were measured and quantified by Image J software.

Antibodies used in this study are listed in Table 1.

### Whole transcriptome sequencing and data analysis

SUM159PT and MCF7 cells at the time points of 0 h, 1 h and 8 h post-CAP exposure were collected, each with three replicates. Total RNA of these 18 samples were extracted and sequenced using HiSeqX10 (Illumina) by Vazyme Company (Nanjing, China).

Raw reads were pre-processed by Vazyme using their in-house pipeline. Differentially expressed transcripts were identified as those with |log_2_(fold change)| ≥1 and corrected p value <0.05.

### CircRNA-miRNA-mRNA network and functional enrichment analysis

The mRNAs positively correlated with hsa_circRNA_0040462 were identified from our whole transcriptome dataset, namely 'mRNA_set1', by calculating the Pearson correlation score, with the absolute value of the correlation score being over 0.8 and p < 0.05 being used as the selection threshold. The miRNAs regulated by mRNAs were defined as 'miRNA_set1' and predicted through starBase (http://starbase.sysu.edu.cn/) 17, with parameters being set as 'type = validate'.

The miRNAs regulated by hsa_circRNA_0040462 (defined as 'miRNA_set2') were predicted using Miranda (http://www.miranda.org/) 18, with parameters being set as 'sc >150' and 'en < -7'. The mRNAs regulated by the miRNA_set2 module were named as 'mRNA_set2' and predicted using miRDB (http://www.mirdb.org/) 19, which were considered as mRNAs directly regulated by hsa_circRNA_0040462 without CAP perturbation.

KEGG pathway enrichment analysis and Gene Ontology (GO) enrichment analysis were conducted for the union of mRNA_set1 and mRNA_set2, and for mRNA_set2, respectively, using the R package 'clusterProfiler' to assess the functional significance of genes regulated by hsa_circRNA_0040462 regardless of CAP perturbation and cell response, respectively. Differentially identified KEGG pathway and GO terms were considered to be involved in cells' response to CAP treatment. Fisher's exact test was used to assess the statistical significance. The p-values were adjusted using Benjamini-Hochberg false discovery rate (FDR), with the adjusted p<0.01 being considered as the cutoff threshold.

The intersection between miRNA_set1 and miRNA_set2 was taken and considered as the set of miRNAs regulated by hsa_circRNA_0040462 and regulating experimentally identified mRNAs from mRNA_set1 (denoted as 'miRNA module') in response to CAP exposure. The miRNA module, mRNAs in 'mRNA_set2' and hsa_circRNA_0040462 were subjected to circRNA-miRNA-mRNA network construction using Cytoscape 20.

## Results

### CAP effectively halts the proliferation and migration of breast cancer cells

**Figure 2:**
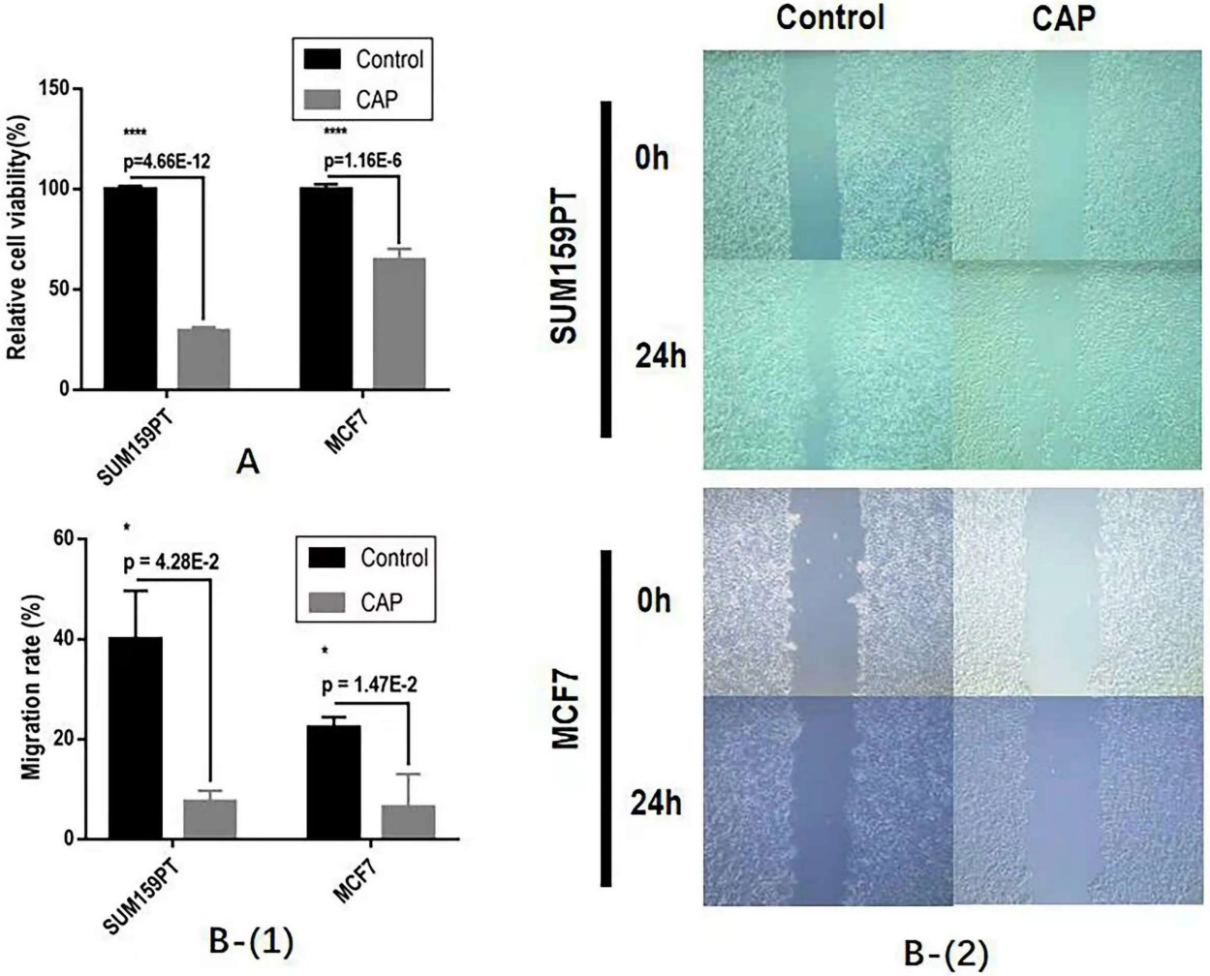
The effect of CAP on breast cancer cell (**A**) growth and (**B**) migrative ability.

CAP could significantly halt the growth and migrative ability of both SUM159PT and MCF7 cells. Specifically, CAP selectively reduced the growth of SUM159PT cells to approximately 1/3 of its untreated peers and suppressed that of MCF7 to around 3/5 of its control, both with p<1E-4 (**Figure 2A**). The migration rates have been reduced to 20% and 40%, respectively, for SUM159PT and MCF7 cells, each with a significance of 4.28E-2 and 1.47E-2 (**Figure 2B**).

### Whole transcriptome sequencing unveils hsa_circRNA_0040462 as a sensor of cells' response to CAP treatment

**Figure 3:**
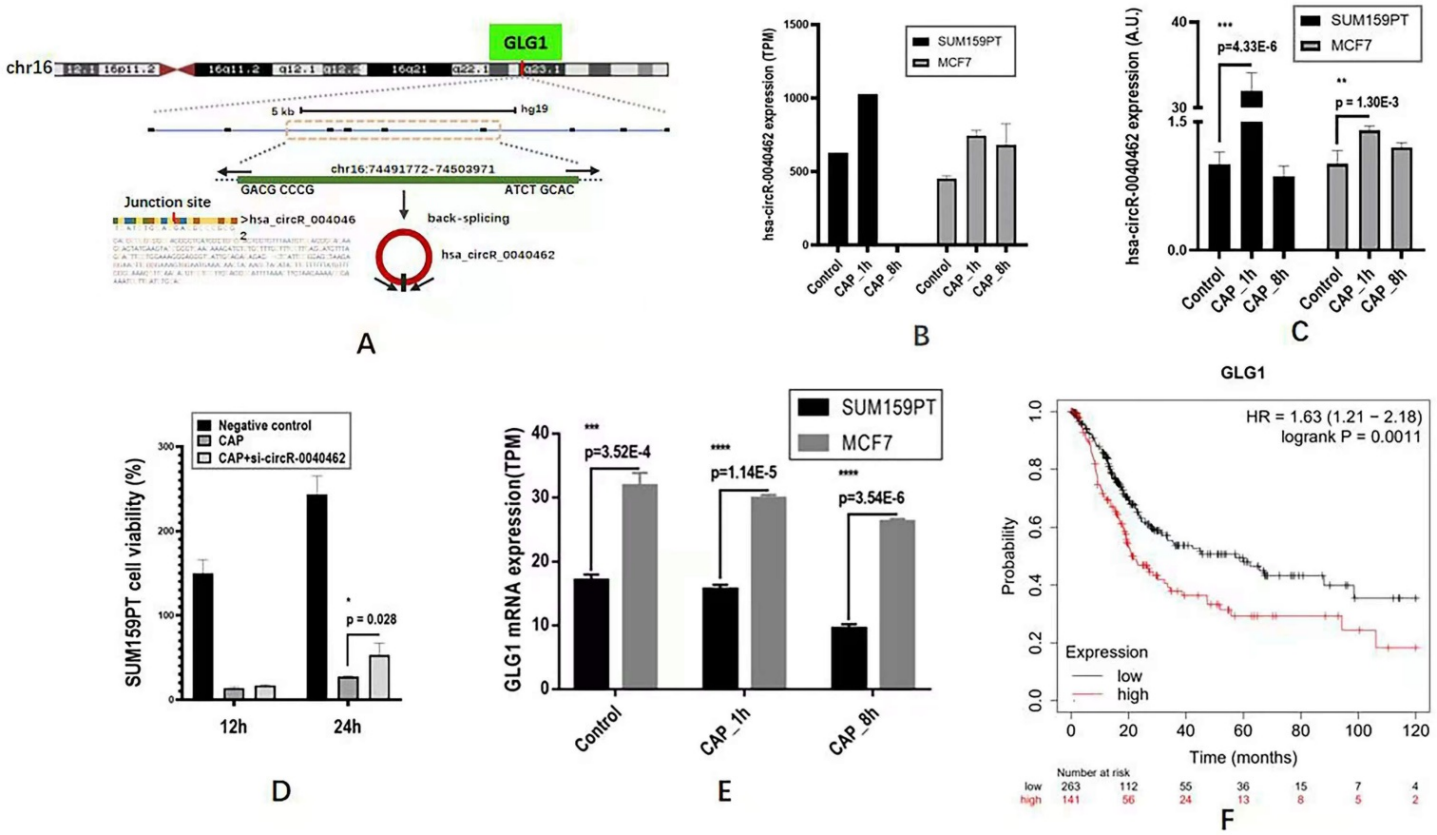
** The expression of hsa_circRNA_0040462 in response to CAP treatment and cancer associated features of its host gene *GLG1*. (A)** Genomic location of hsa_circRNA_0040462. The expression level of hsa_circRNA_0040462 1h post-CAP treatment in SUM159PT and MCF7 cells (**B**) from the transcriptome data, and (**C**) validated *in vitro*. The Y axis represents the folds of hsa_circRNA_0040462 expression on CAP treatment as compared with that of the control. **(D)** SUM159PT cell proliferation in response to CAP treatment without and with knocking down hsa_circRNA_0040462. **(E)** The gene expression level of GLG1 1h and 8h post-CAP treatment in SUM159PT and MCF7 cells. **(F)** Breast cancer patient 10-year relapse-free survival using protein expression data as assessed via Kaplan-Meier Plotter. 'TPM' represents 'transcripts per million', 'A.U.' represents 'arbitrary unit'.

Through differentially expressed gene analysis, we identified one circRNA, hsa_circRNA_0040462, highly expressed in both SUM159PT and MCF7 cells 1 h post-CAP exposure but not in MCF7 cells (**Figure 3A**). In particular, hsa_circRNA_0040462 increased to approximately 1.5 folds in both SUM159PT and MCF7 cells (**Figure 3B**). This has been experimentally validated* in vitro*, where hsa_circRNA_0040462 expression increased 27 folds (p=4.33E-6, **Figure 3C**) in SUM159PT cells and enhanced in MCF7 cells, though slightly, with statistical significance 1 h post-CAP exposure (p=0.0013, **Figure 3C**). In addition, relapsed cell proliferation on CAP exposure was observed with statistical significance (p=0.028) in SUM159PT cells when hsa_circRNA_0040462 was silenced, consolidating the role of hsa_circRNA_0040462 in being a sensor of cells' response to CAP treatment (**Figure 3D**). These results collectively suggested the high sensitivity of hsa_circRNA_0040462 in response to CAP treatment especially in SUM159PT cells.

The level of the host gene (GLG1) of hsa_circRNA_0040462 was higher in MCF7 than in SUM159PT cells, which decreased on CAP exposure and decreased with the exposure duration (**Figure 3E**). Specifically, GLG1 showed approximately half expression in SUM159PT cells as compared with that in MCF7 cells before and after CAP exposure (p=3.52E-4 for the control, p=1.14E-5 for 1 h post-CAP, p=3.54E-6 for 8 h post-CAP, **Figure 3E**). These are suggestive of the tumor suppressive role of GLG1, which has been confirmed by its clinical association. In particular, 10-year relapse-free survival of protein expression data from 126 patients was conducted (Liu_2014) using Kaplan-Meier Plotter (https://kmplot.com/analysis/) 21 where the median was used for data stratification, and the results showed a favorable prognostic value of GLG1 on breast cancer survival (HR=0.35, p=8.6E-4, **Figure 3F**).

### Computational analysis reveals hsa_circRNA_0040462 as a regulator of cell proliferation, migration and anti-cancer stemness

We obtained 22 mRNAs ('mRNA_set1') whose transcription levels were highly and positively correlated with hsa_circRNA_0040462 (**Supplementary Table 1**), and regulated by 276 miRNAs ('miRNA_set1') on CAP exposure (**Supplementary Table 2**). On the other hand, we found that hsa_circRNA_0040462 regulated the expression of 8 miRNAs ('miRNA_set2', **Supplementary Table 2**) and 3394 mRNAs ('mRNA_set2', **Supplementary Table 1**) without CAP perturbation.

The intersection of miRNA_set1 and miRNA_set2 included 4 miRNAs (i.e., hsa-miRNA-27a-3p, hsa-miRNA-27b-3p, hsa-miRNA-221-5p, hsa-miRNA-1-3p), and that of mRNA_set1 and mRNA_set2 contained only one gene (i.e., *clec7a*) that was regulated by hsa-miRNA-32-3p. These 4 miRNAs and the one mRNA were considered to be the downstream players of hsa_circRNA_0040462 regardless of CAP exposure.

**Figure 4:**
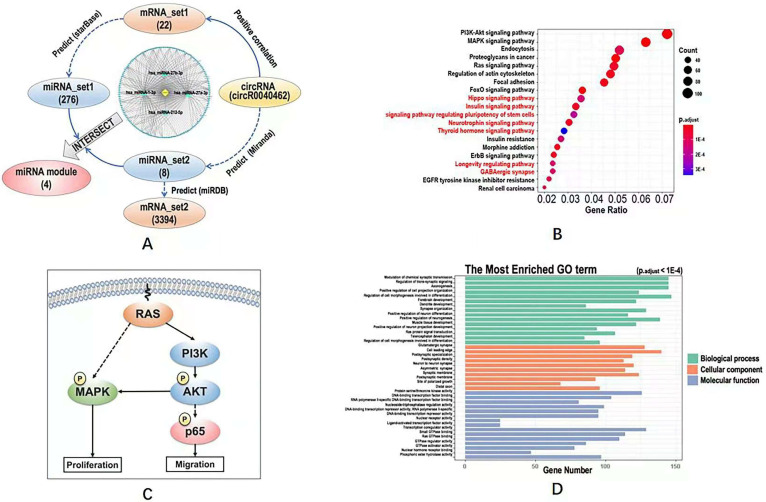
** Computational analysis on the KEGG pathways, GO terms and regulatory network downstream of hsa_circRNA_0040462. (A)** Workflow of the bioinformatics analysis and the constructed circRNA-miRNA-mRNA network. KEGG pathways predicted using (**B**) mRNA_set2 with pathways not identified using mRNA_set1+2 being characterized in red. **(C)** Simplified regulatory network of canonical pathways popped up in KEGG analysis. GO terms predicted using mRNA_set1+2 and mRNA_set2 were identical which were shown in (**D**). The definitions of mRNA_set1, mRNA_set2, miRNA_set1, miRNA_set2 were illustrated in Figure 4A. The mRNA_set1+2 is the union of mRNA_set1 and mRNA_set2.

The circRNA-miRNA-mRNA network constructed using hsa_circRNA_0040462, the 4 miRNAs and mRNA_set1 showed that most edges were connected via hsa-miRNA-1-3p (**Figure 4A**), suggestive of its pivotal role in hsa_circRNA_0040462-triggered signal relay.

Define KEGG pathways and GO terms obtained by taking the union of mRNA_set1 and mRNA_set2 as 'KEGG_set1/2' and 'GO_set1/2', and those obtained from mRNA_set2 as 'KEGG_set2' and 'GO_set2'. KEGG pathways identified using mRNA_set1/2 was a subset of those enriched using mRNA_set2. PI3K/AKT and MAPK signalings were the top pathways enriched in both KEGG_set1/2 and KEGG_set2 (**Figure 4B, Supplementary Figure 1, Supplementary Table 3**), which are all canonical pathways responsible for cell proliferation (**Figure 4C**). Ras signaling was revealed from both KEGG and GO analyses using both '_set1/2' and '_set2' datasets (**Figure 4B, Supplementary Figure 1, Figure 4D, Supplementary Table 3**), which is the upstream player of PIK3/AKT and MAPK pathways (**Figure 4C**). 'Signaling pathway regulating pluripotency of stem cells' was shown available in the KEGG analysis of the '_set2' dataset but vanished in that of the '_set1/2' dataset (**Figure 4B**). GO terms enriched from both '_set1/2' and '_set2' datasets were identical (**Figure 4D, Supplementary Table 3**). We examined the effect of hsa_circRNA_0040462 on the level of total and phosphorylated AKT as the PI3K/AKT signaling was ranked first from the enriched KEGG pathways (**Figure 4B**), and found that phosphorylated PI3K, phosphorylated AKT and total AKT levels were all substantially elevated by silencing hsa_circRNA_0040462 (**Supplementary Figure 2**).

### Hsa_circRNA_0040462 plays double-edged roles in breast cancer progression

**Figure 5:**
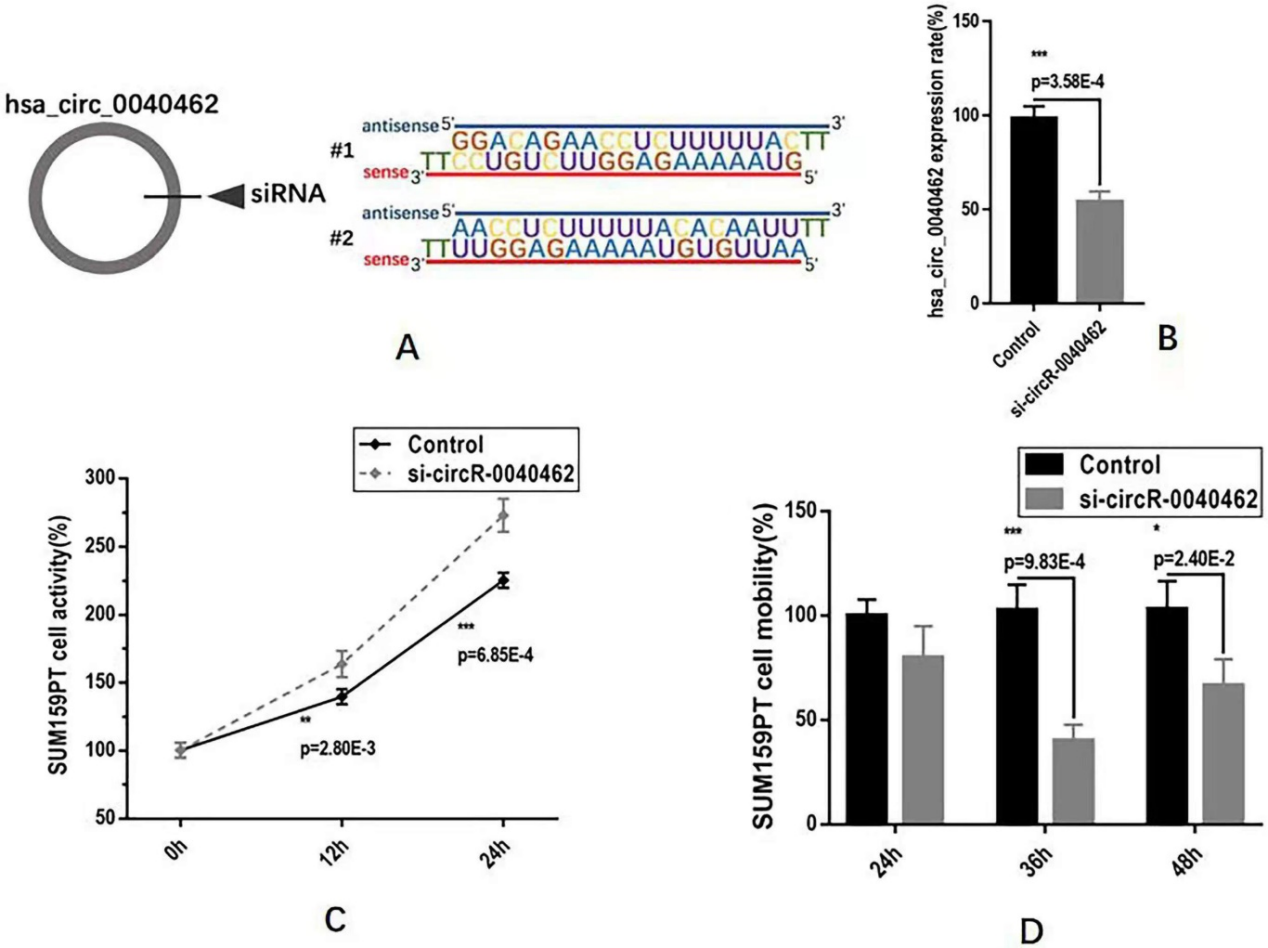
** Assays examining the effect of hsa_circRNA_0040462 on breast cancer cells. (A)** Sequences of the designed siRNAs for has_circRNA_0040462. **(B)** Knocking down effect of the hsa_circRNA_0040462 siRNA. **(C)** SUM159PT cell proliferation after knocking down hsa_circRNA_0040462. **(D)** SUM159PT cell migration after knocking down hsa_circRNA_0040462.

We next examined the functionalities of hsa_circRNA_0040462 in cancer progression *in vitro* using SUM159PT cells. By knocking down hsa_circRNA_0040462 using self-designed siRNAs (**Figure 5A**), we reduced the expression of this circRNA to almost half of its basal level with statistical significance (p=3.58E-4, **Figure 5B**). Cells with reduced hsa_circRNA_0040462 expression exhibited significantly enhanced growth rate in SUM159PT cells (p=0.0028 at 12 h, p=6.85E-4 at 24 h, **Figure 5C**). Interestingly, reduced hsa_circRNA_0040462 level led to suppressed mobility of SUM159PT cells (p=9.83E-4 at 36 h, p=0.024 at 48 h, **Figure 5D**).

**Figure 6:**
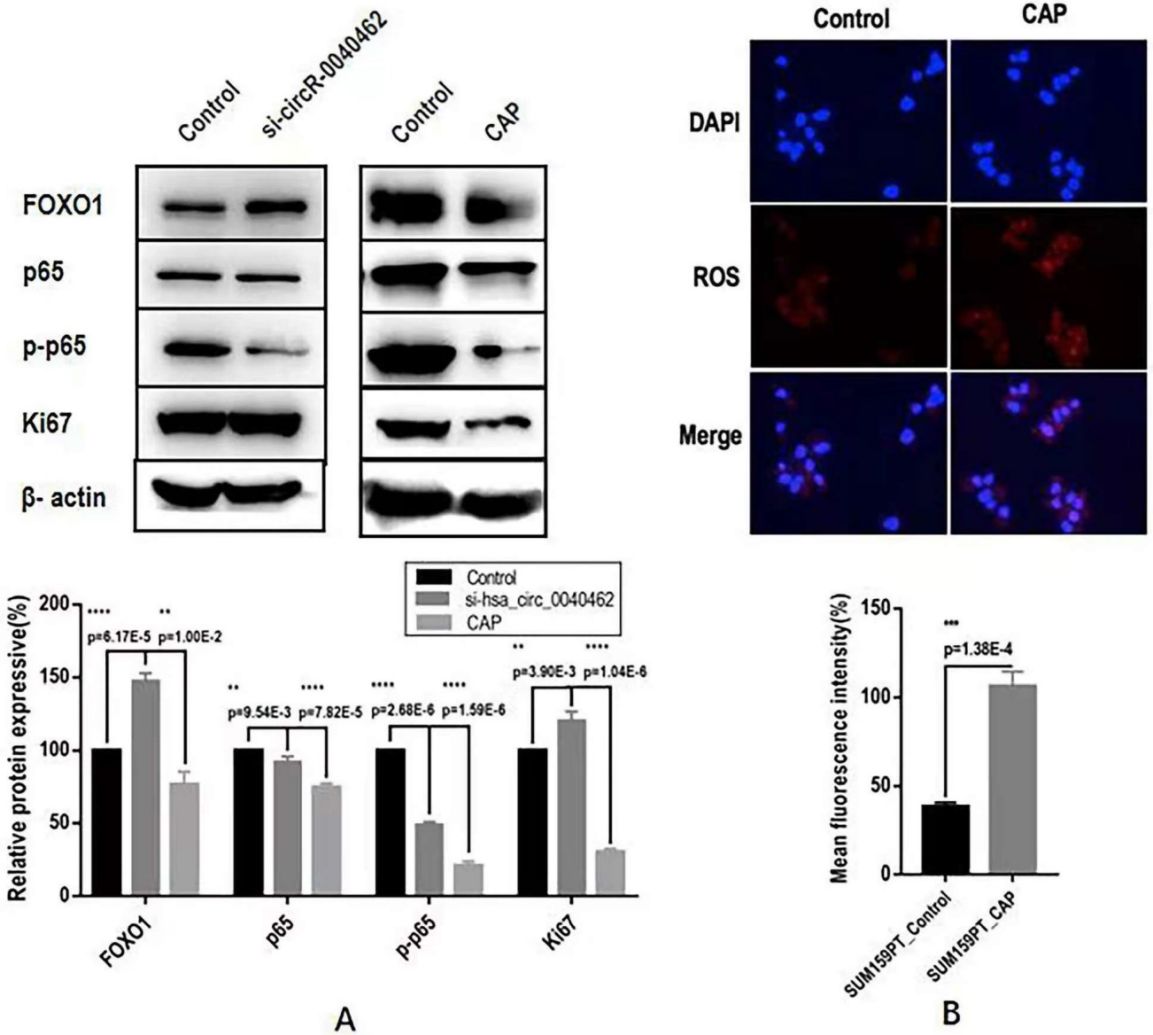
** Assays examining the effect of hsa_circRNA_0040462 on breast cancer cells. (A)** Western blots and quantification of several canonical makers on cancer cell proliferation, migration, and anti-oxidant ability after knocking down hsa_circRNA_0040462 or CAP treatment. **(B)** Immunofluorescence imaging and quantification of ROS levels after CAP exposure.

Through computational analysis, the prominent role of PI3K/AKT, MAPK and RAS signaling in hsa_circRNA_0040462-assisted cell proliferation, and the importance of players contributing to cell migration, cell stemness and anti-oxidant were revealed (**Figure 4D**). By examining the protein expression of KI67 (a cancer proliferation marker 22), p65 (a protein responsible for cancer migration 23) and its phosphorylated state (p-p65) that indicates its activity, FOXO1 (a marker showing cells anti-oxidant ability and being associated with cancer stemness 24), we found that silencing hsa_circRNA_0040462 could, in general, promote tumor progression given the enhanced FOXO1 and KI67 expression (p=6.17E-5 for FOXO1, p=3.9E-3 for KI67, **Figure 6A**). However, suppressed hsa_circRNA_0040462 expression reduced the activity of p65 to approximately half of the control (p=2.68E-6, **Figure 6A**) without substantially affecting its total protein level (**Figure 6A**), suggestive of inhibited migrative ability of cancer cells.

Contrary to knocking down hsa_circRNA_0040462, CAP significantly suppressed FOXO1 (p=1E-2) and KI67 (p=1.04E-6), where KI67 expression reduced to 40% of its original level, suggesting the consistent regulatory direction of CAP and hsa_circRNA_0040462 in cancer cell proliferation. However, CAP also dramatically reduced both the levels of p65 and its phosphorylated levels (p=7.82E-5 for p65, p=1.59E-6 for p-p65, **Figure 5B**). It is important to notice that knocking down hsa_circRNA_0040462 suppressed p-p65 to half of its control, yet CAP reduced it to 20% of its original level (**Figure 5B**), suggesting that the effect of CAP on p-p65 overrides that of hsa_circRNA_0040462 despite its promotive role on hsa_circRNA_0040462 expression.

## Discussion

CAP, being proposed as an emerging onco-therapeutic approach, is dose-dependent 25. Different types of cancer cells differ in their sensitivities to CAP exposure, rendering 'accurate and easy monitor on cells' response to CAP treatment' a critical bottleneck that limits the translation of CAP into clinics. Through transcriptome sequencing followed by *in vitro* validation and preliminary dry and wet lab functional studies, we identified hsa_circRNA_0040462 as a sensor of cells' response to CAP treatment.

Though CAP showed selectivity against breast cancer cell proliferation and migration, and substantially elevated the expression of hsa_circRNA_0040462 in these malignant cells (**Figure 3B, 3C**), hsa_circRNA_0040462 suppressed TNBC cell growth but promoted cells' migrative ability (**Figure 5C, 5D**). Further investigations on primary signaling molecules using western blotting indicated the opposite roles of CAP and hsa_circRNA_0040462 on the activated form of p65 (phosphorylated p65, **Figure 6A**), a molecule primarily responsible for cell migration. The fact that hsa_circRNA_0040462 activated p65 but suppressed cell proliferation and antioxidant ability suggested its double-edged roles in cancer progression given its numerous downstream targets and the complexity of the regulatory network it was involved in. CAP, on the other hand, could systematically adjust the network signaling and thus exhibited a stronger suppressive role on cell migration to override the effect of hsa_circRNA_0040462 on p-p65 (**Figure 6**). Thus, CAP could elevate the redox level of cells and target their anti-oxidant ability towards halted cancer cell progression that cannot be explained by any single pathway or hub gene; and hsa_circRNA_0040462, a marker identified with high sensitivity to CAP treatment, may only be able to function as a sensor of CAP efficacy but not a therapeutic target.

We identified 4 miRNAs (i.e., hsa-miRNA-1-3p, hsa-miRNA-27a-3p, hsa-miRNA-27b-3p, hsa-miRNA-221-5p) and one gene (i.e., *clec7a*) as important downstream players of hsa_circRNA_0040462, with hsa-miRNA-1-3p being the hub of the constructed network. These molecules have all been associated with cancer. For instance, dysregulation of hsa-miRNA-1-3p has recently been associated with breast cancer 26, prostate cancer 27, colorectal cancer 28, and lung cancer 29; altered hsa-miRNA-27a-3p has been shown to be involved in the carcinogenesis of breast cancer 30, gastric cancer 31, osteosarcoma 32, cholangiocellular carcinoma 33, glioblastoma multiforme 34 and spinal cord glioma 35; dysregulated hsa-miRNA-27b-3p was revealed to be associated with gastric cancer 36 and lung cancer 37; and hsa-miRNA-221-5p aberration was proposed for hepatocellular carcinoma prognosis 38. The gene *clec7a* that encodes a glycoprotein with a distinct role in innate immunity regulation was up-regulated after CAP treatment 39. While *clec7a* over-expression conferred a prior drug resistance in leukemic cells 40, 41 and was prognostic of poor prostate cancer relapse-free survival 42, it constituted as one member of the immune-related prognostic panel associated with favorable melanoma survival 43 whose mutation has been associated with immunodeficiency and the pathogenesis of retinoblastoma 44.

We only used two cell lines (SUM159PT and MCF7), each representing a distinct type of breast cancer subtype, in this study to keep consistent with the number of cell lines used in the whole transcriptome sequencing. The findings should be carried over to other breast cancer cell lines for additional validations.

Through a series of computational analyses, we unveiled the importance of PI3K/AKT and MAPK signaling in relaying hsa_circRNA_0040462 mediated cell growth. Yet no specific pathway responsible for cell migration was popped up during bioinformatics prediction. We examined a panel of canonical markers characterizing cancer cell proliferation (Ki67, PI3K, AKT), migration (p65), stemness and anti-oxidant ability (p65, p-p65), yet these do not cover all possible mechanisms underlying the observed opposite roles of hsa_circRNA_0040462 on cell growth and migration that requires additional explorations. Further in-depth investigations on the functionalities of hsa_circRNA_0040462 during cancer progression and its phenotypic manifestations are necessary and left over for further studies.

## Conclusion

We report in this study hsa_circRNA_0040462 as a sensor of CAP treatment response that can be used to adjust the dose of CAP towards optimized therapeutic response and as a prognostic marker of tumors feasible for receiving CAP treatment. However, given the complexity of the regulatory network of a circular RNA, hsa_circRNA_0040462 is not a feasible therapeutic target as evidenced by its opposite functionalities on cancer cell proliferation and migration.

## Supplementary Material

Supplementary figures and tables.

## Figures and Tables

**Figure 7:**
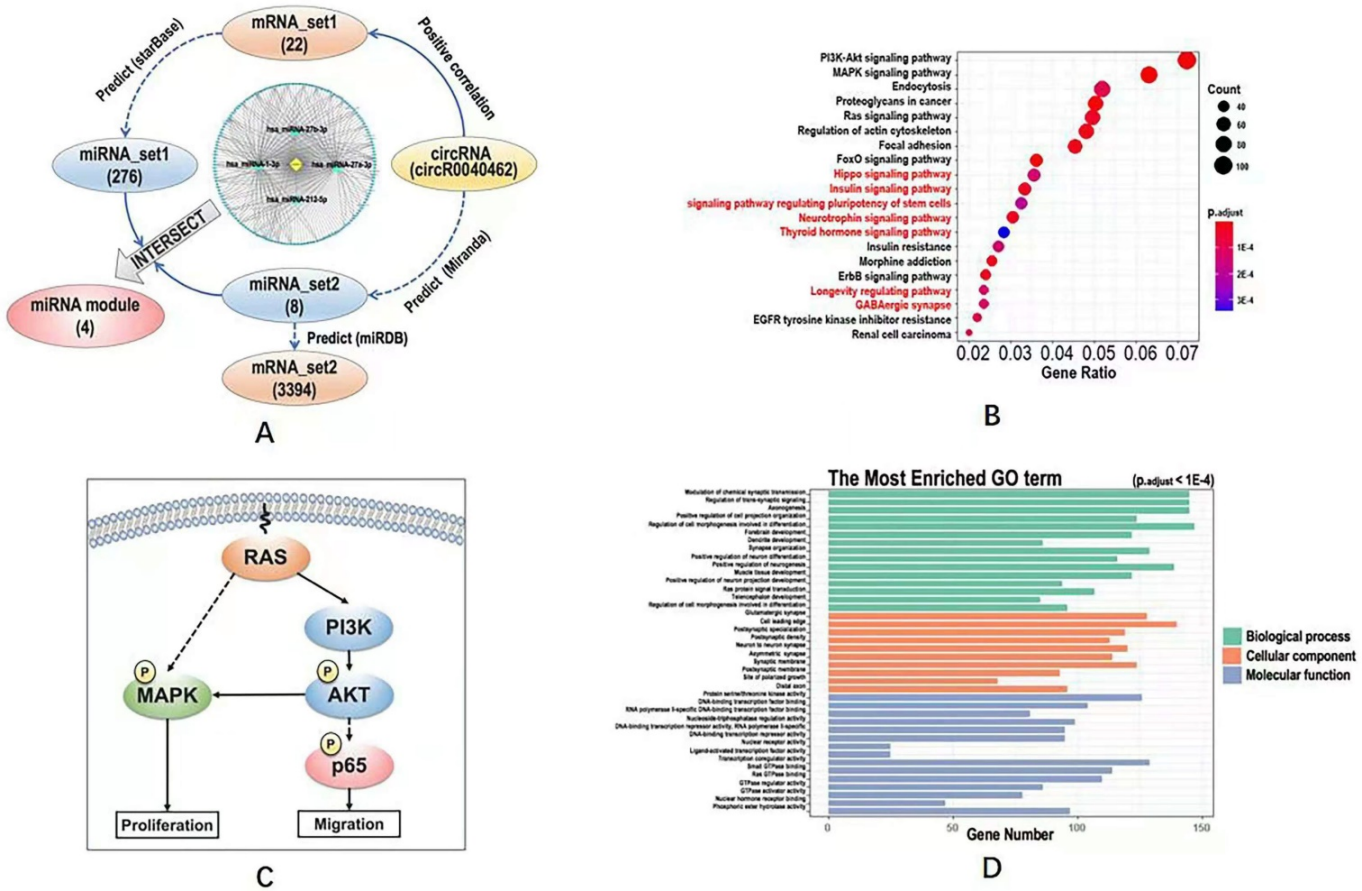
** Conceptual diagram illustrating the double-edged roles of hsa_circRNA_0040462 in tumor cell progression and the underlying mechanism.** One possible mechanism that leads to the double-edged manifestation of hsa_circRNA_0040462 on breast cancer cells is illustrated here. In this mechanism, hsa_circRNA_0040462 suppresses cell proliferation by inhibiting the RAS-PI3K/AKT, and RAS-MAPK pathway and promotes cell migration via promoting the phosphorylation of p65; CAP overrides the effect of hsa_circRNA_0040462 on cancer cell migration that leads to both halted cancer cell growth and migration. On the other hand, both hsa_circRNA_0040462 and CAP suppress the anti-oxidant ability of breast cancer cells in a pathway involving FOXO1; CAP could elevate the ROS level in breast cancer cells; elevated cellular ROS level and reduced anti-oxidant ability concomitantly occur in breast cancer cells that leads to their death.

**Table 1:** Information on antibodies used in this study

Product Name	Company	Catalog No.	Dilution ratio	Type
FoxO1 (C29H4) Rabbit mAb	CST	2880	1:1000	primary antibody
NF-κB p65 (D14E12) XP® Rabbit mAb	CST	8242S	1:1000	primary antibody
Phospho-NF-κB p65 (Ser536) (93H1) Rabbit mAb	CST	3033S	1:1000	primary antibody
Rabbit anti-Ki67 Polyclonal Antibody	Absin	Abs131303	1:1000	primary antibody
GAPDH Rabbit pAb	Abclonal	AC001	1:5000	primary antibody
HRP-labeled Goat Anti-Rabbit IgG(H+L)	Beyotime	A0208	1:1000	secondary antibody
