# Supplementary material for: Hsa_circRNA_0040462: a sensor of cells' response to CAP treatment with double-edged roles on breast cancer malignancy
Source: Int J Med Sci. 2022 Mar 21;19(4):640–50. doi: 10.7150/ijms.66940 (PMC9108402; doi:10.7150/ijms.66940)
Supplement: Supplementary file 1 — Supplementary figures and tables. [file ijmsv19p0640s1.pdf]

Supplementary materials

Supplementary Figure 1. KEGG pathways identified using mRNA_set1+2. The definitions of mRNA_set1, mRNA_set2, miRNA_set1, miRNA_set2 were illustrated in Figure 4A. The mRNA_set1+2 is the union of mRNA_set1 and mRNA_set2.

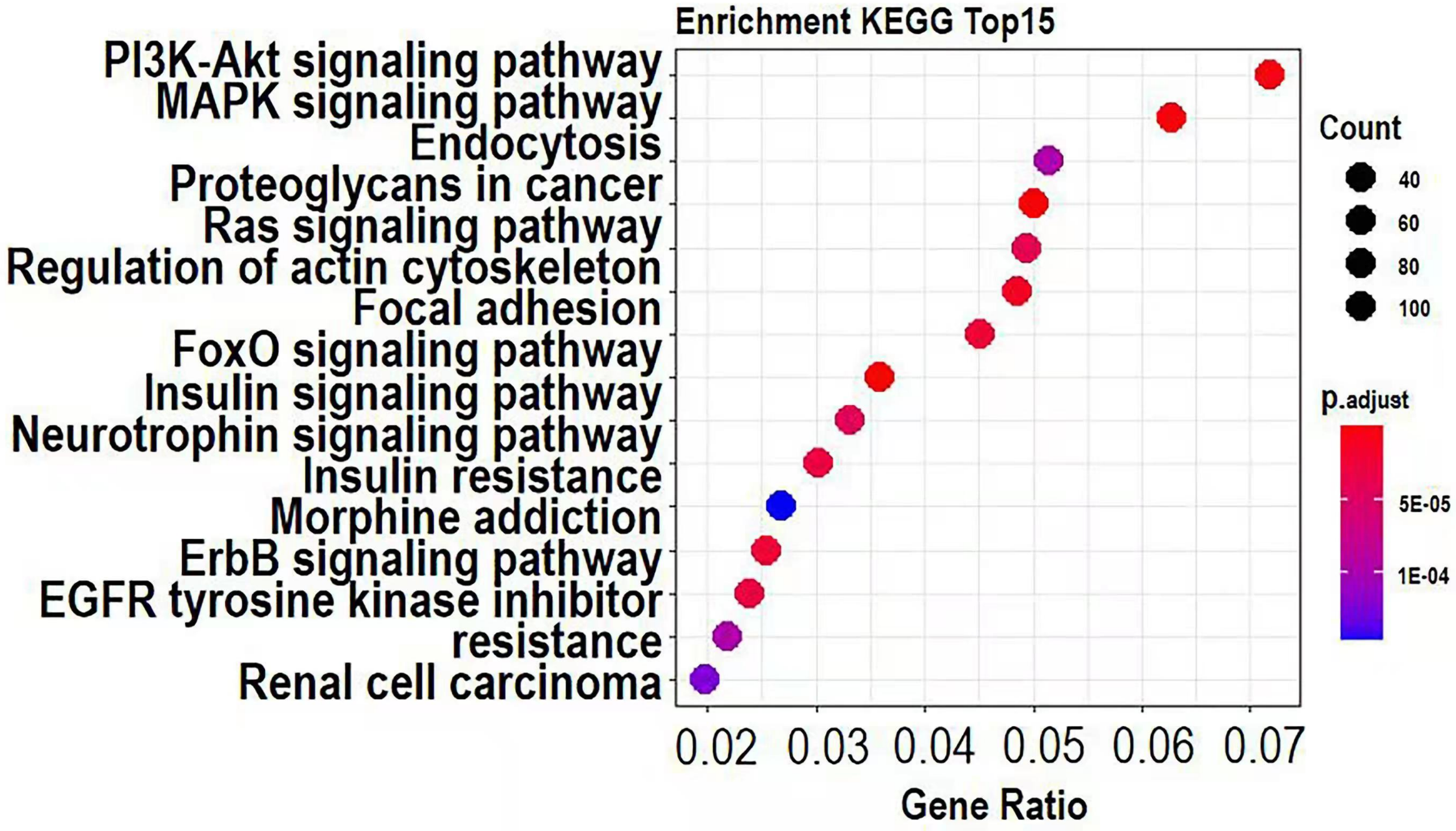

Supplementary Figure 2. Western blots examining the effect of hsa_circRNA_0040462 on key elements in the PI3K/AKT pathway. Phosphorylated PI3K, phosphorylated AKT and AKT total levels are represented as p-PI3K, p-AKT and AKT, respectively.

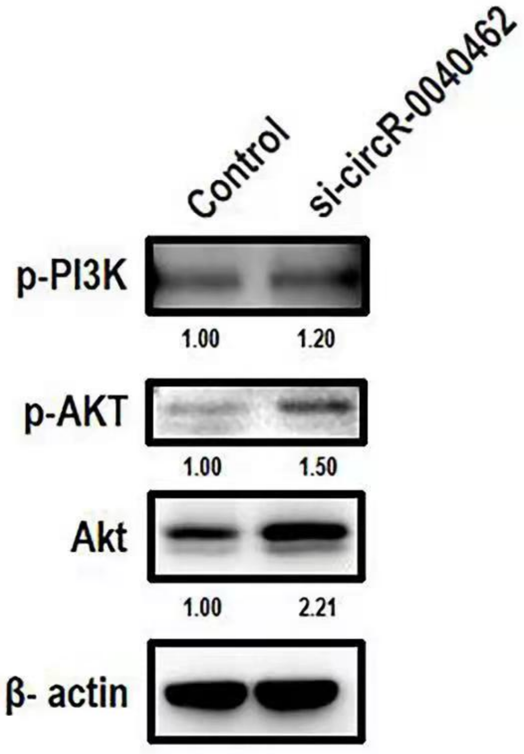

Supplementary Table 1. The panel of mRNAs predicted to be regulated by hsa_circRNA_0040462 from the whole transcriptome data and via miRDB prediction using miRNA_set2. Results obtained from the whole transcriptome data were denoted as 'set1', and from miRDB prediction using miRNA_set2 were denoted as 'mRNA_set2'. The definitions of mRNA_set1, mRNA_set2, miRNA_set1, miRNA_set2 were illustrated in Figure 4A.

GeneID	Data source	Correlation	P value	Prediction tool
LIN7B	mRNA_set1	0.92	3.67E-04	NA
ARL13A	mRNA_set1	0.91	6.46E-04	NA
CLEC7A	mRNA_set1	0.88	1.58E-03	NA
HEBP2	mRNA_set1	0.87	2.48E-03	NA
LIAS	mRNA_set1	0.86	2.75E-03	NA
EFHB	mRNA_set1	0.85	3.81E-03	NA
PTTG3P	mRNA_set1	0.84	4.24E-03	NA
GPR63	mRNA_set1	0.84	4.68E-03	NA
OCM	mRNA_set1	0.83	5.31E-03	NA
MDP1	mRNA_set1	0.83	5.33E-03	NA
RNU2-1	mRNA_set1	0.83	5.50E-03	NA
BNIPL	mRNA_set1	0.83	5.73E-03	NA
PCP2	mRNA_set1	0.82	6.61E-03	NA
C8orf4	mRNA_set1	0.82	6.77E-03	NA
KY	mRNA_set1	0.82	6.88E-03	NA
DOCK9-AS2	mRNA_set1	0.82	7.03E-03	NA
NXPH4	mRNA_set1	0.81	7.58E-03	NA
IFI27	mRNA_set1	0.81	7.73E-03	NA
GPR83	mRNA_set1	0.81	8.14E-03	NA
NIPSNAP3A	mRNA_set1	0.81	8.42E-03	NA
CHRM4	mRNA_set1	0.80	9.00E-03	NA
LPPR3	mRNA_set1	0.80	9.62E-03	NA
CDK19	mRNA_set2	NA	NA	miRDB
SOS2	mRNA_set2	NA	NA	miRDB
HACD3	mRNA_set2	NA	NA	miRDB
MMD	mRNA_set2	NA	NA	miRDB

SLC44A1	mRNA_set2	NA	NA	miRDB
AFF4	mRNA_set2	NA	NA	miRDB
GXYLT1	mRNA_set2	NA	NA	miRDB
ARFGEF1	mRNA_set2	NA	NA	miRDB
GCC2	mRNA_set2	NA	NA	miRDB
DCUN1D4	mRNA_set2	NA	NA	miRDB
PLK2	mRNA_set2	NA	NA	miRDB
TNPO1	mRNA_set2	NA	NA	miRDB
TRPV3	mRNA_set2	NA	NA	miRDB
GAB1	mRNA_set2	NA	NA	miRDB
RPS6KA5	mRNA_set2	NA	NA	miRDB
HOMER1	mRNA_set2	NA	NA	miRDB
WDR47	mRNA_set2	NA	NA	miRDB
LDLR	mRNA_set2	NA	NA	miRDB
BCL2L11	mRNA_set2	NA	NA	miRDB
UBE2D3	mRNA_set2	NA	NA	miRDB
NPTN	mRNA_set2	NA	NA	miRDB
MEOX2	mRNA_set2	NA	NA	miRDB
CBLB	mRNA_set2	NA	NA	miRDB
KMT2A	mRNA_set2	NA	NA	miRDB
PAX7	mRNA_set2	NA	NA	miRDB
CDK14	mRNA_set2	NA	NA	miRDB
CPED1	mRNA_set2	NA	NA	miRDB
SMIM14	mRNA_set2	NA	NA	miRDB
GJA1	mRNA_set2	NA	NA	miRDB
TAGLN2	mRNA_set2	NA	NA	miRDB
TNKS2	mRNA_set2	NA	NA	miRDB
XPO6	mRNA_set2	NA	NA	miRDB

GLCCI1	mRNA_set2	NA	NA	miRDB
LSM8	mRNA_set2	NA	NA	miRDB
NTF3	mRNA_set2	NA	NA	miRDB
CREBZF	mRNA_set2	NA	NA	miRDB
SEMA3A	mRNA_set2	NA	NA	miRDB
GRIA4	mRNA_set2	NA	NA	miRDB
BEX3	mRNA_set2	NA	NA	miRDB
MBTD1	mRNA_set2	NA	NA	miRDB
PDS5B	mRNA_set2	NA	NA	miRDB
AKIRIN1	mRNA_set2	NA	NA	miRDB
RGPD6	mRNA_set2	NA	NA	miRDB
PHLPP2	mRNA_set2	NA	NA	miRDB
TAB3	mRNA_set2	NA	NA	miRDB
SOS1	mRNA_set2	NA	NA	miRDB
FBXW7	mRNA_set2	NA	NA	miRDB
CDS1	mRNA_set2	NA	NA	miRDB
RGPD5	mRNA_set2	NA	NA	miRDB
SEMA6A	mRNA_set2	NA	NA	miRDB
NRK	mRNA_set2	NA	NA	miRDB
DENND4C	mRNA_set2	NA	NA	miRDB
ROBO1	mRNA_set2	NA	NA	miRDB
FXR1	mRNA_set2	NA	NA	miRDB
ZFYVE26	mRNA_set2	NA	NA	miRDB
UHMK1	mRNA_set2	NA	NA	miRDB
C5orf30	mRNA_set2	NA	NA	miRDB
SZRD1	mRNA_set2	NA	NA	miRDB
AKAP1	mRNA_set2	NA	NA	miRDB
B4GALT6	mRNA_set2	NA	NA	miRDB

TWF1	mRNA_set2	NA	NA	miRDB
SS18	mRNA_set2	NA	NA	miRDB
SNX2	mRNA_set2	NA	NA	miRDB
SLC39A10	mRNA_set2	NA	NA	miRDB
ZNF281	mRNA_set2	NA	NA	miRDB
PHAX	mRNA_set2	NA	NA	miRDB
KCNIP3	mRNA_set2	NA	NA	miRDB
SULF1	mRNA_set2	NA	NA	miRDB
PDIK1L	mRNA_set2	NA	NA	miRDB
PTPRJ	mRNA_set2	NA	NA	miRDB
MKX	mRNA_set2	NA	NA	miRDB
XK	mRNA_set2	NA	NA	miRDB
TMTC2	mRNA_set2	NA	NA	miRDB
PTPN4	mRNA_set2	NA	NA	miRDB
USP42	mRNA_set2	NA	NA	miRDB
ABHD17C	mRNA_set2	NA	NA	miRDB
GPAM	mRNA_set2	NA	NA	miRDB
ARHGEF26	mRNA_set2	NA	NA	miRDB
RGPD4	mRNA_set2	NA	NA	miRDB
KCNK2	mRNA_set2	NA	NA	miRDB
EYA1	mRNA_set2	NA	NA	miRDB
RGPD8	mRNA_set2	NA	NA	miRDB
ZBTB34	mRNA_set2	NA	NA	miRDB
KIAA1109	mRNA_set2	NA	NA	miRDB
ST6GALNAC3	mRNA_set2	NA	NA	miRDB
EYA4	mRNA_set2	NA	NA	miRDB
PLEKHJ1	mRNA_set2	NA	NA	miRDB
MAP3K9	mRNA_set2	NA	NA	miRDB

MED12L	mRNA_set2	NA	NA	miRDB
BTAF1	mRNA_set2	NA	NA	miRDB
NPEPL1	mRNA_set2	NA	NA	miRDB
BICC1	mRNA_set2	NA	NA	miRDB
JMY	mRNA_set2	NA	NA	miRDB
PHF3	mRNA_set2	NA	NA	miRDB
ATP6AP2	mRNA_set2	NA	NA	miRDB
USP33	mRNA_set2	NA	NA	miRDB
ARHGAP21	mRNA_set2	NA	NA	miRDB
VPS37A	mRNA_set2	NA	NA	miRDB
ABCB7	mRNA_set2	NA	NA	miRDB
RMND5A	mRNA_set2	NA	NA	miRDB
B4GALT5	mRNA_set2	NA	NA	miRDB
DCP2	mRNA_set2	NA	NA	miRDB
QKI	mRNA_set2	NA	NA	miRDB
KAT7	mRNA_set2	NA	NA	miRDB
ZDHHC7	mRNA_set2	NA	NA	miRDB
OSBPL11	mRNA_set2	NA	NA	miRDB
TMEM9B	mRNA_set2	NA	NA	miRDB
CORO1C	mRNA_set2	NA	NA	miRDB
UTRN	mRNA_set2	NA	NA	miRDB
G6PD	mRNA_set2	NA	NA	miRDB
TMCC1	mRNA_set2	NA	NA	miRDB
SLC10A7	mRNA_set2	NA	NA	miRDB
GPR137C	mRNA_set2	NA	NA	miRDB
MMD2	mRNA_set2	NA	NA	miRDB
UNC119B	mRNA_set2	NA	NA	miRDB
CEBPZ	mRNA_set2	NA	NA	miRDB

TPPP	mRNA_set2	NA	NA	miRDB
BDNF	mRNA_set2	NA	NA	miRDB
NFATC3	mRNA_set2	NA	NA	miRDB
PIK3C2B	mRNA_set2	NA	NA	miRDB
BTBD11	mRNA_set2	NA	NA	miRDB
PHF20L1	mRNA_set2	NA	NA	miRDB
EYS	mRNA_set2	NA	NA	miRDB
EIF3A	mRNA_set2	NA	NA	miRDB
NDNF	mRNA_set2	NA	NA	miRDB
PCDH19	mRNA_set2	NA	NA	miRDB
FAM210B	mRNA_set2	NA	NA	miRDB
MTM1	mRNA_set2	NA	NA	miRDB
KRAS	mRNA_set2	NA	NA	miRDB
SGPP1	mRNA_set2	NA	NA	miRDB
LYN	mRNA_set2	NA	NA	miRDB
TM6SF1	mRNA_set2	NA	NA	miRDB
USP32	mRNA_set2	NA	NA	miRDB
CREBL2	mRNA_set2	NA	NA	miRDB
MYBL1	mRNA_set2	NA	NA	miRDB
SBF2	mRNA_set2	NA	NA	miRDB
PRR3	mRNA_set2	NA	NA	miRDB
ONECUT2	mRNA_set2	NA	NA	miRDB
TMBIM6	mRNA_set2	NA	NA	miRDB
FOXA3	mRNA_set2	NA	NA	miRDB
BTG2	mRNA_set2	NA	NA	miRDB
ADAMTSL3	mRNA_set2	NA	NA	miRDB
RUNX1	mRNA_set2	NA	NA	miRDB
ACVR1C	mRNA_set2	NA	NA	miRDB

TMEM170B	mRNA_set2	NA	NA	miRDB
EHF	mRNA_set2	NA	NA	miRDB
GRIN2D	mRNA_set2	NA	NA	miRDB
USP46	mRNA_set2	NA	NA	miRDB
C2CD2	mRNA_set2	NA	NA	miRDB
WNK3	mRNA_set2	NA	NA	miRDB
HOXA5	mRNA_set2	NA	NA	miRDB
SLC7A11	mRNA_set2	NA	NA	miRDB
RNF139	mRNA_set2	NA	NA	miRDB
GATC	mRNA_set2	NA	NA	miRDB
DNAJC27	mRNA_set2	NA	NA	miRDB
KDM7A	mRNA_set2	NA	NA	miRDB
KIAA1217	mRNA_set2	NA	NA	miRDB
ESR1	mRNA_set2	NA	NA	miRDB
ADAMTS19	mRNA_set2	NA	NA	miRDB
BTBD3	mRNA_set2	NA	NA	miRDB
TGFA	mRNA_set2	NA	NA	miRDB
NEURL4	mRNA_set2	NA	NA	miRDB
DNMT1	mRNA_set2	NA	NA	miRDB
PPP1R10	mRNA_set2	NA	NA	miRDB
TNRC6A	mRNA_set2	NA	NA	miRDB
INO80	mRNA_set2	NA	NA	miRDB
CCT6A	mRNA_set2	NA	NA	miRDB
GADD45A	mRNA_set2	NA	NA	miRDB
TNRC6C	mRNA_set2	NA	NA	miRDB
GTF2H1	mRNA_set2	NA	NA	miRDB
HOXC8	mRNA_set2	NA	NA	miRDB
CLCN6	mRNA_set2	NA	NA	miRDB

ERBB3	mRNA_set2	NA	NA	miRDB
PAX3	mRNA_set2	NA	NA	miRDB
PDCD4	mRNA_set2	NA	NA	miRDB
FOXP1	mRNA_set2	NA	NA	miRDB
KAT6A	mRNA_set2	NA	NA	miRDB
TRAPPC3	mRNA_set2	NA	NA	miRDB
ADAR	mRNA_set2	NA	NA	miRDB
CAAP1	mRNA_set2	NA	NA	miRDB
FAM102A	mRNA_set2	NA	NA	miRDB
LRRC8A	mRNA_set2	NA	NA	miRDB
C5orf51	mRNA_set2	NA	NA	miRDB
FAM168A	mRNA_set2	NA	NA	miRDB
AP1G1	mRNA_set2	NA	NA	miRDB
NXT2	mRNA_set2	NA	NA	miRDB
KIF2A	mRNA_set2	NA	NA	miRDB
PDCD10	mRNA_set2	NA	NA	miRDB
POGZ	mRNA_set2	NA	NA	miRDB
CLOCK	mRNA_set2	NA	NA	miRDB
PPARGC1A	mRNA_set2	NA	NA	miRDB
MBNL2	mRNA_set2	NA	NA	miRDB
ADAMTS6	mRNA_set2	NA	NA	miRDB
ITGB8	mRNA_set2	NA	NA	miRDB
NEK10	mRNA_set2	NA	NA	miRDB
GDF10	mRNA_set2	NA	NA	miRDB
ARHGEF3	mRNA_set2	NA	NA	miRDB
THBS2	mRNA_set2	NA	NA	miRDB
RORA	mRNA_set2	NA	NA	miRDB
PNKD	mRNA_set2	NA	NA	miRDB

KCTD8	mRNA_set2	NA	NA	miRDB
SLITRK1	mRNA_set2	NA	NA	miRDB
PDIA5	mRNA_set2	NA	NA	miRDB
ABCA1	mRNA_set2	NA	NA	miRDB
EDRF1	mRNA_set2	NA	NA	miRDB
KLF3	mRNA_set2	NA	NA	miRDB
SMAD9	mRNA_set2	NA	NA	miRDB
TFAP2B	mRNA_set2	NA	NA	miRDB
HIPK2	mRNA_set2	NA	NA	miRDB
UNKL	mRNA_set2	NA	NA	miRDB
PLEKHH1	mRNA_set2	NA	NA	miRDB
PLCL2	mRNA_set2	NA	NA	miRDB
ROR1	mRNA_set2	NA	NA	miRDB
SEMA7A	mRNA_set2	NA	NA	miRDB
CKAP4	mRNA_set2	NA	NA	miRDB
VAV2	mRNA_set2	NA	NA	miRDB
CDR2	mRNA_set2	NA	NA	miRDB
FBXO10	mRNA_set2	NA	NA	miRDB
LIFR	mRNA_set2	NA	NA	miRDB
ABHD6	mRNA_set2	NA	NA	miRDB
ZFHX3	mRNA_set2	NA	NA	miRDB
ADORA2B	mRNA_set2	NA	NA	miRDB
GOLM1	mRNA_set2	NA	NA	miRDB
EPS8	mRNA_set2	NA	NA	miRDB
CCNK	mRNA_set2	NA	NA	miRDB
UGCG	mRNA_set2	NA	NA	miRDB
IPMK	mRNA_set2	NA	NA	miRDB
HIVEP3	mRNA_set2	NA	NA	miRDB

PEG10	mRNA_set2	NA	NA	miRDB
SLC35F1	mRNA_set2	NA	NA	miRDB
ZHX1	mRNA_set2	NA	NA	miRDB
GNS	mRNA_set2	NA	NA	miRDB
KIAA0232	mRNA_set2	NA	NA	miRDB
POU3F2	mRNA_set2	NA	NA	miRDB
XPO4	mRNA_set2	NA	NA	miRDB
ARL6IP1	mRNA_set2	NA	NA	miRDB
GLRX5	mRNA_set2	NA	NA	miRDB
FAM168B	mRNA_set2	NA	NA	miRDB
ERRFI1	mRNA_set2	NA	NA	miRDB
MXD1	mRNA_set2	NA	NA	miRDB
AGO4	mRNA_set2	NA	NA	miRDB
YWHAB	mRNA_set2	NA	NA	miRDB
COL4A1	mRNA_set2	NA	NA	miRDB
LIPA	mRNA_set2	NA	NA	miRDB
CABP7	mRNA_set2	NA	NA	miRDB
LMTK2	mRNA_set2	NA	NA	miRDB
INHBB	mRNA_set2	NA	NA	miRDB
HSP90B1	mRNA_set2	NA	NA	miRDB
FNDC3A	mRNA_set2	NA	NA	miRDB
FBXO33	mRNA_set2	NA	NA	miRDB
IMPACT	mRNA_set2	NA	NA	miRDB
EIF4E	mRNA_set2	NA	NA	miRDB
MAP4K3	mRNA_set2	NA	NA	miRDB
SPRED1	mRNA_set2	NA	NA	miRDB
SLC25A22	mRNA_set2	NA	NA	miRDB
UST	mRNA_set2	NA	NA	miRDB

GPD2	mRNA_set2	NA	NA	miRDB
MCHR1	mRNA_set2	NA	NA	miRDB
CD2AP	mRNA_set2	NA	NA	miRDB
CBL	mRNA_set2	NA	NA	miRDB
HIGD1A	mRNA_set2	NA	NA	miRDB
EIF1AX	mRNA_set2	NA	NA	miRDB
GNPDA2	mRNA_set2	NA	NA	miRDB
ANKRD29	mRNA_set2	NA	NA	miRDB
TMEM243	mRNA_set2	NA	NA	miRDB
TCEANC2	mRNA_set2	NA	NA	miRDB
RHOBTB1	mRNA_set2	NA	NA	miRDB
DDX3X	mRNA_set2	NA	NA	miRDB
VSTM2A	mRNA_set2	NA	NA	miRDB
TOPORS	mRNA_set2	NA	NA	miRDB
PAXIP1	mRNA_set2	NA	NA	miRDB
MIA2	mRNA_set2	NA	NA	miRDB
TSHZ3	mRNA_set2	NA	NA	miRDB
PI15	mRNA_set2	NA	NA	miRDB
YOD1	mRNA_set2	NA	NA	miRDB
LMAN1	mRNA_set2	NA	NA	miRDB
STRN	mRNA_set2	NA	NA	miRDB
MIGA1	mRNA_set2	NA	NA	miRDB
FUT9	mRNA_set2	NA	NA	miRDB
CARNMT1	mRNA_set2	NA	NA	miRDB
PTAR1	mRNA_set2	NA	NA	miRDB
INO80D	mRNA_set2	NA	NA	miRDB
NAA30	mRNA_set2	NA	NA	miRDB
MOB1A	mRNA_set2	NA	NA	miRDB

COL1A2	mRNA_set2	NA	NA	miRDB
BRWD1	mRNA_set2	NA	NA	miRDB
PPP6C	mRNA_set2	NA	NA	miRDB
PTPN12	mRNA_set2	NA	NA	miRDB
MARK1	mRNA_set2	NA	NA	miRDB
ZNF800	mRNA_set2	NA	NA	miRDB
RREB1	mRNA_set2	NA	NA	miRDB
PDE7B	mRNA_set2	NA	NA	miRDB
C20orf194	mRNA_set2	NA	NA	miRDB
CREBRF	mRNA_set2	NA	NA	miRDB
DNAJC13	mRNA_set2	NA	NA	miRDB
TSC22D2	mRNA_set2	NA	NA	miRDB
GSPT1	mRNA_set2	NA	NA	miRDB
B4GALT3	mRNA_set2	NA	NA	miRDB
CCNG1	mRNA_set2	NA	NA	miRDB
NR2F6	mRNA_set2	NA	NA	miRDB
ITSN2	mRNA_set2	NA	NA	miRDB
CSRP2	mRNA_set2	NA	NA	miRDB
NECAP1	mRNA_set2	NA	NA	miRDB
ID4	mRNA_set2	NA	NA	miRDB
RO60	mRNA_set2	NA	NA	miRDB
RALGAPA2	mRNA_set2	NA	NA	miRDB
MAP1B	mRNA_set2	NA	NA	miRDB
GRM5	mRNA_set2	NA	NA	miRDB
ST3GAL6	mRNA_set2	NA	NA	miRDB
ASPH	mRNA_set2	NA	NA	miRDB
BRPF3	mRNA_set2	NA	NA	miRDB
SSH1	mRNA_set2	NA	NA	miRDB

STYK1	mRNA_set2	NA	NA	miRDB
KDM3A	mRNA_set2	NA	NA	miRDB
COG7	mRNA_set2	NA	NA	miRDB
EDEM3	mRNA_set2	NA	NA	miRDB
SLC25A25	mRNA_set2	NA	NA	miRDB
SNRNP27	mRNA_set2	NA	NA	miRDB
DDX6	mRNA_set2	NA	NA	miRDB
TENT2	mRNA_set2	NA	NA	miRDB
ROBO2	mRNA_set2	NA	NA	miRDB
SMS	mRNA_set2	NA	NA	miRDB
MRGPRX3	mRNA_set2	NA	NA	miRDB
CAND1	mRNA_set2	NA	NA	miRDB
RNF38	mRNA_set2	NA	NA	miRDB
ARL8B	mRNA_set2	NA	NA	miRDB
WNT1	mRNA_set2	NA	NA	miRDB
KRT76	mRNA_set2	NA	NA	miRDB
ADGRB3	mRNA_set2	NA	NA	miRDB
LRP2	mRNA_set2	NA	NA	miRDB
LEPROTL1	mRNA_set2	NA	NA	miRDB
FBN1	mRNA_set2	NA	NA	miRDB
EOGT	mRNA_set2	NA	NA	miRDB
NRP1	mRNA_set2	NA	NA	miRDB
RALBP1	mRNA_set2	NA	NA	miRDB
SESN2	mRNA_set2	NA	NA	miRDB
SESTD1	mRNA_set2	NA	NA	miRDB
BAZ2B	mRNA_set2	NA	NA	miRDB
ZFP36L2	mRNA_set2	NA	NA	miRDB
MATR3	mRNA_set2	NA	NA	miRDB

ATP6V1A	mRNA_set2	NA	NA	miRDB
TMEM178A	mRNA_set2	NA	NA	miRDB
API5	mRNA_set2	NA	NA	miRDB
HELZ2	mRNA_set2	NA	NA	miRDB
WDR48	mRNA_set2	NA	NA	miRDB
C2orf69	mRNA_set2	NA	NA	miRDB
TMSB4X	mRNA_set2	NA	NA	miRDB
EDN1	mRNA_set2	NA	NA	miRDB
MFSD14A	mRNA_set2	NA	NA	miRDB
MON2	mRNA_set2	NA	NA	miRDB
STARD7	mRNA_set2	NA	NA	miRDB
MAPRE2	mRNA_set2	NA	NA	miRDB
CBLN2	mRNA_set2	NA	NA	miRDB
MEX3C	mRNA_set2	NA	NA	miRDB
NPY2R	mRNA_set2	NA	NA	miRDB
FAM122C	mRNA_set2	NA	NA	miRDB
CEP44	mRNA_set2	NA	NA	miRDB
PPM1D	mRNA_set2	NA	NA	miRDB
ACBD5	mRNA_set2	NA	NA	miRDB
PSMD7	mRNA_set2	NA	NA	miRDB
C1QBP	mRNA_set2	NA	NA	miRDB
STXBP5	mRNA_set2	NA	NA	miRDB
PPP1R15B	mRNA_set2	NA	NA	miRDB
PIAS1	mRNA_set2	NA	NA	miRDB
ZBTB10	mRNA_set2	NA	NA	miRDB
FBXO28	mRNA_set2	NA	NA	miRDB
KCNQ5	mRNA_set2	NA	NA	miRDB
PAPOLA	mRNA_set2	NA	NA	miRDB

CXADR	mRNA_set2	NA	NA	miRDB
ZDHHC17	mRNA_set2	NA	NA	miRDB
PREX2	mRNA_set2	NA	NA	miRDB
OSBP	mRNA_set2	NA	NA	miRDB
DOT1L	mRNA_set2	NA	NA	miRDB
KIAA1211L	mRNA_set2	NA	NA	miRDB
INSM2	mRNA_set2	NA	NA	miRDB
ZCCHC24	mRNA_set2	NA	NA	miRDB
GALNT7	mRNA_set2	NA	NA	miRDB
B3GNT7	mRNA_set2	NA	NA	miRDB
APPBP2	mRNA_set2	NA	NA	miRDB
PPARG	mRNA_set2	NA	NA	miRDB
UBE2V1	mRNA_set2	NA	NA	miRDB
SHE	mRNA_set2	NA	NA	miRDB
NIPAL4	mRNA_set2	NA	NA	miRDB
ARF3	mRNA_set2	NA	NA	miRDB
CTH	mRNA_set2	NA	NA	miRDB
KCNA4	mRNA_set2	NA	NA	miRDB
ENDOU	mRNA_set2	NA	NA	miRDB
PCNX1	mRNA_set2	NA	NA	miRDB
TNRC18	mRNA_set2	NA	NA	miRDB
PTGER3	mRNA_set2	NA	NA	miRDB
KITLG	mRNA_set2	NA	NA	miRDB
USP25	mRNA_set2	NA	NA	miRDB
ZSCAN26	mRNA_set2	NA	NA	miRDB
NPAS3	mRNA_set2	NA	NA	miRDB
SLC39A11	mRNA_set2	NA	NA	miRDB
GRIA3	mRNA_set2	NA	NA	miRDB

PCDH9	mRNA_set2	NA	NA	miRDB
AGFG1	mRNA_set2	NA	NA	miRDB
VSIG10	mRNA_set2	NA	NA	miRDB
MIER3	mRNA_set2	NA	NA	miRDB
STARD13	mRNA_set2	NA	NA	miRDB
ZDHHC23	mRNA_set2	NA	NA	miRDB
FAM43A	mRNA_set2	NA	NA	miRDB
ACVR1	mRNA_set2	NA	NA	miRDB
TBC1D8	mRNA_set2	NA	NA	miRDB
RAB34	mRNA_set2	NA	NA	miRDB
ESRRG	mRNA_set2	NA	NA	miRDB
ATG14	mRNA_set2	NA	NA	miRDB
PNPLA6	mRNA_set2	NA	NA	miRDB
LBR	mRNA_set2	NA	NA	miRDB
SNX27	mRNA_set2	NA	NA	miRDB
PLAA	mRNA_set2	NA	NA	miRDB
ATP11A	mRNA_set2	NA	NA	miRDB
RASSF8	mRNA_set2	NA	NA	miRDB
KLF6	mRNA_set2	NA	NA	miRDB
TMED7	mRNA_set2	NA	NA	miRDB
CCDC6	mRNA_set2	NA	NA	miRDB
GMFB	mRNA_set2	NA	NA	miRDB
PHF20	mRNA_set2	NA	NA	miRDB
SGCB	mRNA_set2	NA	NA	miRDB
ADAM22	mRNA_set2	NA	NA	miRDB
SRSF9	mRNA_set2	NA	NA	miRDB
TRA2B	mRNA_set2	NA	NA	miRDB
FAM155A	mRNA_set2	NA	NA	miRDB

DDX5	mRNA_set2	NA	NA	miRDB
SRI	mRNA_set2	NA	NA	miRDB
CTTNBP2NL	mRNA_set2	NA	NA	miRDB
POGK	mRNA_set2	NA	NA	miRDB
FRS2	mRNA_set2	NA	NA	miRDB
RARB	mRNA_set2	NA	NA	miRDB
CAVIN2	mRNA_set2	NA	NA	miRDB
HSPD1	mRNA_set2	NA	NA	miRDB
MYOCD	mRNA_set2	NA	NA	miRDB
IFT52	mRNA_set2	NA	NA	miRDB
HS3ST3B1	mRNA_set2	NA	NA	miRDB
CTDSP2	mRNA_set2	NA	NA	miRDB
CNR1	mRNA_set2	NA	NA	miRDB
SDC1	mRNA_set2	NA	NA	miRDB
ZNF316	mRNA_set2	NA	NA	miRDB
PIKFYVE	mRNA_set2	NA	NA	miRDB
AMER2	mRNA_set2	NA	NA	miRDB
IRF2BPL	mRNA_set2	NA	NA	miRDB
DDX3Y	mRNA_set2	NA	NA	miRDB
SPOCK3	mRNA_set2	NA	NA	miRDB
TRIM33	mRNA_set2	NA	NA	miRDB
KATNA1	mRNA_set2	NA	NA	miRDB
HHAT	mRNA_set2	NA	NA	miRDB
SLC25A33	mRNA_set2	NA	NA	miRDB
SAMD8	mRNA_set2	NA	NA	miRDB
CCDC125	mRNA_set2	NA	NA	miRDB
CAV2	mRNA_set2	NA	NA	miRDB
VLDLR	mRNA_set2	NA	NA	miRDB

MEIOC	mRNA_set2	NA	NA	miRDB
LATS2	mRNA_set2	NA	NA	miRDB
GNPTAB	mRNA_set2	NA	NA	miRDB
DTNA	mRNA_set2	NA	NA	miRDB
KIF5B	mRNA_set2	NA	NA	miRDB
SNX18	mRNA_set2	NA	NA	miRDB
NUP205	mRNA_set2	NA	NA	miRDB
NRIP1	mRNA_set2	NA	NA	miRDB
TEAD1	mRNA_set2	NA	NA	miRDB
C1orf52	mRNA_set2	NA	NA	miRDB
ADAMTS10	mRNA_set2	NA	NA	miRDB
SLC6A1	mRNA_set2	NA	NA	miRDB
HMGCR	mRNA_set2	NA	NA	miRDB
PALM2	mRNA_set2	NA	NA	miRDB
GAREM1	mRNA_set2	NA	NA	miRDB
HBEGF	mRNA_set2	NA	NA	miRDB
REPS1	mRNA_set2	NA	NA	miRDB
EPB41L4A	mRNA_set2	NA	NA	miRDB
SFRP1	mRNA_set2	NA	NA	miRDB
CREB1	mRNA_set2	NA	NA	miRDB
TPR	mRNA_set2	NA	NA	miRDB
ELFN2	mRNA_set2	NA	NA	miRDB
FAM184A	mRNA_set2	NA	NA	miRDB
ATAD2B	mRNA_set2	NA	NA	miRDB
STAB2	mRNA_set2	NA	NA	miRDB
MTURN	mRNA_set2	NA	NA	miRDB
PAQR9	mRNA_set2	NA	NA	miRDB
DIPK1A	mRNA_set2	NA	NA	miRDB

MKNK2	mRNA_set2	NA	NA	miRDB
FBXO34	mRNA_set2	NA	NA	miRDB
PARD6B	mRNA_set2	NA	NA	miRDB
ARHGAP32	mRNA_set2	NA	NA	miRDB
STAG1	mRNA_set2	NA	NA	miRDB
NABP1	mRNA_set2	NA	NA	miRDB
RPS6KB1	mRNA_set2	NA	NA	miRDB
TRIM23	mRNA_set2	NA	NA	miRDB
RCAN2	mRNA_set2	NA	NA	miRDB
ACTA2	mRNA_set2	NA	NA	miRDB
HOXA10	mRNA_set2	NA	NA	miRDB
MATN3	mRNA_set2	NA	NA	miRDB
TGIF2	mRNA_set2	NA	NA	miRDB
RBM24	mRNA_set2	NA	NA	miRDB
DOCK6	mRNA_set2	NA	NA	miRDB
DICER1	mRNA_set2	NA	NA	miRDB
SIK1	mRNA_set2	NA	NA	miRDB
SLC24A3	mRNA_set2	NA	NA	miRDB
F3	mRNA_set2	NA	NA	miRDB
DMXL1	mRNA_set2	NA	NA	miRDB
VSIG1	mRNA_set2	NA	NA	miRDB
GRID2	mRNA_set2	NA	NA	miRDB
SKIDA1	mRNA_set2	NA	NA	miRDB
SLC25A44	mRNA_set2	NA	NA	miRDB
JARID2	mRNA_set2	NA	NA	miRDB
SIK1B	mRNA_set2	NA	NA	miRDB
PPP6R1	mRNA_set2	NA	NA	miRDB
E2F7	mRNA_set2	NA	NA	miRDB

HECW2	mRNA_set2	NA	NA	miRDB
CS	mRNA_set2	NA	NA	miRDB
SNAP91	mRNA_set2	NA	NA	miRDB
CDKN1B	mRNA_set2	NA	NA	miRDB
C1GALT1	mRNA_set2	NA	NA	miRDB
FAM104A	mRNA_set2	NA	NA	miRDB
CYTH3	mRNA_set2	NA	NA	miRDB
HMBOX1	mRNA_set2	NA	NA	miRDB
CNN3	mRNA_set2	NA	NA	miRDB
STAG2	mRNA_set2	NA	NA	miRDB
KTN1	mRNA_set2	NA	NA	miRDB
SLC39A9	mRNA_set2	NA	NA	miRDB
CLTC	mRNA_set2	NA	NA	miRDB
PIK3C2A	mRNA_set2	NA	NA	miRDB
ATF2	mRNA_set2	NA	NA	miRDB
KCTD10	mRNA_set2	NA	NA	miRDB
SLC25A30	mRNA_set2	NA	NA	miRDB
PLXNA4	mRNA_set2	NA	NA	miRDB
MPP5	mRNA_set2	NA	NA	miRDB
STC2	mRNA_set2	NA	NA	miRDB
HP1BP3	mRNA_set2	NA	NA	miRDB
GLCE	mRNA_set2	NA	NA	miRDB
SEC22B	mRNA_set2	NA	NA	miRDB
PCDHB13	mRNA_set2	NA	NA	miRDB
PLPPR4	mRNA_set2	NA	NA	miRDB
SRGAP2	mRNA_set2	NA	NA	miRDB
CCDC117	mRNA_set2	NA	NA	miRDB
ATF6	mRNA_set2	NA	NA	miRDB

MYPN	mRNA_set2	NA	NA	miRDB
PRDM10	mRNA_set2	NA	NA	miRDB
DCAF12	mRNA_set2	NA	NA	miRDB
MACO1	mRNA_set2	NA	NA	miRDB
RALGDS	mRNA_set2	NA	NA	miRDB
FAM20B	mRNA_set2	NA	NA	miRDB
SMG1	mRNA_set2	NA	NA	miRDB
PDZK1	mRNA_set2	NA	NA	miRDB
ME1	mRNA_set2	NA	NA	miRDB
DFFB	mRNA_set2	NA	NA	miRDB
CHMP5	mRNA_set2	NA	NA	miRDB
TMEM200B	mRNA_set2	NA	NA	miRDB
TMEM206	mRNA_set2	NA	NA	miRDB
FLG	mRNA_set2	NA	NA	miRDB
SLK	mRNA_set2	NA	NA	miRDB
SEC61G	mRNA_set2	NA	NA	miRDB
NAV3	mRNA_set2	NA	NA	miRDB
PRPF40A	mRNA_set2	NA	NA	miRDB
ACOT13	mRNA_set2	NA	NA	miRDB
MMS22L	mRNA_set2	NA	NA	miRDB
SCRN1	mRNA_set2	NA	NA	miRDB
RAPH1	mRNA_set2	NA	NA	miRDB
NPEPPS	mRNA_set2	NA	NA	miRDB
LPAR6	mRNA_set2	NA	NA	miRDB
CLCN3	mRNA_set2	NA	NA	miRDB
PANK1	mRNA_set2	NA	NA	miRDB
RNF141	mRNA_set2	NA	NA	miRDB
TLK2	mRNA_set2	NA	NA	miRDB

YWHAQ	mRNA_set2	NA	NA	miRDB
LPCAT1	mRNA_set2	NA	NA	miRDB
MAP2K4	mRNA_set2	NA	NA	miRDB
ING5	mRNA_set2	NA	NA	miRDB
AK4	mRNA_set2	NA	NA	miRDB
NEMP2	mRNA_set2	NA	NA	miRDB
CACNG2	mRNA_set2	NA	NA	miRDB
NR5A2	mRNA_set2	NA	NA	miRDB
CEP135	mRNA_set2	NA	NA	miRDB
BRWD3	mRNA_set2	NA	NA	miRDB
SNAP25	mRNA_set2	NA	NA	miRDB
MYT1	mRNA_set2	NA	NA	miRDB
LPIN1	mRNA_set2	NA	NA	miRDB
PPP4R2	mRNA_set2	NA	NA	miRDB
PDE10A	mRNA_set2	NA	NA	miRDB
PLPP3	mRNA_set2	NA	NA	miRDB
HAPLN1	mRNA_set2	NA	NA	miRDB
CLK2	mRNA_set2	NA	NA	miRDB
TNRC6B	mRNA_set2	NA	NA	miRDB
SOX11	mRNA_set2	NA	NA	miRDB
CA10	mRNA_set2	NA	NA	miRDB
SCAF11	mRNA_set2	NA	NA	miRDB
1-Mar	mRNA_set2	NA	NA	miRDB
ANGEL2	mRNA_set2	NA	NA	miRDB
MMP10	mRNA_set2	NA	NA	miRDB
S1PR1	mRNA_set2	NA	NA	miRDB
CDH20	mRNA_set2	NA	NA	miRDB
BMP3	mRNA_set2	NA	NA	miRDB

NFAT5	mRNA_set2	NA	NA	miRDB
NCKIPSD	mRNA_set2	NA	NA	miRDB
MMP15	mRNA_set2	NA	NA	miRDB
NHS	mRNA_set2	NA	NA	miRDB
KCTD16	mRNA_set2	NA	NA	miRDB
CDC14A	mRNA_set2	NA	NA	miRDB
SRSF11	mRNA_set2	NA	NA	miRDB
HBS1L	mRNA_set2	NA	NA	miRDB
RANGAP1	mRNA_set2	NA	NA	miRDB
NR2C2AP	mRNA_set2	NA	NA	miRDB
TMEM63B	mRNA_set2	NA	NA	miRDB
SECISBP2L	mRNA_set2	NA	NA	miRDB
CYB5R4	mRNA_set2	NA	NA	miRDB
PITPNM2	mRNA_set2	NA	NA	miRDB
A4GNT	mRNA_set2	NA	NA	miRDB
TMEM170A	mRNA_set2	NA	NA	miRDB
RTN4IP1	mRNA_set2	NA	NA	miRDB
SERP1	mRNA_set2	NA	NA	miRDB
HNRNPU	mRNA_set2	NA	NA	miRDB
RNF145	mRNA_set2	NA	NA	miRDB
MECOM	mRNA_set2	NA	NA	miRDB
WWC1	mRNA_set2	NA	NA	miRDB
EBPL	mRNA_set2	NA	NA	miRDB
THAP12	mRNA_set2	NA	NA	miRDB
TIMP3	mRNA_set2	NA	NA	miRDB
PBX1	mRNA_set2	NA	NA	miRDB
CREB5	mRNA_set2	NA	NA	miRDB
SAMSN1	mRNA_set2	NA	NA	miRDB

LASP1	mRNA_set2	NA	NA	miRDB
NFATC2	mRNA_set2	NA	NA	miRDB
GCH1	mRNA_set2	NA	NA	miRDB
METTL21A	mRNA_set2	NA	NA	miRDB
PTPRG	mRNA_set2	NA	NA	miRDB
SMARCC1	mRNA_set2	NA	NA	miRDB
RABGAP1L	mRNA_set2	NA	NA	miRDB
ASXL3	mRNA_set2	NA	NA	miRDB
SEC61A1	mRNA_set2	NA	NA	miRDB
LRCH1	mRNA_set2	NA	NA	miRDB
PPIB	mRNA_set2	NA	NA	miRDB
PABPC1L2B	mRNA_set2	NA	NA	miRDB
RIT2	mRNA_set2	NA	NA	miRDB
VPS37D	mRNA_set2	NA	NA	miRDB
KCNQ3	mRNA_set2	NA	NA	miRDB
SPTLC1	mRNA_set2	NA	NA	miRDB
ACY1	mRNA_set2	NA	NA	miRDB
TLE4	mRNA_set2	NA	NA	miRDB
VEZF1	mRNA_set2	NA	NA	miRDB
GLMN	mRNA_set2	NA	NA	miRDB
PABPC5	mRNA_set2	NA	NA	miRDB
COBL	mRNA_set2	NA	NA	miRDB
VWA3B	mRNA_set2	NA	NA	miRDB
LARP4B	mRNA_set2	NA	NA	miRDB
CNOT6L	mRNA_set2	NA	NA	miRDB
TMEM108	mRNA_set2	NA	NA	miRDB
TRDMT1	mRNA_set2	NA	NA	miRDB
PDE3A	mRNA_set2	NA	NA	miRDB

KLF5	mRNA_set2	NA	NA	miRDB
USP37	mRNA_set2	NA	NA	miRDB
SRSF6	mRNA_set2	NA	NA	miRDB
TBC1D32	mRNA_set2	NA	NA	miRDB
CMPK1	mRNA_set2	NA	NA	miRDB
KBTBD3	mRNA_set2	NA	NA	miRDB
LIN28B	mRNA_set2	NA	NA	miRDB
MAP3K21	mRNA_set2	NA	NA	miRDB
LONRF1	mRNA_set2	NA	NA	miRDB
DYNC2LI1	mRNA_set2	NA	NA	miRDB
MTMR4	mRNA_set2	NA	NA	miRDB
KMT2C	mRNA_set2	NA	NA	miRDB
GOLGA1	mRNA_set2	NA	NA	miRDB
CIPC	mRNA_set2	NA	NA	miRDB
FAM133B	mRNA_set2	NA	NA	miRDB
IKZF2	mRNA_set2	NA	NA	miRDB
NGFR	mRNA_set2	NA	NA	miRDB
RGS8	mRNA_set2	NA	NA	miRDB
SNX10	mRNA_set2	NA	NA	miRDB
PIK3CA	mRNA_set2	NA	NA	miRDB
PSEN1	mRNA_set2	NA	NA	miRDB
OAF	mRNA_set2	NA	NA	miRDB
GALNT3	mRNA_set2	NA	NA	miRDB
PLCH1	mRNA_set2	NA	NA	miRDB
NDUFS4	mRNA_set2	NA	NA	miRDB
SORL1	mRNA_set2	NA	NA	miRDB
MOCS3	mRNA_set2	NA	NA	miRDB
HIC1	mRNA_set2	NA	NA	miRDB

PKIA	mRNA_set2	NA	NA	miRDB
SUCO	mRNA_set2	NA	NA	miRDB
SOGA1	mRNA_set2	NA	NA	miRDB
SMOC2	mRNA_set2	NA	NA	miRDB
ARL4C	mRNA_set2	NA	NA	miRDB
TXLNG	mRNA_set2	NA	NA	miRDB
EGFR	mRNA_set2	NA	NA	miRDB
FRS3	mRNA_set2	NA	NA	miRDB
TMEM189-UBE2V1	mRNA_set2	NA	NA	miRDB
STAC	mRNA_set2	NA	NA	miRDB
SLC22A23	mRNA_set2	NA	NA	miRDB
LCOR	mRNA_set2	NA	NA	miRDB
RELN	mRNA_set2	NA	NA	miRDB
KLHL29	mRNA_set2	NA	NA	miRDB
COLEC10	mRNA_set2	NA	NA	miRDB
PTGDR	mRNA_set2	NA	NA	miRDB
ANK1	mRNA_set2	NA	NA	miRDB
PAX9	mRNA_set2	NA	NA	miRDB
ZMYM4	mRNA_set2	NA	NA	miRDB
CDK5R1	mRNA_set2	NA	NA	miRDB
ARHGEF12	mRNA_set2	NA	NA	miRDB
SYNJ1	mRNA_set2	NA	NA	miRDB
EPN2	mRNA_set2	NA	NA	miRDB
TXNIP	mRNA_set2	NA	NA	miRDB
ATP4B	mRNA_set2	NA	NA	miRDB
EYA3	mRNA_set2	NA	NA	miRDB
NPTX1	mRNA_set2	NA	NA	miRDB
MTCL1	mRNA_set2	NA	NA	miRDB

TANC1	mRNA_set2	NA	NA	miRDB
YTHDC2	mRNA_set2	NA	NA	miRDB
PRKCZ	mRNA_set2	NA	NA	miRDB
MAP2K1	mRNA_set2	NA	NA	miRDB
SNN	mRNA_set2	NA	NA	miRDB
RC3H1	mRNA_set2	NA	NA	miRDB
PIGA	mRNA_set2	NA	NA	miRDB
SPTY2D1	mRNA_set2	NA	NA	miRDB
ADGRF5	mRNA_set2	NA	NA	miRDB
AP4E1	mRNA_set2	NA	NA	miRDB
ZNF804A	mRNA_set2	NA	NA	miRDB
GPM6A	mRNA_set2	NA	NA	miRDB
MAB21L1	mRNA_set2	NA	NA	miRDB
ZBTB41	mRNA_set2	NA	NA	miRDB
SLC25A53	mRNA_set2	NA	NA	miRDB
IP6K2	mRNA_set2	NA	NA	miRDB
CCSAP	mRNA_set2	NA	NA	miRDB
SYT1	mRNA_set2	NA	NA	miRDB
ZFP36L1	mRNA_set2	NA	NA	miRDB
TFE3	mRNA_set2	NA	NA	miRDB
ADPGK	mRNA_set2	NA	NA	miRDB
NCL	mRNA_set2	NA	NA	miRDB
MGAT4A	mRNA_set2	NA	NA	miRDB
WBP1L	mRNA_set2	NA	NA	miRDB
ETS1	mRNA_set2	NA	NA	miRDB
CCDC32	mRNA_set2	NA	NA	miRDB
TNPO2	mRNA_set2	NA	NA	miRDB
RGS7	mRNA_set2	NA	NA	miRDB

TSPAN4	mRNA_set2	NA	NA	miRDB
S100A7A	mRNA_set2	NA	NA	miRDB
GCLC	mRNA_set2	NA	NA	miRDB
NR2C1	mRNA_set2	NA	NA	miRDB
SLAMF9	mRNA_set2	NA	NA	miRDB
ATXN7	mRNA_set2	NA	NA	miRDB
11-Sep	mRNA_set2	NA	NA	miRDB
USP15	mRNA_set2	NA	NA	miRDB
PTHLH	mRNA_set2	NA	NA	miRDB
CERS2	mRNA_set2	NA	NA	miRDB
ANKRD27	mRNA_set2	NA	NA	miRDB
CROT	mRNA_set2	NA	NA	miRDB
STAT3	mRNA_set2	NA	NA	miRDB
PLEKHF2	mRNA_set2	NA	NA	miRDB
NPAS2	mRNA_set2	NA	NA	miRDB
JAM3	mRNA_set2	NA	NA	miRDB
TET2	mRNA_set2	NA	NA	miRDB
EPC1	mRNA_set2	NA	NA	miRDB
ARIH1	mRNA_set2	NA	NA	miRDB
ATF1	mRNA_set2	NA	NA	miRDB
ATP5F1C	mRNA_set2	NA	NA	miRDB
PLCL1	mRNA_set2	NA	NA	miRDB
CORO2A	mRNA_set2	NA	NA	miRDB
RSBN1	mRNA_set2	NA	NA	miRDB
PIP4P2	mRNA_set2	NA	NA	miRDB
NAALADL2	mRNA_set2	NA	NA	miRDB
USP8	mRNA_set2	NA	NA	miRDB
LILRA1	mRNA_set2	NA	NA	miRDB

L3HYPDH	mRNA_set2	NA	NA	miRDB
SETD5	mRNA_set2	NA	NA	miRDB
CCM2	mRNA_set2	NA	NA	miRDB
COMMD3-BMI1	mRNA_set2	NA	NA	miRDB
ATP2B1	mRNA_set2	NA	NA	miRDB
USF3	mRNA_set2	NA	NA	miRDB
OTULIN	mRNA_set2	NA	NA	miRDB
FZD4	mRNA_set2	NA	NA	miRDB
HSD17B12	mRNA_set2	NA	NA	miRDB
HOXC6	mRNA_set2	NA	NA	miRDB
CAPN15	mRNA_set2	NA	NA	miRDB
TRIM50	mRNA_set2	NA	NA	miRDB
GATA6	mRNA_set2	NA	NA	miRDB
RARA	mRNA_set2	NA	NA	miRDB
FRMD6	mRNA_set2	NA	NA	miRDB
ZNF329	mRNA_set2	NA	NA	miRDB
SIX1	mRNA_set2	NA	NA	miRDB
AQP11	mRNA_set2	NA	NA	miRDB
PSPC1	mRNA_set2	NA	NA	miRDB
NEK6	mRNA_set2	NA	NA	miRDB
PDPK1	mRNA_set2	NA	NA	miRDB
COLGALT2	mRNA_set2	NA	NA	miRDB
SLC9B1	mRNA_set2	NA	NA	miRDB
PLXND1	mRNA_set2	NA	NA	miRDB
PATZ1	mRNA_set2	NA	NA	miRDB
PLPPR1	mRNA_set2	NA	NA	miRDB
MED14	mRNA_set2	NA	NA	miRDB
RSBN1L	mRNA_set2	NA	NA	miRDB

ZNF268	mRNA_set2	NA	NA	miRDB
RSPO3	mRNA_set2	NA	NA	miRDB
NRBF2	mRNA_set2	NA	NA	miRDB
LITAF	mRNA_set2	NA	NA	miRDB
TRAPPC8	mRNA_set2	NA	NA	miRDB
RPGRIP1L	mRNA_set2	NA	NA	miRDB
NCALD	mRNA_set2	NA	NA	miRDB
PF4V1	mRNA_set2	NA	NA	miRDB
EPB41	mRNA_set2	NA	NA	miRDB
KPNB1	mRNA_set2	NA	NA	miRDB
MKLN1	mRNA_set2	NA	NA	miRDB
TCIM	mRNA_set2	NA	NA	miRDB
CDH11	mRNA_set2	NA	NA	miRDB
MAFB	mRNA_set2	NA	NA	miRDB
CNTNAP3B	mRNA_set2	NA	NA	miRDB
PRICKLE2	mRNA_set2	NA	NA	miRDB
RICTOR	mRNA_set2	NA	NA	miRDB
ZBTB18	mRNA_set2	NA	NA	miRDB
CBLL1	mRNA_set2	NA	NA	miRDB
EPS15	mRNA_set2	NA	NA	miRDB
TBL1XR1	mRNA_set2	NA	NA	miRDB
CELSR1	mRNA_set2	NA	NA	miRDB
FAM234A	mRNA_set2	NA	NA	miRDB
FMR1	mRNA_set2	NA	NA	miRDB
C18orf25	mRNA_set2	NA	NA	miRDB
UBAP2L	mRNA_set2	NA	NA	miRDB
GPATCH8	mRNA_set2	NA	NA	miRDB
HACE1	mRNA_set2	NA	NA	miRDB

PDE7A	mRNA_set2	NA	NA	miRDB
PEAK1	mRNA_set2	NA	NA	miRDB
UBE4A	mRNA_set2	NA	NA	miRDB
MTX1	mRNA_set2	NA	NA	miRDB
PHIP	mRNA_set2	NA	NA	miRDB
MAX	mRNA_set2	NA	NA	miRDB
STMN2	mRNA_set2	NA	NA	miRDB
DRD1	mRNA_set2	NA	NA	miRDB
INSM1	mRNA_set2	NA	NA	miRDB
VAMP4	mRNA_set2	NA	NA	miRDB
PTPN1	mRNA_set2	NA	NA	miRDB
SLC29A3	mRNA_set2	NA	NA	miRDB
YWHAZ	mRNA_set2	NA	NA	miRDB
TRIM2	mRNA_set2	NA	NA	miRDB
BSCL2	mRNA_set2	NA	NA	miRDB
ARCN1	mRNA_set2	NA	NA	miRDB
FAM126A	mRNA_set2	NA	NA	miRDB
ANXA2	mRNA_set2	NA	NA	miRDB
THRB	mRNA_set2	NA	NA	miRDB
ADGRA3	mRNA_set2	NA	NA	miRDB
ZNF547	mRNA_set2	NA	NA	miRDB
KANK4	mRNA_set2	NA	NA	miRDB
JOSD1	mRNA_set2	NA	NA	miRDB
MFSD2A	mRNA_set2	NA	NA	miRDB
OGN	mRNA_set2	NA	NA	miRDB
CNGB1	mRNA_set2	NA	NA	miRDB
MED23	mRNA_set2	NA	NA	miRDB
PRB3	mRNA_set2	NA	NA	miRDB

NDUFA5	mRNA_set2	NA	NA	miRDB
MMAA	mRNA_set2	NA	NA	miRDB
ZNF215	mRNA_set2	NA	NA	miRDB
CDK6	mRNA_set2	NA	NA	miRDB
SRRM1	mRNA_set2	NA	NA	miRDB
KLHL7	mRNA_set2	NA	NA	miRDB
STEAP2	mRNA_set2	NA	NA	miRDB
KCTD9	mRNA_set2	NA	NA	miRDB
STOX2	mRNA_set2	NA	NA	miRDB
POM121	mRNA_set2	NA	NA	miRDB
MAP3K8	mRNA_set2	NA	NA	miRDB
NR3C2	mRNA_set2	NA	NA	miRDB
ECSCR	mRNA_set2	NA	NA	miRDB
DOCK5	mRNA_set2	NA	NA	miRDB
ZNF80	mRNA_set2	NA	NA	miRDB
SLC24A1	mRNA_set2	NA	NA	miRDB
ZMAT3	mRNA_set2	NA	NA	miRDB
PRKG1	mRNA_set2	NA	NA	miRDB
CDK18	mRNA_set2	NA	NA	miRDB
INPP1	mRNA_set2	NA	NA	miRDB
BEST1	mRNA_set2	NA	NA	miRDB
GNG12	mRNA_set2	NA	NA	miRDB
ABL2	mRNA_set2	NA	NA	miRDB
ASAH1	mRNA_set2	NA	NA	miRDB
TMA7	mRNA_set2	NA	NA	miRDB
POGLUT1	mRNA_set2	NA	NA	miRDB
TOR2A	mRNA_set2	NA	NA	miRDB
ITGA8	mRNA_set2	NA	NA	miRDB

OTX2	mRNA_set2	NA	NA	miRDB
GATA3	mRNA_set2	NA	NA	miRDB
ARX	mRNA_set2	NA	NA	miRDB
RUFY3	mRNA_set2	NA	NA	miRDB
NRARP	mRNA_set2	NA	NA	miRDB
PDHX	mRNA_set2	NA	NA	miRDB
RBPMS2	mRNA_set2	NA	NA	miRDB
TOM1L1	mRNA_set2	NA	NA	miRDB
CACNA2D3	mRNA_set2	NA	NA	miRDB
WSB1	mRNA_set2	NA	NA	miRDB
MAPK14	mRNA_set2	NA	NA	miRDB
GLRA2	mRNA_set2	NA	NA	miRDB
GFPT2	mRNA_set2	NA	NA	miRDB
CCNJ	mRNA_set2	NA	NA	miRDB
MAP3K4	mRNA_set2	NA	NA	miRDB
DKK2	mRNA_set2	NA	NA	miRDB
RASSF3	mRNA_set2	NA	NA	miRDB
ZBTB20	mRNA_set2	NA	NA	miRDB
EN2	mRNA_set2	NA	NA	miRDB
RNGTT	mRNA_set2	NA	NA	miRDB
NEURL1B	mRNA_set2	NA	NA	miRDB
MS4A7	mRNA_set2	NA	NA	miRDB
GRB2	mRNA_set2	NA	NA	miRDB
ABCD3	mRNA_set2	NA	NA	miRDB
SLC2A1	mRNA_set2	NA	NA	miRDB
UCP3	mRNA_set2	NA	NA	miRDB
TEK	mRNA_set2	NA	NA	miRDB
TGFB2	mRNA_set2	NA	NA	miRDB

ADAMTS15	mRNA_set2	NA	NA	miRDB
CSF1	mRNA_set2	NA	NA	miRDB
DLG2	mRNA_set2	NA	NA	miRDB
ELAVL2	mRNA_set2	NA	NA	miRDB
CASZ1	mRNA_set2	NA	NA	miRDB
FAM161A	mRNA_set2	NA	NA	miRDB
ARRDC3	mRNA_set2	NA	NA	miRDB
GAP43	mRNA_set2	NA	NA	miRDB
ARF4	mRNA_set2	NA	NA	miRDB
MIER1	mRNA_set2	NA	NA	miRDB
VMP1	mRNA_set2	NA	NA	miRDB
UBE4B	mRNA_set2	NA	NA	miRDB
RNF219	mRNA_set2	NA	NA	miRDB
CNTN4	mRNA_set2	NA	NA	miRDB
PHACTR2	mRNA_set2	NA	NA	miRDB
FAM72C	mRNA_set2	NA	NA	miRDB
CDKL5	mRNA_set2	NA	NA	miRDB
AZIN1	mRNA_set2	NA	NA	miRDB
NOL4L	mRNA_set2	NA	NA	miRDB
ADAM12	mRNA_set2	NA	NA	miRDB
MPP7	mRNA_set2	NA	NA	miRDB
SEMA6D	mRNA_set2	NA	NA	miRDB
NDRG3	mRNA_set2	NA	NA	miRDB
MIPOL1	mRNA_set2	NA	NA	miRDB
FAM72D	mRNA_set2	NA	NA	miRDB
NEXMIF	mRNA_set2	NA	NA	miRDB
FOSB	mRNA_set2	NA	NA	miRDB
IGF1	mRNA_set2	NA	NA	miRDB

PHF6	mRNA_set2	NA	NA	miRDB
OSBPL7	mRNA_set2	NA	NA	miRDB
ZNF24	mRNA_set2	NA	NA	miRDB
RAB5A	mRNA_set2	NA	NA	miRDB
FAM72A	mRNA_set2	NA	NA	miRDB
KMT2E	mRNA_set2	NA	NA	miRDB
FAM72B	mRNA_set2	NA	NA	miRDB
GPR158	mRNA_set2	NA	NA	miRDB
DLG1	mRNA_set2	NA	NA	miRDB
HMGN1	mRNA_set2	NA	NA	miRDB
HSCB	mRNA_set2	NA	NA	miRDB
ITPRID1	mRNA_set2	NA	NA	miRDB
GALNS	mRNA_set2	NA	NA	miRDB
RAPGEF3	mRNA_set2	NA	NA	miRDB
DLL1	mRNA_set2	NA	NA	miRDB
MARK3	mRNA_set2	NA	NA	miRDB
CLIC5	mRNA_set2	NA	NA	miRDB
CCL28	mRNA_set2	NA	NA	miRDB
SPINK8	mRNA_set2	NA	NA	miRDB
URI1	mRNA_set2	NA	NA	miRDB
NUP160	mRNA_set2	NA	NA	miRDB
DYNLT1	mRNA_set2	NA	NA	miRDB
STRADB	mRNA_set2	NA	NA	miRDB
HPS5	mRNA_set2	NA	NA	miRDB
SLC2A12	mRNA_set2	NA	NA	miRDB
STK17B	mRNA_set2	NA	NA	miRDB
CPNE3	mRNA_set2	NA	NA	miRDB
C15orf41	mRNA_set2	NA	NA	miRDB

CISD1	mRNA_set2	NA	NA	miRDB
KIAA1143	mRNA_set2	NA	NA	miRDB
ATRX	mRNA_set2	NA	NA	miRDB
SGIP1	mRNA_set2	NA	NA	miRDB
CD44	mRNA_set2	NA	NA	miRDB
TBC1D23	mRNA_set2	NA	NA	miRDB
NHLRC2	mRNA_set2	NA	NA	miRDB
PREX1	mRNA_set2	NA	NA	miRDB
ACER3	mRNA_set2	NA	NA	miRDB
TNFAIP3	mRNA_set2	NA	NA	miRDB
SLC30A7	mRNA_set2	NA	NA	miRDB
TGFBR3	mRNA_set2	NA	NA	miRDB
LOX	mRNA_set2	NA	NA	miRDB
PDE3B	mRNA_set2	NA	NA	miRDB
GPR6	mRNA_set2	NA	NA	miRDB
TMEM91	mRNA_set2	NA	NA	miRDB
NKAIN1	mRNA_set2	NA	NA	miRDB
NAV2	mRNA_set2	NA	NA	miRDB
NHLH2	mRNA_set2	NA	NA	miRDB
NRP2	mRNA_set2	NA	NA	miRDB
OGA	mRNA_set2	NA	NA	miRDB
RNF8	mRNA_set2	NA	NA	miRDB
ALDH5A1	mRNA_set2	NA	NA	miRDB
ATP11C	mRNA_set2	NA	NA	miRDB
SHC4	mRNA_set2	NA	NA	miRDB
NCAM1	mRNA_set2	NA	NA	miRDB
MDM4	mRNA_set2	NA	NA	miRDB
PRKX	mRNA_set2	NA	NA	miRDB

TERB2	mRNA_set2	NA	NA	miRDB
ERICH3	mRNA_set2	NA	NA	miRDB
MIER2	mRNA_set2	NA	NA	miRDB
NSD1	mRNA_set2	NA	NA	miRDB
NR2F2	mRNA_set2	NA	NA	miRDB
CMKLR1	mRNA_set2	NA	NA	miRDB
ABCB9	mRNA_set2	NA	NA	miRDB
CDC42EP3	mRNA_set2	NA	NA	miRDB
EFNB2	mRNA_set2	NA	NA	miRDB
CDIP1	mRNA_set2	NA	NA	miRDB
NXF1	mRNA_set2	NA	NA	miRDB
ERG	mRNA_set2	NA	NA	miRDB
INA	mRNA_set2	NA	NA	miRDB
NCOA5	mRNA_set2	NA	NA	miRDB
CALD1	mRNA_set2	NA	NA	miRDB
ABHD17B	mRNA_set2	NA	NA	miRDB
DEPDC4	mRNA_set2	NA	NA	miRDB
GEM	mRNA_set2	NA	NA	miRDB
MLLT10	mRNA_set2	NA	NA	miRDB
ADAM23	mRNA_set2	NA	NA	miRDB
MDFIC	mRNA_set2	NA	NA	miRDB
TMEM54	mRNA_set2	NA	NA	miRDB
AGO1	mRNA_set2	NA	NA	miRDB
ITGA5	mRNA_set2	NA	NA	miRDB
ALCAM	mRNA_set2	NA	NA	miRDB
TOMM70	mRNA_set2	NA	NA	miRDB
CCDC141	mRNA_set2	NA	NA	miRDB
LTBP1	mRNA_set2	NA	NA	miRDB

USP31	mRNA_set2	NA	NA	miRDB
CUL5	mRNA_set2	NA	NA	miRDB
RGMA	mRNA_set2	NA	NA	miRDB
MTMR9	mRNA_set2	NA	NA	miRDB
NCOA1	mRNA_set2	NA	NA	miRDB
ELAVL4	mRNA_set2	NA	NA	miRDB
MTMR10	mRNA_set2	NA	NA	miRDB
FEZ2	mRNA_set2	NA	NA	miRDB
ARFIP1	mRNA_set2	NA	NA	miRDB
TBC1D15	mRNA_set2	NA	NA	miRDB
ARHGAP20	mRNA_set2	NA	NA	miRDB
NINJ1	mRNA_set2	NA	NA	miRDB
PRKCE	mRNA_set2	NA	NA	miRDB
SMARCB1	mRNA_set2	NA	NA	miRDB
TOX3	mRNA_set2	NA	NA	miRDB
KMT5B	mRNA_set2	NA	NA	miRDB
WDR1	mRNA_set2	NA	NA	miRDB
MEA1	mRNA_set2	NA	NA	miRDB
SLC7A2	mRNA_set2	NA	NA	miRDB
ZBTB6	mRNA_set2	NA	NA	miRDB
FBXL14	mRNA_set2	NA	NA	miRDB
MBLAC2	mRNA_set2	NA	NA	miRDB
CCL2	mRNA_set2	NA	NA	miRDB
THBS1	mRNA_set2	NA	NA	miRDB
CITED2	mRNA_set2	NA	NA	miRDB
ASH2L	mRNA_set2	NA	NA	miRDB
NETO2	mRNA_set2	NA	NA	miRDB
SATB1	mRNA_set2	NA	NA	miRDB

SLC38A3	mRNA_set2	NA	NA	miRDB
MINDY2	mRNA_set2	NA	NA	miRDB
CHSY1	mRNA_set2	NA	NA	miRDB
DFFA	mRNA_set2	NA	NA	miRDB
ERN2	mRNA_set2	NA	NA	miRDB
UBE2R2	mRNA_set2	NA	NA	miRDB
ATP7A	mRNA_set2	NA	NA	miRDB
CDK5RAP2	mRNA_set2	NA	NA	miRDB
CLSTN2	mRNA_set2	NA	NA	miRDB
NALCN	mRNA_set2	NA	NA	miRDB
COPS8	mRNA_set2	NA	NA	miRDB
TRIM38	mRNA_set2	NA	NA	miRDB
PRB1	mRNA_set2	NA	NA	miRDB
CYP7A1	mRNA_set2	NA	NA	miRDB
PDE8A	mRNA_set2	NA	NA	miRDB
RNF217	mRNA_set2	NA	NA	miRDB
GPC5	mRNA_set2	NA	NA	miRDB
SEC24D	mRNA_set2	NA	NA	miRDB
SIMC1	mRNA_set2	NA	NA	miRDB
SOCS6	mRNA_set2	NA	NA	miRDB
NGF	mRNA_set2	NA	NA	miRDB
AHR	mRNA_set2	NA	NA	miRDB
ALKBH1	mRNA_set2	NA	NA	miRDB
PAFAH1B1	mRNA_set2	NA	NA	miRDB
C21orf91	mRNA_set2	NA	NA	miRDB
PQLC3	mRNA_set2	NA	NA	miRDB
CAPS2	mRNA_set2	NA	NA	miRDB
CADPS	mRNA_set2	NA	NA	miRDB

GUF1	mRNA_set2	NA	NA	miRDB
HEATR5A	mRNA_set2	NA	NA	miRDB
SH3BP4	mRNA_set2	NA	NA	miRDB
PLS1	mRNA_set2	NA	NA	miRDB
PPP1R8	mRNA_set2	NA	NA	miRDB
PPP2R2A	mRNA_set2	NA	NA	miRDB
AMPD3	mRNA_set2	NA	NA	miRDB
POU2F3	mRNA_set2	NA	NA	miRDB
TAF5L	mRNA_set2	NA	NA	miRDB
SS18L1	mRNA_set2	NA	NA	miRDB
SLC35F4	mRNA_set2	NA	NA	miRDB
SLIT2	mRNA_set2	NA	NA	miRDB
GLTP	mRNA_set2	NA	NA	miRDB
ARRDC4	mRNA_set2	NA	NA	miRDB
NECAB1	mRNA_set2	NA	NA	miRDB
UNC13C	mRNA_set2	NA	NA	miRDB
MIGA2	mRNA_set2	NA	NA	miRDB
CAMTA1	mRNA_set2	NA	NA	miRDB
PAIP2	mRNA_set2	NA	NA	miRDB
APOOL	mRNA_set2	NA	NA	miRDB
SYNRG	mRNA_set2	NA	NA	miRDB
PF4	mRNA_set2	NA	NA	miRDB
ATP1A2	mRNA_set2	NA	NA	miRDB
CCNC	mRNA_set2	NA	NA	miRDB
TAPT1	mRNA_set2	NA	NA	miRDB
KCTD14	mRNA_set2	NA	NA	miRDB
WIPF2	mRNA_set2	NA	NA	miRDB
SPATA13	mRNA_set2	NA	NA	miRDB

LIPT2	mRNA_set2	NA	NA	miRDB
CCNY	mRNA_set2	NA	NA	miRDB
FN1	mRNA_set2	NA	NA	miRDB
RPN2	mRNA_set2	NA	NA	miRDB
LIMK1	mRNA_set2	NA	NA	miRDB
SLCO5A1	mRNA_set2	NA	NA	miRDB
PCLO	mRNA_set2	NA	NA	miRDB
EPAS1	mRNA_set2	NA	NA	miRDB
SCN9A	mRNA_set2	NA	NA	miRDB
NOG	mRNA_set2	NA	NA	miRDB
MRAS	mRNA_set2	NA	NA	miRDB
MOSPD1	mRNA_set2	NA	NA	miRDB
MLLT6	mRNA_set2	NA	NA	miRDB
ITGA9	mRNA_set2	NA	NA	miRDB
CD72	mRNA_set2	NA	NA	miRDB
H2AFY	mRNA_set2	NA	NA	miRDB
SSR1	mRNA_set2	NA	NA	miRDB
USP48	mRNA_set2	NA	NA	miRDB
CANX	mRNA_set2	NA	NA	miRDB
NT5C3A	mRNA_set2	NA	NA	miRDB
NAT14	mRNA_set2	NA	NA	miRDB
LRRC41	mRNA_set2	NA	NA	miRDB
FOXF1	mRNA_set2	NA	NA	miRDB
BPNT1	mRNA_set2	NA	NA	miRDB
HIPK3	mRNA_set2	NA	NA	miRDB
CAPRIN1	mRNA_set2	NA	NA	miRDB
AKAP11	mRNA_set2	NA	NA	miRDB
CAP1	mRNA_set2	NA	NA	miRDB

ACTB	mRNA_set2	NA	NA	miRDB
BHLHE22	mRNA_set2	NA	NA	miRDB
MAP4K2	mRNA_set2	NA	NA	miRDB
TBCK	mRNA_set2	NA	NA	miRDB
E2F5	mRNA_set2	NA	NA	miRDB
SOWAHC	mRNA_set2	NA	NA	miRDB
CPEB1	mRNA_set2	NA	NA	miRDB
INMT	mRNA_set2	NA	NA	miRDB
NBEA	mRNA_set2	NA	NA	miRDB
RNF138	mRNA_set2	NA	NA	miRDB
ZNF280D	mRNA_set2	NA	NA	miRDB
SNAI2	mRNA_set2	NA	NA	miRDB
SLC35G1	mRNA_set2	NA	NA	miRDB
MAL2	mRNA_set2	NA	NA	miRDB
FAIM	mRNA_set2	NA	NA	miRDB
ZEB2	mRNA_set2	NA	NA	miRDB
BACH2	mRNA_set2	NA	NA	miRDB
GRK6	mRNA_set2	NA	NA	miRDB
AP1S1	mRNA_set2	NA	NA	miRDB
NET1	mRNA_set2	NA	NA	miRDB
RSPRY1	mRNA_set2	NA	NA	miRDB
ZBTB8A	mRNA_set2	NA	NA	miRDB
SUPT4H1	mRNA_set2	NA	NA	miRDB
TFB1M	mRNA_set2	NA	NA	miRDB
EGR3	mRNA_set2	NA	NA	miRDB
CTDSP1	mRNA_set2	NA	NA	miRDB
PRB2	mRNA_set2	NA	NA	miRDB
RNFT2	mRNA_set2	NA	NA	miRDB

ABHD2	mRNA_set2	NA	NA	miRDB
P3H1	mRNA_set2	NA	NA	miRDB
ME2	mRNA_set2	NA	NA	miRDB
CACNA1G	mRNA_set2	NA	NA	miRDB
EFEMP1	mRNA_set2	NA	NA	miRDB
ANKRD39	mRNA_set2	NA	NA	miRDB
ASB8	mRNA_set2	NA	NA	miRDB
KAZN	mRNA_set2	NA	NA	miRDB
LRRTM3	mRNA_set2	NA	NA	miRDB
SENP2	mRNA_set2	NA	NA	miRDB
CHMP4C	mRNA_set2	NA	NA	miRDB
SCAI	mRNA_set2	NA	NA	miRDB
UBTD2	mRNA_set2	NA	NA	miRDB
MNX1	mRNA_set2	NA	NA	miRDB
SLC36A4	mRNA_set2	NA	NA	miRDB
PHYKPL	mRNA_set2	NA	NA	miRDB
GABPA	mRNA_set2	NA	NA	miRDB
JADE3	mRNA_set2	NA	NA	miRDB
PPP2CB	mRNA_set2	NA	NA	miRDB
EMC7	mRNA_set2	NA	NA	miRDB
SERPINB4	mRNA_set2	NA	NA	miRDB
NCOA2	mRNA_set2	NA	NA	miRDB
RYBP	mRNA_set2	NA	NA	miRDB
FBLN5	mRNA_set2	NA	NA	miRDB
SRL	mRNA_set2	NA	NA	miRDB
VAV3	mRNA_set2	NA	NA	miRDB
AFAP1	mRNA_set2	NA	NA	miRDB
KPNA3	mRNA_set2	NA	NA	miRDB

ARHGEF7	mRNA_set2	NA	NA	miRDB
WDCP	mRNA_set2	NA	NA	miRDB
AGRN	mRNA_set2	NA	NA	miRDB
C11orf58	mRNA_set2	NA	NA	miRDB
BEND4	mRNA_set2	NA	NA	miRDB
SOWAHA	mRNA_set2	NA	NA	miRDB
ZNF708	mRNA_set2	NA	NA	miRDB
AKIRIN2	mRNA_set2	NA	NA	miRDB
SLC24A4	mRNA_set2	NA	NA	miRDB
SLC13A3	mRNA_set2	NA	NA	miRDB
ST14	mRNA_set2	NA	NA	miRDB
INSR	mRNA_set2	NA	NA	miRDB
FOXP4	mRNA_set2	NA	NA	miRDB
PDK4	mRNA_set2	NA	NA	miRDB
BTG1	mRNA_set2	NA	NA	miRDB
NCOA7	mRNA_set2	NA	NA	miRDB
SEC62	mRNA_set2	NA	NA	miRDB
FCRL2	mRNA_set2	NA	NA	miRDB
PNISR	mRNA_set2	NA	NA	miRDB
FAM78A	mRNA_set2	NA	NA	miRDB
ANKS1A	mRNA_set2	NA	NA	miRDB
WDR37	mRNA_set2	NA	NA	miRDB
GATA2	mRNA_set2	NA	NA	miRDB
PTCH1	mRNA_set2	NA	NA	miRDB
GATAD2A	mRNA_set2	NA	NA	miRDB
ZFP36	mRNA_set2	NA	NA	miRDB
SEH1L	mRNA_set2	NA	NA	miRDB
HSPH1	mRNA_set2	NA	NA	miRDB

SLC9A4	mRNA_set2	NA	NA	miRDB
CCNT2	mRNA_set2	NA	NA	miRDB
KCNJ3	mRNA_set2	NA	NA	miRDB
PRLR	mRNA_set2	NA	NA	miRDB
PAIP2B	mRNA_set2	NA	NA	miRDB
TRIL	mRNA_set2	NA	NA	miRDB
MRPS14	mRNA_set2	NA	NA	miRDB
SATB2	mRNA_set2	NA	NA	miRDB
FCRL1	mRNA_set2	NA	NA	miRDB
HSDL1	mRNA_set2	NA	NA	miRDB
CCN4	mRNA_set2	NA	NA	miRDB
MTMR14	mRNA_set2	NA	NA	miRDB
WNT10B	mRNA_set2	NA	NA	miRDB
SIX4	mRNA_set2	NA	NA	miRDB
CLTA	mRNA_set2	NA	NA	miRDB
ING2	mRNA_set2	NA	NA	miRDB
TGOLN2	mRNA_set2	NA	NA	miRDB
MNT	mRNA_set2	NA	NA	miRDB
CERS6	mRNA_set2	NA	NA	miRDB
ROCK1	mRNA_set2	NA	NA	miRDB
WDR20	mRNA_set2	NA	NA	miRDB
CAMSAP2	mRNA_set2	NA	NA	miRDB
MMP13	mRNA_set2	NA	NA	miRDB
IL15	mRNA_set2	NA	NA	miRDB
WDR61	mRNA_set2	NA	NA	miRDB
PDK3	mRNA_set2	NA	NA	miRDB
SLC25A36	mRNA_set2	NA	NA	miRDB
BCL11A	mRNA_set2	NA	NA	miRDB

ELF1	mRNA_set2	NA	NA	miRDB
RFWD3	mRNA_set2	NA	NA	miRDB
FNDC3B	mRNA_set2	NA	NA	miRDB
PFDN1	mRNA_set2	NA	NA	miRDB
FUBP1	mRNA_set2	NA	NA	miRDB
TBX3	mRNA_set2	NA	NA	miRDB
TBX18	mRNA_set2	NA	NA	miRDB
ACER2	mRNA_set2	NA	NA	miRDB
MYLK	mRNA_set2	NA	NA	miRDB
SOX9	mRNA_set2	NA	NA	miRDB
GIT1	mRNA_set2	NA	NA	miRDB
UBE2H	mRNA_set2	NA	NA	miRDB
FAM91A1	mRNA_set2	NA	NA	miRDB
AJUBA	mRNA_set2	NA	NA	miRDB
TTC7B	mRNA_set2	NA	NA	miRDB
NAB1	mRNA_set2	NA	NA	miRDB
USP38	mRNA_set2	NA	NA	miRDB
KCNH5	mRNA_set2	NA	NA	miRDB
SORCS1	mRNA_set2	NA	NA	miRDB
CNPY3	mRNA_set2	NA	NA	miRDB
CISD2	mRNA_set2	NA	NA	miRDB
RAD21	mRNA_set2	NA	NA	miRDB
CLCC1	mRNA_set2	NA	NA	miRDB
TMEM251	mRNA_set2	NA	NA	miRDB
SH3TC2	mRNA_set2	NA	NA	miRDB
LACTB2	mRNA_set2	NA	NA	miRDB
TMEM174	mRNA_set2	NA	NA	miRDB
APP	mRNA_set2	NA	NA	miRDB

CSN1S1	mRNA_set2	NA	NA	miRDB
SMC5	mRNA_set2	NA	NA	miRDB
SERPINB3	mRNA_set2	NA	NA	miRDB
FGF4	mRNA_set2	NA	NA	miRDB
USP12	mRNA_set2	NA	NA	miRDB
KNSTRN	mRNA_set2	NA	NA	miRDB
USP30	mRNA_set2	NA	NA	miRDB
ATP1B1	mRNA_set2	NA	NA	miRDB
PRKCI	mRNA_set2	NA	NA	miRDB
JADE1	mRNA_set2	NA	NA	miRDB
SENP7	mRNA_set2	NA	NA	miRDB
BMT2	mRNA_set2	NA	NA	miRDB
KIAA1549L	mRNA_set2	NA	NA	miRDB
HYAL2	mRNA_set2	NA	NA	miRDB
VPS72	mRNA_set2	NA	NA	miRDB
FERMT2	mRNA_set2	NA	NA	miRDB
RAB21	mRNA_set2	NA	NA	miRDB
SGMS1	mRNA_set2	NA	NA	miRDB
GOLPH3	mRNA_set2	NA	NA	miRDB
MAGI3	mRNA_set2	NA	NA	miRDB
XPO1	mRNA_set2	NA	NA	miRDB
GPT2	mRNA_set2	NA	NA	miRDB
COL19A1	mRNA_set2	NA	NA	miRDB
TET1	mRNA_set2	NA	NA	miRDB
CHD7	mRNA_set2	NA	NA	miRDB
MDN1	mRNA_set2	NA	NA	miRDB
TSC1	mRNA_set2	NA	NA	miRDB
PLEKHA5	mRNA_set2	NA	NA	miRDB

STK39	mRNA_set2	NA	NA	miRDB
SLFN5	mRNA_set2	NA	NA	miRDB
CCDC28B	mRNA_set2	NA	NA	miRDB
SRP19	mRNA_set2	NA	NA	miRDB
FOXO1	mRNA_set2	NA	NA	miRDB
CYP39A1	mRNA_set2	NA	NA	miRDB
HEG1	mRNA_set2	NA	NA	miRDB
RAB11FIP1	mRNA_set2	NA	NA	miRDB
LYSMD3	mRNA_set2	NA	NA	miRDB
KHSRP	mRNA_set2	NA	NA	miRDB
SLC25A16	mRNA_set2	NA	NA	miRDB
COL21A1	mRNA_set2	NA	NA	miRDB
CTU1	mRNA_set2	NA	NA	miRDB
RIPOR2	mRNA_set2	NA	NA	miRDB
KMT5A	mRNA_set2	NA	NA	miRDB
TRAF3IP3	mRNA_set2	NA	NA	miRDB
MSL1	mRNA_set2	NA	NA	miRDB
NDUFC2-KCTD14	mRNA_set2	NA	NA	miRDB
FOXO3	mRNA_set2	NA	NA	miRDB
SPRY2	mRNA_set2	NA	NA	miRDB
BTBD10	mRNA_set2	NA	NA	miRDB
TRAK2	mRNA_set2	NA	NA	miRDB
PRKAG2	mRNA_set2	NA	NA	miRDB
CHUK	mRNA_set2	NA	NA	miRDB
NOL4	mRNA_set2	NA	NA	miRDB
PGRMC2	mRNA_set2	NA	NA	miRDB
SLC16A6	mRNA_set2	NA	NA	miRDB
ZCCHC2	mRNA_set2	NA	NA	miRDB

MITF	mRNA_set2	NA	NA	miRDB
TRIM59	mRNA_set2	NA	NA	miRDB
KLF4	mRNA_set2	NA	NA	miRDB
CFL2	mRNA_set2	NA	NA	miRDB
SLC37A3	mRNA_set2	NA	NA	miRDB
SPRN	mRNA_set2	NA	NA	miRDB
DDX55	mRNA_set2	NA	NA	miRDB
SP2	mRNA_set2	NA	NA	miRDB
PICALM	mRNA_set2	NA	NA	miRDB
SEC63	mRNA_set2	NA	NA	miRDB
ZBTB4	mRNA_set2	NA	NA	miRDB
ARPC3	mRNA_set2	NA	NA	miRDB
MCTP1	mRNA_set2	NA	NA	miRDB
LAMP2	mRNA_set2	NA	NA	miRDB
PIRT	mRNA_set2	NA	NA	miRDB
MET	mRNA_set2	NA	NA	miRDB
ZNF326	mRNA_set2	NA	NA	miRDB
DGKH	mRNA_set2	NA	NA	miRDB
KAT6B	mRNA_set2	NA	NA	miRDB
RNF165	mRNA_set2	NA	NA	miRDB
CTBS	mRNA_set2	NA	NA	miRDB
PRR9	mRNA_set2	NA	NA	miRDB
TMEM178B	mRNA_set2	NA	NA	miRDB
INSL5	mRNA_set2	NA	NA	miRDB
KLHL36	mRNA_set2	NA	NA	miRDB
TUBB6	mRNA_set2	NA	NA	miRDB
PCDHGA11	mRNA_set2	NA	NA	miRDB
PLXDC2	mRNA_set2	NA	NA	miRDB

GABARAPL2	mRNA_set2	NA	NA	miRDB
ZDHHC21	mRNA_set2	NA	NA	miRDB
F13B	mRNA_set2	NA	NA	miRDB
SMNDC1	mRNA_set2	NA	NA	miRDB
OLFM3	mRNA_set2	NA	NA	miRDB
FAM8A1	mRNA_set2	NA	NA	miRDB
MED13	mRNA_set2	NA	NA	miRDB
HTR2A	mRNA_set2	NA	NA	miRDB
LAMA4	mRNA_set2	NA	NA	miRDB
PCM1	mRNA_set2	NA	NA	miRDB
TTC28	mRNA_set2	NA	NA	miRDB
REXO4	mRNA_set2	NA	NA	miRDB
DPP10	mRNA_set2	NA	NA	miRDB
BDH2	mRNA_set2	NA	NA	miRDB
KCTD13	mRNA_set2	NA	NA	miRDB
YWHAE	mRNA_set2	NA	NA	miRDB
INPPL1	mRNA_set2	NA	NA	miRDB
ZIC1	mRNA_set2	NA	NA	miRDB
PLAG1	mRNA_set2	NA	NA	miRDB
NUP210	mRNA_set2	NA	NA	miRDB
ADCY6	mRNA_set2	NA	NA	miRDB
CAMK1D	mRNA_set2	NA	NA	miRDB
RAPGEF2	mRNA_set2	NA	NA	miRDB
PHB	mRNA_set2	NA	NA	miRDB
KIAA0319L	mRNA_set2	NA	NA	miRDB
NEDD4	mRNA_set2	NA	NA	miRDB
RGL2	mRNA_set2	NA	NA	miRDB
UBR5	mRNA_set2	NA	NA	miRDB

UNC5D	mRNA_set2	NA	NA	miRDB
PSMA1	mRNA_set2	NA	NA	miRDB
ZNF426	mRNA_set2	NA	NA	miRDB
HDHD2	mRNA_set2	NA	NA	miRDB
FAM217B	mRNA_set2	NA	NA	miRDB
KCNK5	mRNA_set2	NA	NA	miRDB
PRMT8	mRNA_set2	NA	NA	miRDB
AHSA2P	mRNA_set2	NA	NA	miRDB
ARMC8	mRNA_set2	NA	NA	miRDB
ALG9	mRNA_set2	NA	NA	miRDB
DLL4	mRNA_set2	NA	NA	miRDB
FGF7	mRNA_set2	NA	NA	miRDB
CIITA	mRNA_set2	NA	NA	miRDB
NME7	mRNA_set2	NA	NA	miRDB
DUSP1	mRNA_set2	NA	NA	miRDB
IGFBP5	mRNA_set2	NA	NA	miRDB
ITPK1	mRNA_set2	NA	NA	miRDB
ATP2B4	mRNA_set2	NA	NA	miRDB
ADAM17	mRNA_set2	NA	NA	miRDB
ANXA4	mRNA_set2	NA	NA	miRDB
PDE1C	mRNA_set2	NA	NA	miRDB
ATP8A1	mRNA_set2	NA	NA	miRDB
PPP1R9B	mRNA_set2	NA	NA	miRDB
KRTAP2-3	mRNA_set2	NA	NA	miRDB
CCKBR	mRNA_set2	NA	NA	miRDB
OTUD4	mRNA_set2	NA	NA	miRDB
SLC35B3	mRNA_set2	NA	NA	miRDB
FLI1	mRNA_set2	NA	NA	miRDB

CCDC146	mRNA_set2	NA	NA	miRDB
MRO	mRNA_set2	NA	NA	miRDB
TMEM135	mRNA_set2	NA	NA	miRDB
SLC8A2	mRNA_set2	NA	NA	miRDB
INTS6	mRNA_set2	NA	NA	miRDB
MMRN1	mRNA_set2	NA	NA	miRDB
NUP50	mRNA_set2	NA	NA	miRDB
SEC23B	mRNA_set2	NA	NA	miRDB
RAD54B	mRNA_set2	NA	NA	miRDB
TRAT1	mRNA_set2	NA	NA	miRDB
SLC35D1	mRNA_set2	NA	NA	miRDB
VAT1	mRNA_set2	NA	NA	miRDB
IGSF10	mRNA_set2	NA	NA	miRDB
MOSMO	mRNA_set2	NA	NA	miRDB
CHL1	mRNA_set2	NA	NA	miRDB
NPNT	mRNA_set2	NA	NA	miRDB
SLC38A1	mRNA_set2	NA	NA	miRDB
TIPARP	mRNA_set2	NA	NA	miRDB
TMEM196	mRNA_set2	NA	NA	miRDB
CAB39	mRNA_set2	NA	NA	miRDB
CFHR4	mRNA_set2	NA	NA	miRDB
DMRTA1	mRNA_set2	NA	NA	miRDB
BBX	mRNA_set2	NA	NA	miRDB
NDUFS1	mRNA_set2	NA	NA	miRDB
TGFBR1	mRNA_set2	NA	NA	miRDB
CNOT7	mRNA_set2	NA	NA	miRDB
USP24	mRNA_set2	NA	NA	miRDB
FEM1B	mRNA_set2	NA	NA	miRDB

DACT1	mRNA_set2	NA	NA	miRDB
CD58	mRNA_set2	NA	NA	miRDB
CFHR3	mRNA_set2	NA	NA	miRDB
CNIH1	mRNA_set2	NA	NA	miRDB
RP2	mRNA_set2	NA	NA	miRDB
LIN54	mRNA_set2	NA	NA	miRDB
C5orf47	mRNA_set2	NA	NA	miRDB
AMD1	mRNA_set2	NA	NA	miRDB
CELF2	mRNA_set2	NA	NA	miRDB
INPP5J	mRNA_set2	NA	NA	miRDB
CEMIP	mRNA_set2	NA	NA	miRDB
KLHL31	mRNA_set2	NA	NA	miRDB
NOVA1	mRNA_set2	NA	NA	miRDB
PNPLA7	mRNA_set2	NA	NA	miRDB
TSPYL1	mRNA_set2	NA	NA	miRDB
CPPED1	mRNA_set2	NA	NA	miRDB
RAP2A	mRNA_set2	NA	NA	miRDB
SLC35A3	mRNA_set2	NA	NA	miRDB
CHKA	mRNA_set2	NA	NA	miRDB
DYNLL2	mRNA_set2	NA	NA	miRDB
STX16	mRNA_set2	NA	NA	miRDB
ZBTB39	mRNA_set2	NA	NA	miRDB
SH3GL3	mRNA_set2	NA	NA	miRDB
SOX7	mRNA_set2	NA	NA	miRDB
COPZ1	mRNA_set2	NA	NA	miRDB
DYRK1A	mRNA_set2	NA	NA	miRDB
MAP2K7	mRNA_set2	NA	NA	miRDB
ZNF608	mRNA_set2	NA	NA	miRDB

PELI2	mRNA_set2	NA	NA	miRDB
EPM2A	mRNA_set2	NA	NA	miRDB
ATXN7L1	mRNA_set2	NA	NA	miRDB
PRKAA1	mRNA_set2	NA	NA	miRDB
DYRK2	mRNA_set2	NA	NA	miRDB
MPL	mRNA_set2	NA	NA	miRDB
MAFG	mRNA_set2	NA	NA	miRDB
RUNX2	mRNA_set2	NA	NA	miRDB
GPRC5A	mRNA_set2	NA	NA	miRDB
SESN3	mRNA_set2	NA	NA	miRDB
ZNF821	mRNA_set2	NA	NA	miRDB
RTN4	mRNA_set2	NA	NA	miRDB
FUT3	mRNA_set2	NA	NA	miRDB
RBM27	mRNA_set2	NA	NA	miRDB
MSANTD2	mRNA_set2	NA	NA	miRDB
GPR85	mRNA_set2	NA	NA	miRDB
GIPC2	mRNA_set2	NA	NA	miRDB
SLC8A1	mRNA_set2	NA	NA	miRDB
EML3	mRNA_set2	NA	NA	miRDB
NEDD9	mRNA_set2	NA	NA	miRDB
ANP32E	mRNA_set2	NA	NA	miRDB
SUSD1	mRNA_set2	NA	NA	miRDB
CWC15	mRNA_set2	NA	NA	miRDB
RIMS4	mRNA_set2	NA	NA	miRDB
SLC6A11	mRNA_set2	NA	NA	miRDB
ZNF250	mRNA_set2	NA	NA	miRDB
AP1B1	mRNA_set2	NA	NA	miRDB
SEMA4D	mRNA_set2	NA	NA	miRDB

FAM104B	mRNA_set2	NA	NA	miRDB
SSUH2	mRNA_set2	NA	NA	miRDB
SYT9	mRNA_set2	NA	NA	miRDB
PNMA3	mRNA_set2	NA	NA	miRDB
AKT3	mRNA_set2	NA	NA	miRDB
PAFAH1B2	mRNA_set2	NA	NA	miRDB
BEST3	mRNA_set2	NA	NA	miRDB
TXNDC9	mRNA_set2	NA	NA	miRDB
ABI1	mRNA_set2	NA	NA	miRDB
MLLT3	mRNA_set2	NA	NA	miRDB
CDKN2AIP	mRNA_set2	NA	NA	miRDB
PPP1R2	mRNA_set2	NA	NA	miRDB
NRAS	mRNA_set2	NA	NA	miRDB
GK	mRNA_set2	NA	NA	miRDB
CIP2A	mRNA_set2	NA	NA	miRDB
UBE2S	mRNA_set2	NA	NA	miRDB
MYCBP2	mRNA_set2	NA	NA	miRDB
DPH3P1	mRNA_set2	NA	NA	miRDB
RGPD1	mRNA_set2	NA	NA	miRDB
ANK3	mRNA_set2	NA	NA	miRDB
NR4A3	mRNA_set2	NA	NA	miRDB
TYW3	mRNA_set2	NA	NA	miRDB
SEPSECS	mRNA_set2	NA	NA	miRDB
CNEP1R1	mRNA_set2	NA	NA	miRDB
ANKRD28	mRNA_set2	NA	NA	miRDB
KLHL2	mRNA_set2	NA	NA	miRDB
C4orf3	mRNA_set2	NA	NA	miRDB
ARL5A	mRNA_set2	NA	NA	miRDB

ATP11B	mRNA_set2	NA	NA	miRDB
GORASP2	mRNA_set2	NA	NA	miRDB
CLVS1	mRNA_set2	NA	NA	miRDB
MINDY3	mRNA_set2	NA	NA	miRDB
HAND2	mRNA_set2	NA	NA	miRDB
ACTN2	mRNA_set2	NA	NA	miRDB
MYH10	mRNA_set2	NA	NA	miRDB
PRKCB	mRNA_set2	NA	NA	miRDB
SCN1A	mRNA_set2	NA	NA	miRDB
ZFAND3	mRNA_set2	NA	NA	miRDB
SPTLC2	mRNA_set2	NA	NA	miRDB
BNIP3	mRNA_set2	NA	NA	miRDB
IRF4	mRNA_set2	NA	NA	miRDB
HIP1	mRNA_set2	NA	NA	miRDB
SLC9A7	mRNA_set2	NA	NA	miRDB
FAM171A1	mRNA_set2	NA	NA	miRDB
NR3C1	mRNA_set2	NA	NA	miRDB
KCNN3	mRNA_set2	NA	NA	miRDB
CDH5	mRNA_set2	NA	NA	miRDB
HS2ST1	mRNA_set2	NA	NA	miRDB
TMPRSS7	mRNA_set2	NA	NA	miRDB
NR2C2	mRNA_set2	NA	NA	miRDB
NETO1	mRNA_set2	NA	NA	miRDB
NREP	mRNA_set2	NA	NA	miRDB
TTC39A	mRNA_set2	NA	NA	miRDB
ADAMTSL1	mRNA_set2	NA	NA	miRDB
LARP4	mRNA_set2	NA	NA	miRDB
ATXN1	mRNA_set2	NA	NA	miRDB

SLC26A4	mRNA_set2	NA	NA	miRDB
RAB14	mRNA_set2	NA	NA	miRDB
GPCPD1	mRNA_set2	NA	NA	miRDB
PRNP	mRNA_set2	NA	NA	miRDB
TMEM266	mRNA_set2	NA	NA	miRDB
PIGS	mRNA_set2	NA	NA	miRDB
ADCY2	mRNA_set2	NA	NA	miRDB
CADM1	mRNA_set2	NA	NA	miRDB
RFX7	mRNA_set2	NA	NA	miRDB
AHDC1	mRNA_set2	NA	NA	miRDB
LYSMD2	mRNA_set2	NA	NA	miRDB
FAM84A	mRNA_set2	NA	NA	miRDB
NT5C1B-RDH14	mRNA_set2	NA	NA	miRDB
PCARE	mRNA_set2	NA	NA	miRDB
PRKACB	mRNA_set2	NA	NA	miRDB
KCNJ2	mRNA_set2	NA	NA	miRDB
EPHB1	mRNA_set2	NA	NA	miRDB
CREM	mRNA_set2	NA	NA	miRDB
TAF1B	mRNA_set2	NA	NA	miRDB
ATG13	mRNA_set2	NA	NA	miRDB
BCL7A	mRNA_set2	NA	NA	miRDB
DACH1	mRNA_set2	NA	NA	miRDB
LIPI	mRNA_set2	NA	NA	miRDB
CCDC170	mRNA_set2	NA	NA	miRDB
TNS3	mRNA_set2	NA	NA	miRDB
ASRGL1	mRNA_set2	NA	NA	miRDB
PPP4R3B	mRNA_set2	NA	NA	miRDB
SRSF1	mRNA_set2	NA	NA	miRDB

TPM4	mRNA_set2	NA	NA	miRDB
RFLNB	mRNA_set2	NA	NA	miRDB
KCNMB2	mRNA_set2	NA	NA	miRDB
LRRC10	mRNA_set2	NA	NA	miRDB
ATOH8	mRNA_set2	NA	NA	miRDB
PAX5	mRNA_set2	NA	NA	miRDB
SRP9	mRNA_set2	NA	NA	miRDB
ABHD14A-ACY1	mRNA_set2	NA	NA	miRDB
TANC2	mRNA_set2	NA	NA	miRDB
LDLRAD4	mRNA_set2	NA	NA	miRDB
CALM1	mRNA_set2	NA	NA	miRDB
MGA	mRNA_set2	NA	NA	miRDB
SEC22C	mRNA_set2	NA	NA	miRDB
MFAP3L	mRNA_set2	NA	NA	miRDB
CCL20	mRNA_set2	NA	NA	miRDB
ZNF407	mRNA_set2	NA	NA	miRDB
PPM1A	mRNA_set2	NA	NA	miRDB
COL4A4	mRNA_set2	NA	NA	miRDB
KLF13	mRNA_set2	NA	NA	miRDB
CSMD3	mRNA_set2	NA	NA	miRDB
ZC3H6	mRNA_set2	NA	NA	miRDB
KCNK9	mRNA_set2	NA	NA	miRDB
DAAM1	mRNA_set2	NA	NA	miRDB
KIF11	mRNA_set2	NA	NA	miRDB
ACVR2A	mRNA_set2	NA	NA	miRDB
UTP25	mRNA_set2	NA	NA	miRDB
SKIL	mRNA_set2	NA	NA	miRDB
AIFM1	mRNA_set2	NA	NA	miRDB

SPIRE1	mRNA_set2	NA	NA	miRDB
JPT2	mRNA_set2	NA	NA	miRDB
XRN1	mRNA_set2	NA	NA	miRDB
KPNA4	mRNA_set2	NA	NA	miRDB
PDZRN3	mRNA_set2	NA	NA	miRDB
MAP3K20	mRNA_set2	NA	NA	miRDB
EP300	mRNA_set2	NA	NA	miRDB
RBMS3	mRNA_set2	NA	NA	miRDB
NDFIP2	mRNA_set2	NA	NA	miRDB
NPHP1	mRNA_set2	NA	NA	miRDB
MAPKAPK5	mRNA_set2	NA	NA	miRDB
PDE8B	mRNA_set2	NA	NA	miRDB
CNKSR2	mRNA_set2	NA	NA	miRDB
ITPRIPL2	mRNA_set2	NA	NA	miRDB
BCL11B	mRNA_set2	NA	NA	miRDB
EIF4E3	mRNA_set2	NA	NA	miRDB
SRGAP1	mRNA_set2	NA	NA	miRDB
IRS1	mRNA_set2	NA	NA	miRDB
LIMK2	mRNA_set2	NA	NA	miRDB
AMACR	mRNA_set2	NA	NA	miRDB
TIPRL	mRNA_set2	NA	NA	miRDB
TRMT5	mRNA_set2	NA	NA	miRDB
CPNE4	mRNA_set2	NA	NA	miRDB
OXR1	mRNA_set2	NA	NA	miRDB
KAT2B	mRNA_set2	NA	NA	miRDB
DCAF7	mRNA_set2	NA	NA	miRDB
RCBTB1	mRNA_set2	NA	NA	miRDB
UBE2N	mRNA_set2	NA	NA	miRDB

FBXO45	mRNA_set2	NA	NA	miRDB
ZNF705E	mRNA_set2	NA	NA	miRDB
SLC5A7	mRNA_set2	NA	NA	miRDB
FAM76B	mRNA_set2	NA	NA	miRDB
KBTBD8	mRNA_set2	NA	NA	miRDB
TMEM184A	mRNA_set2	NA	NA	miRDB
FLRT3	mRNA_set2	NA	NA	miRDB
BNIP2	mRNA_set2	NA	NA	miRDB
SMAD4	mRNA_set2	NA	NA	miRDB
GRAMD1B	mRNA_set2	NA	NA	miRDB
CECR2	mRNA_set2	NA	NA	miRDB
ECE2	mRNA_set2	NA	NA	miRDB
HYOU1	mRNA_set2	NA	NA	miRDB
TRAF3	mRNA_set2	NA	NA	miRDB
SERBP1	mRNA_set2	NA	NA	miRDB
UHRF1BP1	mRNA_set2	NA	NA	miRDB
EIF5A2	mRNA_set2	NA	NA	miRDB
EEF1AKMT4-ECE2	mRNA_set2	NA	NA	miRDB
ZNF84	mRNA_set2	NA	NA	miRDB
TBR1	mRNA_set2	NA	NA	miRDB
NF1	mRNA_set2	NA	NA	miRDB
ZIC5	mRNA_set2	NA	NA	miRDB
FUBP3	mRNA_set2	NA	NA	miRDB
BRPF1	mRNA_set2	NA	NA	miRDB
CTSA	mRNA_set2	NA	NA	miRDB
KIF14	mRNA_set2	NA	NA	miRDB
CEP55	mRNA_set2	NA	NA	miRDB
PTGES3	mRNA_set2	NA	NA	miRDB

ADAM10	mRNA_set2	NA	NA	miRDB
SIRT7	mRNA_set2	NA	NA	miRDB
TFRC	mRNA_set2	NA	NA	miRDB
ZNRF1	mRNA_set2	NA	NA	miRDB
WDFY4	mRNA_set2	NA	NA	miRDB
ISM1	mRNA_set2	NA	NA	miRDB
IPO8	mRNA_set2	NA	NA	miRDB
PARP11	mRNA_set2	NA	NA	miRDB
ADCY1	mRNA_set2	NA	NA	miRDB
PTPN14	mRNA_set2	NA	NA	miRDB
CLC	mRNA_set2	NA	NA	miRDB
ZNF518A	mRNA_set2	NA	NA	miRDB
CCNT1	mRNA_set2	NA	NA	miRDB
RHOQ	mRNA_set2	NA	NA	miRDB
GNAZ	mRNA_set2	NA	NA	miRDB
ENPEP	mRNA_set2	NA	NA	miRDB
LRRC40	mRNA_set2	NA	NA	miRDB
TAOK1	mRNA_set2	NA	NA	miRDB
GPC6	mRNA_set2	NA	NA	miRDB
ABHD13	mRNA_set2	NA	NA	miRDB
HOOK3	mRNA_set2	NA	NA	miRDB
ITGB6	mRNA_set2	NA	NA	miRDB
ACAP2	mRNA_set2	NA	NA	miRDB
ARHGEF28	mRNA_set2	NA	NA	miRDB
FOXG1	mRNA_set2	NA	NA	miRDB
STX7	mRNA_set2	NA	NA	miRDB
MAK16	mRNA_set2	NA	NA	miRDB
SYNJ2BP	mRNA_set2	NA	NA	miRDB

HPCAL1	mRNA_set2	NA	NA	miRDB
C2orf66	mRNA_set2	NA	NA	miRDB
UBA5	mRNA_set2	NA	NA	miRDB
IFNB1	mRNA_set2	NA	NA	miRDB
BAIAP2L1	mRNA_set2	NA	NA	miRDB
CALCR	mRNA_set2	NA	NA	miRDB
RCOR3	mRNA_set2	NA	NA	miRDB
VAPB	mRNA_set2	NA	NA	miRDB
ZBTB42	mRNA_set2	NA	NA	miRDB
LONP2	mRNA_set2	NA	NA	miRDB
TMEM68	mRNA_set2	NA	NA	miRDB
RNF182	mRNA_set2	NA	NA	miRDB
SFSWAP	mRNA_set2	NA	NA	miRDB
ISL1	mRNA_set2	NA	NA	miRDB
PPP1CC	mRNA_set2	NA	NA	miRDB
CHD2	mRNA_set2	NA	NA	miRDB
SMAD5	mRNA_set2	NA	NA	miRDB
CBFA2T3	mRNA_set2	NA	NA	miRDB
TICRR	mRNA_set2	NA	NA	miRDB
PPIF	mRNA_set2	NA	NA	miRDB
EDAR	mRNA_set2	NA	NA	miRDB
NR1D2	mRNA_set2	NA	NA	miRDB
PRKAA2	mRNA_set2	NA	NA	miRDB
CLCN5	mRNA_set2	NA	NA	miRDB
TRERF1	mRNA_set2	NA	NA	miRDB
DCP1A	mRNA_set2	NA	NA	miRDB
KCMF1	mRNA_set2	NA	NA	miRDB
FAM206A	mRNA_set2	NA	NA	miRDB

FGD6	mRNA_set2	NA	NA	miRDB
SNX3	mRNA_set2	NA	NA	miRDB
TAF4	mRNA_set2	NA	NA	miRDB
ST8SIA3	mRNA_set2	NA	NA	miRDB
MTMR12	mRNA_set2	NA	NA	miRDB
SNAPIN	mRNA_set2	NA	NA	miRDB
SKP1	mRNA_set2	NA	NA	miRDB
GABPB2	mRNA_set2	NA	NA	miRDB
TRIP12	mRNA_set2	NA	NA	miRDB
GOLT1B	mRNA_set2	NA	NA	miRDB
KCND3	mRNA_set2	NA	NA	miRDB
CRISP1	mRNA_set2	NA	NA	miRDB
RBFOX2	mRNA_set2	NA	NA	miRDB
SERPINE1	mRNA_set2	NA	NA	miRDB
SOX5	mRNA_set2	NA	NA	miRDB
C2orf91	mRNA_set2	NA	NA	miRDB
PLEKHA1	mRNA_set2	NA	NA	miRDB
GLS	mRNA_set2	NA	NA	miRDB
PDE12	mRNA_set2	NA	NA	miRDB
TCF7L2	mRNA_set2	NA	NA	miRDB
SNED1	mRNA_set2	NA	NA	miRDB
PSMA7	mRNA_set2	NA	NA	miRDB
SMIM2	mRNA_set2	NA	NA	miRDB
SH3BGRL3	mRNA_set2	NA	NA	miRDB
ORC6	mRNA_set2	NA	NA	miRDB
BAG4	mRNA_set2	NA	NA	miRDB
RBM20	mRNA_set2	NA	NA	miRDB
NECTIN2	mRNA_set2	NA	NA	miRDB

UBE2T	mRNA_set2	NA	NA	miRDB
LYRM2	mRNA_set2	NA	NA	miRDB
SHROOM4	mRNA_set2	NA	NA	miRDB
TMEM47	mRNA_set2	NA	NA	miRDB
WWC2	mRNA_set2	NA	NA	miRDB
LYPD6	mRNA_set2	NA	NA	miRDB
KLF2	mRNA_set2	NA	NA	miRDB
GABRB2	mRNA_set2	NA	NA	miRDB
SYT13	mRNA_set2	NA	NA	miRDB
ZFP14	mRNA_set2	NA	NA	miRDB
TRMT9B	mRNA_set2	NA	NA	miRDB
KCNJ16	mRNA_set2	NA	NA	miRDB
RAB3C	mRNA_set2	NA	NA	miRDB
WSB2	mRNA_set2	NA	NA	miRDB
TMEM43	mRNA_set2	NA	NA	miRDB
CDC73	mRNA_set2	NA	NA	miRDB
TMEM100	mRNA_set2	NA	NA	miRDB
CARF	mRNA_set2	NA	NA	miRDB
PRRC1	mRNA_set2	NA	NA	miRDB
MORF4L2	mRNA_set2	NA	NA	miRDB
APTX	mRNA_set2	NA	NA	miRDB
TUFT1	mRNA_set2	NA	NA	miRDB
DMTF1	mRNA_set2	NA	NA	miRDB
CMTM6	mRNA_set2	NA	NA	miRDB
CMPK2	mRNA_set2	NA	NA	miRDB
TMED5	mRNA_set2	NA	NA	miRDB
SPATA6L	mRNA_set2	NA	NA	miRDB
PCDH17	mRNA_set2	NA	NA	miRDB

RABGGTB	mRNA_set2	NA	NA	miRDB
ZNF75A	mRNA_set2	NA	NA	miRDB
GALNT1	mRNA_set2	NA	NA	miRDB
PDZK1IP1	mRNA_set2	NA	NA	miRDB
PTPRT	mRNA_set2	NA	NA	miRDB
TMTC4	mRNA_set2	NA	NA	miRDB
GTF2A1	mRNA_set2	NA	NA	miRDB
SEC24A	mRNA_set2	NA	NA	miRDB
PPARA	mRNA_set2	NA	NA	miRDB
RXRA	mRNA_set2	NA	NA	miRDB
NR6A1	mRNA_set2	NA	NA	miRDB
CAPZA1	mRNA_set2	NA	NA	miRDB
NFIA	mRNA_set2	NA	NA	miRDB
CABLES2	mRNA_set2	NA	NA	miRDB
NCBP3	mRNA_set2	NA	NA	miRDB
NRCAM	mRNA_set2	NA	NA	miRDB
TXN2	mRNA_set2	NA	NA	miRDB
ACLY	mRNA_set2	NA	NA	miRDB
IFNAR1	mRNA_set2	NA	NA	miRDB
COL6A3	mRNA_set2	NA	NA	miRDB
CHD1	mRNA_set2	NA	NA	miRDB
RAB10	mRNA_set2	NA	NA	miRDB
ZNF3	mRNA_set2	NA	NA	miRDB
PPP1R12A	mRNA_set2	NA	NA	miRDB
SH2B3	mRNA_set2	NA	NA	miRDB
CDK8	mRNA_set2	NA	NA	miRDB
NFYA	mRNA_set2	NA	NA	miRDB
CAST	mRNA_set2	NA	NA	miRDB

ITGA11	mRNA_set2	NA	NA	miRDB
FBXL19	mRNA_set2	NA	NA	miRDB
BAHCC1	mRNA_set2	NA	NA	miRDB
MAPKBP1	mRNA_set2	NA	NA	miRDB
TSPAN9	mRNA_set2	NA	NA	miRDB
PGD	mRNA_set2	NA	NA	miRDB
TSPYL4	mRNA_set2	NA	NA	miRDB
MMP8	mRNA_set2	NA	NA	miRDB
SLC35B4	mRNA_set2	NA	NA	miRDB
CSTA	mRNA_set2	NA	NA	miRDB
ZBTB21	mRNA_set2	NA	NA	miRDB
SPATS2L	mRNA_set2	NA	NA	miRDB
NIN	mRNA_set2	NA	NA	miRDB
KDELR2	mRNA_set2	NA	NA	miRDB
ANKIB1	mRNA_set2	NA	NA	miRDB
SLCO1B1	mRNA_set2	NA	NA	miRDB
ARPC1A	mRNA_set2	NA	NA	miRDB
HACD4	mRNA_set2	NA	NA	miRDB
LRRC29	mRNA_set2	NA	NA	miRDB
LCP1	mRNA_set2	NA	NA	miRDB
APH1B	mRNA_set2	NA	NA	miRDB
CLDN2	mRNA_set2	NA	NA	miRDB
STK24	mRNA_set2	NA	NA	miRDB
SLC6A8	mRNA_set2	NA	NA	miRDB
DPEP2NB	mRNA_set2	NA	NA	miRDB
NSD3	mRNA_set2	NA	NA	miRDB
TMEM132D	mRNA_set2	NA	NA	miRDB
CCSER1	mRNA_set2	NA	NA	miRDB

GVQW2	mRNA_set2	NA	NA	miRDB
FAM92A	mRNA_set2	NA	NA	miRDB
MACROD2	mRNA_set2	NA	NA	miRDB
CDC5L	mRNA_set2	NA	NA	miRDB
CEP104	mRNA_set2	NA	NA	miRDB
LIMCH1	mRNA_set2	NA	NA	miRDB
UPP2	mRNA_set2	NA	NA	miRDB
C1GALT1C1	mRNA_set2	NA	NA	miRDB
ZBTB24	mRNA_set2	NA	NA	miRDB
ENOX2	mRNA_set2	NA	NA	miRDB
HOXA9	mRNA_set2	NA	NA	miRDB
PRSS22	mRNA_set2	NA	NA	miRDB
APOL6	mRNA_set2	NA	NA	miRDB
FYTTD1	mRNA_set2	NA	NA	miRDB
VDAC3	mRNA_set2	NA	NA	miRDB
HMGCS1	mRNA_set2	NA	NA	miRDB
NMB	mRNA_set2	NA	NA	miRDB
FRYL	mRNA_set2	NA	NA	miRDB
B9D1	mRNA_set2	NA	NA	miRDB
MFSD6	mRNA_set2	NA	NA	miRDB
MINPP1	mRNA_set2	NA	NA	miRDB
DMRT3	mRNA_set2	NA	NA	miRDB
ZBTB44	mRNA_set2	NA	NA	miRDB
HOXA13	mRNA_set2	NA	NA	miRDB
AEBP2	mRNA_set2	NA	NA	miRDB
RAP1B	mRNA_set2	NA	NA	miRDB
CCDC149	mRNA_set2	NA	NA	miRDB
AIDA	mRNA_set2	NA	NA	miRDB

EPHB2	mRNA_set2	NA	NA	miRDB
PRTG	mRNA_set2	NA	NA	miRDB
ZNF230	mRNA_set2	NA	NA	miRDB
FAM84B	mRNA_set2	NA	NA	miRDB
SSBP2	mRNA_set2	NA	NA	miRDB
CSRNP1	mRNA_set2	NA	NA	miRDB
CBFB	mRNA_set2	NA	NA	miRDB
SMPD3	mRNA_set2	NA	NA	miRDB
MTMR1	mRNA_set2	NA	NA	miRDB
TBP	mRNA_set2	NA	NA	miRDB
DLEU7	mRNA_set2	NA	NA	miRDB
C12orf4	mRNA_set2	NA	NA	miRDB
KIT	mRNA_set2	NA	NA	miRDB
CDK1	mRNA_set2	NA	NA	miRDB
USP7	mRNA_set2	NA	NA	miRDB
DIPK2A	mRNA_set2	NA	NA	miRDB
LUC7L	mRNA_set2	NA	NA	miRDB
MYO1D	mRNA_set2	NA	NA	miRDB
SELENOT	mRNA_set2	NA	NA	miRDB
STT3A	mRNA_set2	NA	NA	miRDB
ATP6V1B2	mRNA_set2	NA	NA	miRDB
BOLL	mRNA_set2	NA	NA	miRDB
PRSS21	mRNA_set2	NA	NA	miRDB
LIX1	mRNA_set2	NA	NA	miRDB
FGF14	mRNA_set2	NA	NA	miRDB
CXCR4	mRNA_set2	NA	NA	miRDB
ATXN1L	mRNA_set2	NA	NA	miRDB
TMOD2	mRNA_set2	NA	NA	miRDB

C12orf49	mRNA_set2	NA	NA	miRDB
VEGFA	mRNA_set2	NA	NA	miRDB
PGM2	mRNA_set2	NA	NA	miRDB
AP3D1	mRNA_set2	NA	NA	miRDB
TACR1	mRNA_set2	NA	NA	miRDB
MAP3K1	mRNA_set2	NA	NA	miRDB
ASXL2	mRNA_set2	NA	NA	miRDB
BSN	mRNA_set2	NA	NA	miRDB
COL25A1	mRNA_set2	NA	NA	miRDB
CORO1B	mRNA_set2	NA	NA	miRDB
HOXB4	mRNA_set2	NA	NA	miRDB
SLC38A2	mRNA_set2	NA	NA	miRDB
CYGB	mRNA_set2	NA	NA	miRDB
TBC1D22B	mRNA_set2	NA	NA	miRDB
ATXN3L	mRNA_set2	NA	NA	miRDB
CD47	mRNA_set2	NA	NA	miRDB
AVPR2	mRNA_set2	NA	NA	miRDB
HINFP	mRNA_set2	NA	NA	miRDB
CDC27	mRNA_set2	NA	NA	miRDB
ZNF398	mRNA_set2	NA	NA	miRDB
STYX	mRNA_set2	NA	NA	miRDB
SYT6	mRNA_set2	NA	NA	miRDB
FASLG	mRNA_set2	NA	NA	miRDB
PTPN2	mRNA_set2	NA	NA	miRDB
CPNE8	mRNA_set2	NA	NA	miRDB
CC2D2A	mRNA_set2	NA	NA	miRDB
EPM2AIP1	mRNA_set2	NA	NA	miRDB
ARHGAP5	mRNA_set2	NA	NA	miRDB

MANEA	mRNA_set2	NA	NA	miRDB
DNAH5	mRNA_set2	NA	NA	miRDB
LRRN1	mRNA_set2	NA	NA	miRDB
ZBTB33	mRNA_set2	NA	NA	miRDB
MTHFD1	mRNA_set2	NA	NA	miRDB
TMTC3	mRNA_set2	NA	NA	miRDB
EGLN3	mRNA_set2	NA	NA	miRDB
LARGE2	mRNA_set2	NA	NA	miRDB
CEP350	mRNA_set2	NA	NA	miRDB
PLEKHA2	mRNA_set2	NA	NA	miRDB
TENM3	mRNA_set2	NA	NA	miRDB
PPM1L	mRNA_set2	NA	NA	miRDB
GMNC	mRNA_set2	NA	NA	miRDB
DCK	mRNA_set2	NA	NA	miRDB
KIAA1147	mRNA_set2	NA	NA	miRDB
EIF5	mRNA_set2	NA	NA	miRDB
SH3RF1	mRNA_set2	NA	NA	miRDB
GFPT1	mRNA_set2	NA	NA	miRDB
SEC22A	mRNA_set2	NA	NA	miRDB
RHOT1	mRNA_set2	NA	NA	miRDB
DGKG	mRNA_set2	NA	NA	miRDB
SPATA16	mRNA_set2	NA	NA	miRDB
FAM98A	mRNA_set2	NA	NA	miRDB
INPP4A	mRNA_set2	NA	NA	miRDB
CDC42BPB	mRNA_set2	NA	NA	miRDB
8-Sep	mRNA_set2	NA	NA	miRDB
MPP6	mRNA_set2	NA	NA	miRDB
MAP3K14	mRNA_set2	NA	NA	miRDB

CNTLN	mRNA_set2	NA	NA	miRDB
HDX	mRNA_set2	NA	NA	miRDB
BMI1	mRNA_set2	NA	NA	miRDB
EFHD2	mRNA_set2	NA	NA	miRDB
MDFI	mRNA_set2	NA	NA	miRDB
ICOS	mRNA_set2	NA	NA	miRDB
DPY19L4	mRNA_set2	NA	NA	miRDB
STRBP	mRNA_set2	NA	NA	miRDB
MEPCE	mRNA_set2	NA	NA	miRDB
DCX	mRNA_set2	NA	NA	miRDB
GNPNAT1	mRNA_set2	NA	NA	miRDB
SLC16A10	mRNA_set2	NA	NA	miRDB
ZNF780B	mRNA_set2	NA	NA	miRDB
FAM199X	mRNA_set2	NA	NA	miRDB
TMEM248	mRNA_set2	NA	NA	miRDB
ST18	mRNA_set2	NA	NA	miRDB
DYRK1B	mRNA_set2	NA	NA	miRDB
MARF1	mRNA_set2	NA	NA	miRDB
TAF4B	mRNA_set2	NA	NA	miRDB
PSMD9	mRNA_set2	NA	NA	miRDB
USP6	mRNA_set2	NA	NA	miRDB
TMEM121B	mRNA_set2	NA	NA	miRDB
PTEN	mRNA_set2	NA	NA	miRDB
PAG1	mRNA_set2	NA	NA	miRDB
PKHD1	mRNA_set2	NA	NA	miRDB
KDM2B	mRNA_set2	NA	NA	miRDB
MTMR6	mRNA_set2	NA	NA	miRDB
AGBL2	mRNA_set2	NA	NA	miRDB

UBN2	mRNA_set2	NA	NA	miRDB
LILRB4	mRNA_set2	NA	NA	miRDB
STX3	mRNA_set2	NA	NA	miRDB
2-Sep	mRNA_set2	NA	NA	miRDB
GLIS2	mRNA_set2	NA	NA	miRDB
PAK3	mRNA_set2	NA	NA	miRDB
SPEG	mRNA_set2	NA	NA	miRDB
SFRP2	mRNA_set2	NA	NA	miRDB
TRPS1	mRNA_set2	NA	NA	miRDB
HDAC4	mRNA_set2	NA	NA	miRDB
ANKRD23	mRNA_set2	NA	NA	miRDB
SYN3	mRNA_set2	NA	NA	miRDB
DLG4	mRNA_set2	NA	NA	miRDB
IGSF11	mRNA_set2	NA	NA	miRDB
TAOK3	mRNA_set2	NA	NA	miRDB
AJAP1	mRNA_set2	NA	NA	miRDB
CNOT2	mRNA_set2	NA	NA	miRDB
PPP1CB	mRNA_set2	NA	NA	miRDB
EBF3	mRNA_set2	NA	NA	miRDB
BACE1	mRNA_set2	NA	NA	miRDB
SLC12A6	mRNA_set2	NA	NA	miRDB
NPTXR	mRNA_set2	NA	NA	miRDB
EML1	mRNA_set2	NA	NA	miRDB
UBR3	mRNA_set2	NA	NA	miRDB
CAMSAP1	mRNA_set2	NA	NA	miRDB
TLN1	mRNA_set2	NA	NA	miRDB
SAMD12	mRNA_set2	NA	NA	miRDB
RANBP17	mRNA_set2	NA	NA	miRDB

ZNF138	mRNA_set2	NA	NA	miRDB
SDHC	mRNA_set2	NA	NA	miRDB
C1orf112	mRNA_set2	NA	NA	miRDB
FAM19A5	mRNA_set2	NA	NA	miRDB
MBNL3	mRNA_set2	NA	NA	miRDB
FAM118B	mRNA_set2	NA	NA	miRDB
LRCH2	mRNA_set2	NA	NA	miRDB
AKAP17A	mRNA_set2	NA	NA	miRDB
DPY19L3	mRNA_set2	NA	NA	miRDB
NAA16	mRNA_set2	NA	NA	miRDB
WDR60	mRNA_set2	NA	NA	miRDB
STAR	mRNA_set2	NA	NA	miRDB
RAB7A	mRNA_set2	NA	NA	miRDB
AGPAT4	mRNA_set2	NA	NA	miRDB
RAB31	mRNA_set2	NA	NA	miRDB
GBP6	mRNA_set2	NA	NA	miRDB
DNTTIP2	mRNA_set2	NA	NA	miRDB
ABCF3	mRNA_set2	NA	NA	miRDB
VTA1	mRNA_set2	NA	NA	miRDB
ODAPH	mRNA_set2	NA	NA	miRDB
TRPM7	mRNA_set2	NA	NA	miRDB
PRRX1	mRNA_set2	NA	NA	miRDB
AGGF1	mRNA_set2	NA	NA	miRDB
SLC35F3	mRNA_set2	NA	NA	miRDB
ANKRD40	mRNA_set2	NA	NA	miRDB
RSAD2	mRNA_set2	NA	NA	miRDB
ITGA2	mRNA_set2	NA	NA	miRDB
IGF2BP3	mRNA_set2	NA	NA	miRDB

PGM2L1	mRNA_set2	NA	NA	miRDB
ABAT	mRNA_set2	NA	NA	miRDB
VAT1L	mRNA_set2	NA	NA	miRDB
VPS13A	mRNA_set2	NA	NA	miRDB
BRSK1	mRNA_set2	NA	NA	miRDB
WNT3A	mRNA_set2	NA	NA	miRDB
C1orf21	mRNA_set2	NA	NA	miRDB
LCN8	mRNA_set2	NA	NA	miRDB
PDGFRA	mRNA_set2	NA	NA	miRDB
AGPAT3	mRNA_set2	NA	NA	miRDB
HOXC11	mRNA_set2	NA	NA	miRDB
FAM193B	mRNA_set2	NA	NA	miRDB
RAP2B	mRNA_set2	NA	NA	miRDB
ZNF148	mRNA_set2	NA	NA	miRDB
CPEB3	mRNA_set2	NA	NA	miRDB
IKZF1	mRNA_set2	NA	NA	miRDB
ANKRD36B	mRNA_set2	NA	NA	miRDB
FAM149A	mRNA_set2	NA	NA	miRDB
ATG4D	mRNA_set2	NA	NA	miRDB
ELF5	mRNA_set2	NA	NA	miRDB
DDAH1	mRNA_set2	NA	NA	miRDB
SLC12A7	mRNA_set2	NA	NA	miRDB
KRR1	mRNA_set2	NA	NA	miRDB
PPARGC1B	mRNA_set2	NA	NA	miRDB
MAFF	mRNA_set2	NA	NA	miRDB
TFDP2	mRNA_set2	NA	NA	miRDB
RHD	mRNA_set2	NA	NA	miRDB
ZNF260	mRNA_set2	NA	NA	miRDB

GDF6	mRNA_set2	NA	NA	miRDB
NAMPT	mRNA_set2	NA	NA	miRDB
C2CD5	mRNA_set2	NA	NA	miRDB
TRIM31	mRNA_set2	NA	NA	miRDB
ELOF1	mRNA_set2	NA	NA	miRDB
TEX2	mRNA_set2	NA	NA	miRDB
NOTCH3	mRNA_set2	NA	NA	miRDB
SYAP1	mRNA_set2	NA	NA	miRDB
RNF213	mRNA_set2	NA	NA	miRDB
ZNF226	mRNA_set2	NA	NA	miRDB
CEP112	mRNA_set2	NA	NA	miRDB
TSPOAP1	mRNA_set2	NA	NA	miRDB
HMGN3	mRNA_set2	NA	NA	miRDB
ANKDD1B	mRNA_set2	NA	NA	miRDB
SAFB2	mRNA_set2	NA	NA	miRDB
SMYD4	mRNA_set2	NA	NA	miRDB
ZNF236	mRNA_set2	NA	NA	miRDB
TMEM132C	mRNA_set2	NA	NA	miRDB
RUBCN	mRNA_set2	NA	NA	miRDB
NAALADL1	mRNA_set2	NA	NA	miRDB
DTHD1	mRNA_set2	NA	NA	miRDB
NAA11	mRNA_set2	NA	NA	miRDB
LYRM9	mRNA_set2	NA	NA	miRDB
SETBP1	mRNA_set2	NA	NA	miRDB
ZNF730	mRNA_set2	NA	NA	miRDB
EIF4A1	mRNA_set2	NA	NA	miRDB
AFTPH	mRNA_set2	NA	NA	miRDB
GABPB1	mRNA_set2	NA	NA	miRDB

ZFHX4	mRNA_set2	NA	NA	miRDB
LPGAT1	mRNA_set2	NA	NA	miRDB
IGF2BP2	mRNA_set2	NA	NA	miRDB
MORN2	mRNA_set2	NA	NA	miRDB
SLC26A7	mRNA_set2	NA	NA	miRDB
NSMCE4A	mRNA_set2	NA	NA	miRDB
SLITRK2	mRNA_set2	NA	NA	miRDB
VGLL3	mRNA_set2	NA	NA	miRDB
HCN1	mRNA_set2	NA	NA	miRDB
TP53I13	mRNA_set2	NA	NA	miRDB
PDZD8	mRNA_set2	NA	NA	miRDB
SIRT1	mRNA_set2	NA	NA	miRDB
IMPAD1	mRNA_set2	NA	NA	miRDB
ITPR3	mRNA_set2	NA	NA	miRDB
CALCRL	mRNA_set2	NA	NA	miRDB
SIPA1L1	mRNA_set2	NA	NA	miRDB
HSPA13	mRNA_set2	NA	NA	miRDB
FAM49A	mRNA_set2	NA	NA	miRDB
MAN1A1	mRNA_set2	NA	NA	miRDB
ZNF37A	mRNA_set2	NA	NA	miRDB
SUSD5	mRNA_set2	NA	NA	miRDB
FAM81A	mRNA_set2	NA	NA	miRDB
JAG2	mRNA_set2	NA	NA	miRDB
FZD3	mRNA_set2	NA	NA	miRDB
STK4	mRNA_set2	NA	NA	miRDB
SLC16A7	mRNA_set2	NA	NA	miRDB
PRDM1	mRNA_set2	NA	NA	miRDB
RBBP5	mRNA_set2	NA	NA	miRDB

TBC1D4	mRNA_set2	NA	NA	miRDB
PTCHD1	mRNA_set2	NA	NA	miRDB
ABHD5	mRNA_set2	NA	NA	miRDB
ALKAL1	mRNA_set2	NA	NA	miRDB
BEND3	mRNA_set2	NA	NA	miRDB
STXBP6	mRNA_set2	NA	NA	miRDB
ENOX1	mRNA_set2	NA	NA	miRDB
C2CD4A	mRNA_set2	NA	NA	miRDB
NEBL	mRNA_set2	NA	NA	miRDB
LRBA	mRNA_set2	NA	NA	miRDB
PRPSAP2	mRNA_set2	NA	NA	miRDB
LMLN	mRNA_set2	NA	NA	miRDB
ADAM19	mRNA_set2	NA	NA	miRDB
VEGFC	mRNA_set2	NA	NA	miRDB
KIF3A	mRNA_set2	NA	NA	miRDB
PCDH7	mRNA_set2	NA	NA	miRDB
DPP4	mRNA_set2	NA	NA	miRDB
NPTX2	mRNA_set2	NA	NA	miRDB
FAM13B	mRNA_set2	NA	NA	miRDB
SYT10	mRNA_set2	NA	NA	miRDB
CHST1	mRNA_set2	NA	NA	miRDB
UBE2W	mRNA_set2	NA	NA	miRDB
DSC2	mRNA_set2	NA	NA	miRDB
TRPM5	mRNA_set2	NA	NA	miRDB
RCC2	mRNA_set2	NA	NA	miRDB
EHMT2	mRNA_set2	NA	NA	miRDB
ZMYND8	mRNA_set2	NA	NA	miRDB
HIC2	mRNA_set2	NA	NA	miRDB

SLC2A13	mRNA_set2	NA	NA	miRDB
MICB	mRNA_set2	NA	NA	miRDB
TRPC7	mRNA_set2	NA	NA	miRDB
MAP1A	mRNA_set2	NA	NA	miRDB
HIVEP2	mRNA_set2	NA	NA	miRDB
PTBP1	mRNA_set2	NA	NA	miRDB
PPP2R5A	mRNA_set2	NA	NA	miRDB
ZC3H7B	mRNA_set2	NA	NA	miRDB
CD164	mRNA_set2	NA	NA	miRDB
CDC42SE1	mRNA_set2	NA	NA	miRDB
DGKE	mRNA_set2	NA	NA	miRDB
ZNF580	mRNA_set2	NA	NA	miRDB
B3GALT1	mRNA_set2	NA	NA	miRDB
EEF1E1	mRNA_set2	NA	NA	miRDB
STRN3	mRNA_set2	NA	NA	miRDB
UAP1	mRNA_set2	NA	NA	miRDB
MS4A2	mRNA_set2	NA	NA	miRDB
SLC4A1	mRNA_set2	NA	NA	miRDB
KDM5A	mRNA_set2	NA	NA	miRDB
SSB	mRNA_set2	NA	NA	miRDB
BPTF	mRNA_set2	NA	NA	miRDB
CEP126	mRNA_set2	NA	NA	miRDB
BSDC1	mRNA_set2	NA	NA	miRDB
HECW1	mRNA_set2	NA	NA	miRDB
PCSK5	mRNA_set2	NA	NA	miRDB
SLCO4C1	mRNA_set2	NA	NA	miRDB
FAS	mRNA_set2	NA	NA	miRDB
ZMYM1	mRNA_set2	NA	NA	miRDB

THRAP3	mRNA_set2	NA	NA	miRDB
DBF4	mRNA_set2	NA	NA	miRDB
RASA2	mRNA_set2	NA	NA	miRDB
LRRC8B	mRNA_set2	NA	NA	miRDB
DENND6A	mRNA_set2	NA	NA	miRDB
BLOC1S2	mRNA_set2	NA	NA	miRDB
HORMAD2	mRNA_set2	NA	NA	miRDB
BCL10	mRNA_set2	NA	NA	miRDB
ALDOB	mRNA_set2	NA	NA	miRDB
CLEC7A	mRNA_set2	NA	NA	miRDB
SH3BP1	mRNA_set2	NA	NA	miRDB
CCDC106	mRNA_set2	NA	NA	miRDB
PXDC1	mRNA_set2	NA	NA	miRDB
HSBP1	mRNA_set2	NA	NA	miRDB
TMEM67	mRNA_set2	NA	NA	miRDB
TMEM56	mRNA_set2	NA	NA	miRDB
NFIL3	mRNA_set2	NA	NA	miRDB
USP51	mRNA_set2	NA	NA	miRDB
TMEM50A	mRNA_set2	NA	NA	miRDB
EPB41L1	mRNA_set2	NA	NA	miRDB
COX6C	mRNA_set2	NA	NA	miRDB
COLQ	mRNA_set2	NA	NA	miRDB
VANGL1	mRNA_set2	NA	NA	miRDB
LHFPL6	mRNA_set2	NA	NA	miRDB
DIS3	mRNA_set2	NA	NA	miRDB
NEO1	mRNA_set2	NA	NA	miRDB
ZFP1	mRNA_set2	NA	NA	miRDB
NFE2L2	mRNA_set2	NA	NA	miRDB

APBA2	mRNA_set2	NA	NA	miRDB
ADORA1	mRNA_set2	NA	NA	miRDB
CNOT1	mRNA_set2	NA	NA	miRDB
IKZF5	mRNA_set2	NA	NA	miRDB
SNX12	mRNA_set2	NA	NA	miRDB
DUSP5	mRNA_set2	NA	NA	miRDB
JMJD1C	mRNA_set2	NA	NA	miRDB
NFASC	mRNA_set2	NA	NA	miRDB
FAM172A	mRNA_set2	NA	NA	miRDB
PIP5K1B	mRNA_set2	NA	NA	miRDB
ADGRL2	mRNA_set2	NA	NA	miRDB
SH3BGRL2	mRNA_set2	NA	NA	miRDB
MCPH1	mRNA_set2	NA	NA	miRDB
ERMP1	mRNA_set2	NA	NA	miRDB
PIK3R3	mRNA_set2	NA	NA	miRDB
TCF4	mRNA_set2	NA	NA	miRDB
DNER	mRNA_set2	NA	NA	miRDB
ARPP19	mRNA_set2	NA	NA	miRDB
LRP4	mRNA_set2	NA	NA	miRDB
DMPK	mRNA_set2	NA	NA	miRDB
CTSL	mRNA_set2	NA	NA	miRDB
MASP1	mRNA_set2	NA	NA	miRDB
NUDT12	mRNA_set2	NA	NA	miRDB
SCML4	mRNA_set2	NA	NA	miRDB
GAPVD1	mRNA_set2	NA	NA	miRDB
COL2A1	mRNA_set2	NA	NA	miRDB
KLK10	mRNA_set2	NA	NA	miRDB
STXBP4	mRNA_set2	NA	NA	miRDB

DDHD1	mRNA_set2	NA	NA	miRDB
CAGE1	mRNA_set2	NA	NA	miRDB
BCKDHB	mRNA_set2	NA	NA	miRDB
FNBP1L	mRNA_set2	NA	NA	miRDB
SLC45A4	mRNA_set2	NA	NA	miRDB
PIP4P1	mRNA_set2	NA	NA	miRDB
PDCD2	mRNA_set2	NA	NA	miRDB
RASA1	mRNA_set2	NA	NA	miRDB
FGF17	mRNA_set2	NA	NA	miRDB
SRXN1	mRNA_set2	NA	NA	miRDB
ENTPD7	mRNA_set2	NA	NA	miRDB
DAPP1	mRNA_set2	NA	NA	miRDB
PCGF3	mRNA_set2	NA	NA	miRDB
GNG2	mRNA_set2	NA	NA	miRDB
FNIP1	mRNA_set2	NA	NA	miRDB
GREM1	mRNA_set2	NA	NA	miRDB
MAML1	mRNA_set2	NA	NA	miRDB
PSMF1	mRNA_set2	NA	NA	miRDB
FBXO25	mRNA_set2	NA	NA	miRDB
MPPED2	mRNA_set2	NA	NA	miRDB
LOC102724265	mRNA_set2	NA	NA	miRDB
VANGL2	mRNA_set2	NA	NA	miRDB
COMMD10	mRNA_set2	NA	NA	miRDB
HUWE1	mRNA_set2	NA	NA	miRDB
NCMAP	mRNA_set2	NA	NA	miRDB
TBCE	mRNA_set2	NA	NA	miRDB
TMTC1	mRNA_set2	NA	NA	miRDB
ERO1B	mRNA_set2	NA	NA	miRDB

ZNF19	mRNA_set2	NA	NA	miRDB
CLMP	mRNA_set2	NA	NA	miRDB
MYO5A	mRNA_set2	NA	NA	miRDB
NID2	mRNA_set2	NA	NA	miRDB
ZNF568	mRNA_set2	NA	NA	miRDB
KCNJ13	mRNA_set2	NA	NA	miRDB
N4BP1	mRNA_set2	NA	NA	miRDB
GNB5	mRNA_set2	NA	NA	miRDB
FAM227A	mRNA_set2	NA	NA	miRDB
CHD9	mRNA_set2	NA	NA	miRDB
CRMP1	mRNA_set2	NA	NA	miRDB
SPOPL	mRNA_set2	NA	NA	miRDB
VCPIP1	mRNA_set2	NA	NA	miRDB
GALNT4	mRNA_set2	NA	NA	miRDB
SNX16	mRNA_set2	NA	NA	miRDB
TLL1	mRNA_set2	NA	NA	miRDB
ANO3	mRNA_set2	NA	NA	miRDB
OMA1	mRNA_set2	NA	NA	miRDB
MYB	mRNA_set2	NA	NA	miRDB
CHD3	mRNA_set2	NA	NA	miRDB
GABRA4	mRNA_set2	NA	NA	miRDB
RWDD4	mRNA_set2	NA	NA	miRDB
POC1B-GALNT4	mRNA_set2	NA	NA	miRDB
COG6	mRNA_set2	NA	NA	miRDB
LZTS3	mRNA_set2	NA	NA	miRDB
FOXP2	mRNA_set2	NA	NA	miRDB
ATF3	mRNA_set2	NA	NA	miRDB
SLC46A3	mRNA_set2	NA	NA	miRDB

HINT3	mRNA_set2	NA	NA	miRDB
PLEKHM3	mRNA_set2	NA	NA	miRDB
CALU	mRNA_set2	NA	NA	miRDB
EPHA4	mRNA_set2	NA	NA	miRDB
DCHS2	mRNA_set2	NA	NA	miRDB
ZNF100	mRNA_set2	NA	NA	miRDB
POLR2F	mRNA_set2	NA	NA	miRDB
GABRR2	mRNA_set2	NA	NA	miRDB
MED13L	mRNA_set2	NA	NA	miRDB
ZNF189	mRNA_set2	NA	NA	miRDB
RETREG3	mRNA_set2	NA	NA	miRDB
VIP	mRNA_set2	NA	NA	miRDB
C5orf63	mRNA_set2	NA	NA	miRDB
BCOR	mRNA_set2	NA	NA	miRDB
FAM189B	mRNA_set2	NA	NA	miRDB
RS1	mRNA_set2	NA	NA	miRDB
ABCA12	mRNA_set2	NA	NA	miRDB
ALG8	mRNA_set2	NA	NA	miRDB
MSI2	mRNA_set2	NA	NA	miRDB
CHCHD3	mRNA_set2	NA	NA	miRDB
ZNF274	mRNA_set2	NA	NA	miRDB
EMP1	mRNA_set2	NA	NA	miRDB
DDHD2	mRNA_set2	NA	NA	miRDB
RASAL1	mRNA_set2	NA	NA	miRDB
EPB41L5	mRNA_set2	NA	NA	miRDB
NSD2	mRNA_set2	NA	NA	miRDB
TMEM52B	mRNA_set2	NA	NA	miRDB
C7orf71	mRNA_set2	NA	NA	miRDB

ZC3H12C	mRNA_set2	NA	NA	miRDB
CNTNAP2	mRNA_set2	NA	NA	miRDB
GABRA3	mRNA_set2	NA	NA	miRDB
GTF3C1	mRNA_set2	NA	NA	miRDB
PRDM5	mRNA_set2	NA	NA	miRDB
KLHL15	mRNA_set2	NA	NA	miRDB
NSF	mRNA_set2	NA	NA	miRDB
FHL5	mRNA_set2	NA	NA	miRDB
ATG16L1	mRNA_set2	NA	NA	miRDB
ETF1	mRNA_set2	NA	NA	miRDB
NFYC	mRNA_set2	NA	NA	miRDB
DHCR24	mRNA_set2	NA	NA	miRDB
COX15	mRNA_set2	NA	NA	miRDB
AFF1	mRNA_set2	NA	NA	miRDB
FGF11	mRNA_set2	NA	NA	miRDB
CEP70	mRNA_set2	NA	NA	miRDB
TRIM36	mRNA_set2	NA	NA	miRDB
CASC1	mRNA_set2	NA	NA	miRDB
OAZ1	mRNA_set2	NA	NA	miRDB
CWF19L2	mRNA_set2	NA	NA	miRDB
TMPRSS11B	mRNA_set2	NA	NA	miRDB
INPP4B	mRNA_set2	NA	NA	miRDB
SEMA3C	mRNA_set2	NA	NA	miRDB
HIVEP1	mRNA_set2	NA	NA	miRDB
FERMT3	mRNA_set2	NA	NA	miRDB
C5orf22	mRNA_set2	NA	NA	miRDB
PACSIN2	mRNA_set2	NA	NA	miRDB
PIP4K2C	mRNA_set2	NA	NA	miRDB

ARHGAP29	mRNA_set2	NA	NA	miRDB
SENP6	mRNA_set2	NA	NA	miRDB
GYPA	mRNA_set2	NA	NA	miRDB
ZNF609	mRNA_set2	NA	NA	miRDB
TMEM33	mRNA_set2	NA	NA	miRDB
TMF1	mRNA_set2	NA	NA	miRDB
TBC1D8B	mRNA_set2	NA	NA	miRDB
FBXW11	mRNA_set2	NA	NA	miRDB
COL13A1	mRNA_set2	NA	NA	miRDB
CAMK2D	mRNA_set2	NA	NA	miRDB
SNTG2	mRNA_set2	NA	NA	miRDB
PRXL2C	mRNA_set2	NA	NA	miRDB
LRIG3	mRNA_set2	NA	NA	miRDB
MIS18BP1	mRNA_set2	NA	NA	miRDB
TRAPPC6B	mRNA_set2	NA	NA	miRDB
SOX6	mRNA_set2	NA	NA	miRDB
CHRNA3	mRNA_set2	NA	NA	miRDB
ZFP3	mRNA_set2	NA	NA	miRDB
MCEE	mRNA_set2	NA	NA	miRDB
IFITM10	mRNA_set2	NA	NA	miRDB
WDR43	mRNA_set2	NA	NA	miRDB
NUP153	mRNA_set2	NA	NA	miRDB
NLK	mRNA_set2	NA	NA	miRDB
DNA2	mRNA_set2	NA	NA	miRDB
SMARCA1	mRNA_set2	NA	NA	miRDB
JMJD8	mRNA_set2	NA	NA	miRDB
DEPDC1B	mRNA_set2	NA	NA	miRDB
SREK1	mRNA_set2	NA	NA	miRDB

SLC18B1	mRNA_set2	NA	NA	miRDB
ATP5MC3	mRNA_set2	NA	NA	miRDB
ARNT2	mRNA_set2	NA	NA	miRDB
ADGRG6	mRNA_set2	NA	NA	miRDB
EHD3	mRNA_set2	NA	NA	miRDB
BCAN	mRNA_set2	NA	NA	miRDB
PRR14L	mRNA_set2	NA	NA	miRDB
TARDBP	mRNA_set2	NA	NA	miRDB
PLPP6	mRNA_set2	NA	NA	miRDB
NRXN1	mRNA_set2	NA	NA	miRDB
CBX1	mRNA_set2	NA	NA	miRDB
BRSK2	mRNA_set2	NA	NA	miRDB
DNAJC12	mRNA_set2	NA	NA	miRDB
YPEL1	mRNA_set2	NA	NA	miRDB
B4GALNT4	mRNA_set2	NA	NA	miRDB
TFPI	mRNA_set2	NA	NA	miRDB
PDPN	mRNA_set2	NA	NA	miRDB
ZNF124	mRNA_set2	NA	NA	miRDB
MXI1	mRNA_set2	NA	NA	miRDB
PLAGL2	mRNA_set2	NA	NA	miRDB
LSM11	mRNA_set2	NA	NA	miRDB
CYFIP2	mRNA_set2	NA	NA	miRDB
MLH1	mRNA_set2	NA	NA	miRDB
AK2	mRNA_set2	NA	NA	miRDB
2-Mar	mRNA_set2	NA	NA	miRDB
EPB41L3	mRNA_set2	NA	NA	miRDB
SNRK	mRNA_set2	NA	NA	miRDB
DNAJB4	mRNA_set2	NA	NA	miRDB

BCL7B	mRNA_set2	NA	NA	miRDB
RBMS2	mRNA_set2	NA	NA	miRDB
SPTBN1	mRNA_set2	NA	NA	miRDB
KIAA1841	mRNA_set2	NA	NA	miRDB
VGLL4	mRNA_set2	NA	NA	miRDB
JPH2	mRNA_set2	NA	NA	miRDB
OLFML2A	mRNA_set2	NA	NA	miRDB
LPP	mRNA_set2	NA	NA	miRDB
TM4SF1	mRNA_set2	NA	NA	miRDB
ARHGAP18	mRNA_set2	NA	NA	miRDB
PDGFA	mRNA_set2	NA	NA	miRDB
CARMIL1	mRNA_set2	NA	NA	miRDB
SLC44A2	mRNA_set2	NA	NA	miRDB
IQSEC3	mRNA_set2	NA	NA	miRDB
SLC31A1	mRNA_set2	NA	NA	miRDB
P2RY2	mRNA_set2	NA	NA	miRDB
BLCAP	mRNA_set2	NA	NA	miRDB
GPR161	mRNA_set2	NA	NA	miRDB
NUP58	mRNA_set2	NA	NA	miRDB
RERG	mRNA_set2	NA	NA	miRDB
COA5	mRNA_set2	NA	NA	miRDB
AKR1C2	mRNA_set2	NA	NA	miRDB
HPSE2	mRNA_set2	NA	NA	miRDB
CHORDC1	mRNA_set2	NA	NA	miRDB
CSRNP3	mRNA_set2	NA	NA	miRDB
ZFP91	mRNA_set2	NA	NA	miRDB
RSU1	mRNA_set2	NA	NA	miRDB
OIT3	mRNA_set2	NA	NA	miRDB

COMMD2	mRNA_set2	NA	NA	miRDB
POC5	mRNA_set2	NA	NA	miRDB
PDZRN4	mRNA_set2	NA	NA	miRDB
KRT6A	mRNA_set2	NA	NA	miRDB
PHEX	mRNA_set2	NA	NA	miRDB
LGR4	mRNA_set2	NA	NA	miRDB
RSF1	mRNA_set2	NA	NA	miRDB
PUM2	mRNA_set2	NA	NA	miRDB
TRRAP	mRNA_set2	NA	NA	miRDB
NRG3	mRNA_set2	NA	NA	miRDB
NDUFS2	mRNA_set2	NA	NA	miRDB
MEF2A	mRNA_set2	NA	NA	miRDB
SUB1	mRNA_set2	NA	NA	miRDB
MAGEF1	mRNA_set2	NA	NA	miRDB
UMAD1	mRNA_set2	NA	NA	miRDB
GRAMD1C	mRNA_set2	NA	NA	miRDB
THOC1	mRNA_set2	NA	NA	miRDB
CHML	mRNA_set2	NA	NA	miRDB
CSNK1G1	mRNA_set2	NA	NA	miRDB
RNF144A	mRNA_set2	NA	NA	miRDB
OSBPL10	mRNA_set2	NA	NA	miRDB
RFX3	mRNA_set2	NA	NA	miRDB
AKR7A2	mRNA_set2	NA	NA	miRDB
MTSS1L	mRNA_set2	NA	NA	miRDB
ADD1	mRNA_set2	NA	NA	miRDB
CABP1	mRNA_set2	NA	NA	miRDB
PTGER2	mRNA_set2	NA	NA	miRDB
AGPS	mRNA_set2	NA	NA	miRDB

LYPD3	mRNA_set2	NA	NA	miRDB
RCAN1	mRNA_set2	NA	NA	miRDB
MMP16	mRNA_set2	NA	NA	miRDB
SRRM2	mRNA_set2	NA	NA	miRDB
GSE1	mRNA_set2	NA	NA	miRDB
HCRTR1	mRNA_set2	NA	NA	miRDB
ZBTB25	mRNA_set2	NA	NA	miRDB
CAP2	mRNA_set2	NA	NA	miRDB
FBXO46	mRNA_set2	NA	NA	miRDB
ZNF217	mRNA_set2	NA	NA	miRDB
MTMR3	mRNA_set2	NA	NA	miRDB
SGMS2	mRNA_set2	NA	NA	miRDB
C2orf80	mRNA_set2	NA	NA	miRDB
PI4KA	mRNA_set2	NA	NA	miRDB
DNAJB12	mRNA_set2	NA	NA	miRDB
SACS	mRNA_set2	NA	NA	miRDB
KIAA1324L	mRNA_set2	NA	NA	miRDB
POTEF	mRNA_set2	NA	NA	miRDB
PFN2	mRNA_set2	NA	NA	miRDB
GNB1	mRNA_set2	NA	NA	miRDB
RNF19A	mRNA_set2	NA	NA	miRDB
WASF2	mRNA_set2	NA	NA	miRDB
MR1	mRNA_set2	NA	NA	miRDB
MAPK1	mRNA_set2	NA	NA	miRDB
COIL	mRNA_set2	NA	NA	miRDB
PLEKHS1	mRNA_set2	NA	NA	miRDB
STX6	mRNA_set2	NA	NA	miRDB
C9orf152	mRNA_set2	NA	NA	miRDB

ZDHHC15	mRNA_set2	NA	NA	miRDB
LRRK1	mRNA_set2	NA	NA	miRDB
MXD4	mRNA_set2	NA	NA	miRDB
SMAP1	mRNA_set2	NA	NA	miRDB
DAP	mRNA_set2	NA	NA	miRDB
NOTCH2	mRNA_set2	NA	NA	miRDB
MAP3K3	mRNA_set2	NA	NA	miRDB
FAM57A	mRNA_set2	NA	NA	miRDB
UNC50	mRNA_set2	NA	NA	miRDB
PPM1E	mRNA_set2	NA	NA	miRDB
PMEPA1	mRNA_set2	NA	NA	miRDB
CBLN1	mRNA_set2	NA	NA	miRDB
TRIM71	mRNA_set2	NA	NA	miRDB
CLIC4	mRNA_set2	NA	NA	miRDB
HIF1A	mRNA_set2	NA	NA	miRDB
CTDSPL2	mRNA_set2	NA	NA	miRDB
BBOF1	mRNA_set2	NA	NA	miRDB
SHPRH	mRNA_set2	NA	NA	miRDB
ZNF28	mRNA_set2	NA	NA	miRDB
LARP1B	mRNA_set2	NA	NA	miRDB
TIMP2	mRNA_set2	NA	NA	miRDB
ATAD1	mRNA_set2	NA	NA	miRDB
UNC80	mRNA_set2	NA	NA	miRDB
SMIM13	mRNA_set2	NA	NA	miRDB
ANKS1B	mRNA_set2	NA	NA	miRDB
CNPY1	mRNA_set2	NA	NA	miRDB
CHERP	mRNA_set2	NA	NA	miRDB
BICRA	mRNA_set2	NA	NA	miRDB

WAC	mRNA_set2	NA	NA	miRDB
ZNF571	mRNA_set2	NA	NA	miRDB
ANOS1	mRNA_set2	NA	NA	miRDB
ZBTB11	mRNA_set2	NA	NA	miRDB
LIN7A	mRNA_set2	NA	NA	miRDB
TNFRSF11B	mRNA_set2	NA	NA	miRDB
NKX6-2	mRNA_set2	NA	NA	miRDB
IGF2R	mRNA_set2	NA	NA	miRDB
PDE4D	mRNA_set2	NA	NA	miRDB
XCL2	mRNA_set2	NA	NA	miRDB
LRCH4	mRNA_set2	NA	NA	miRDB
FAR1	mRNA_set2	NA	NA	miRDB
CELF1	mRNA_set2	NA	NA	miRDB
PCF11	mRNA_set2	NA	NA	miRDB
RNF4	mRNA_set2	NA	NA	miRDB
FRZB	mRNA_set2	NA	NA	miRDB
KCNC2	mRNA_set2	NA	NA	miRDB
GRSF1	mRNA_set2	NA	NA	miRDB
RTKN2	mRNA_set2	NA	NA	miRDB
GNB4	mRNA_set2	NA	NA	miRDB
DAZAP2	mRNA_set2	NA	NA	miRDB
URB1	mRNA_set2	NA	NA	miRDB
ADAMTS5	mRNA_set2	NA	NA	miRDB
SFXN2	mRNA_set2	NA	NA	miRDB
POM121C	mRNA_set2	NA	NA	miRDB
GCA	mRNA_set2	NA	NA	miRDB
CCDC175	mRNA_set2	NA	NA	miRDB
GSPT2	mRNA_set2	NA	NA	miRDB

TCEA1	mRNA_set2	NA	NA	miRDB
GRIN3A	mRNA_set2	NA	NA	miRDB
C6orf120	mRNA_set2	NA	NA	miRDB
CHST2	mRNA_set2	NA	NA	miRDB
NME9	mRNA_set2	NA	NA	miRDB
MRPS25	mRNA_set2	NA	NA	miRDB
MAGI1	mRNA_set2	NA	NA	miRDB
SLF2	mRNA_set2	NA	NA	miRDB
MOB1B	mRNA_set2	NA	NA	miRDB
CYREN	mRNA_set2	NA	NA	miRDB
OXSR1	mRNA_set2	NA	NA	miRDB
TIMM23	mRNA_set2	NA	NA	miRDB
CPD	mRNA_set2	NA	NA	miRDB
TUBE1	mRNA_set2	NA	NA	miRDB
GTPBP10	mRNA_set2	NA	NA	miRDB
HIF1AN	mRNA_set2	NA	NA	miRDB
KRT85	mRNA_set2	NA	NA	miRDB
LAMB2	mRNA_set2	NA	NA	miRDB
RNASEL	mRNA_set2	NA	NA	miRDB
STXBP5L	mRNA_set2	NA	NA	miRDB
BTD	mRNA_set2	NA	NA	miRDB
PALLD	mRNA_set2	NA	NA	miRDB
DKK1	mRNA_set2	NA	NA	miRDB
FYB1	mRNA_set2	NA	NA	miRDB
PIEZO2	mRNA_set2	NA	NA	miRDB
SLC25A19	mRNA_set2	NA	NA	miRDB
GNE	mRNA_set2	NA	NA	miRDB
OAS2	mRNA_set2	NA	NA	miRDB

CPLX2	mRNA_set2	NA	NA	miRDB
ZNF264	mRNA_set2	NA	NA	miRDB
ZNF280C	mRNA_set2	NA	NA	miRDB
CASK	mRNA_set2	NA	NA	miRDB
CCND2	mRNA_set2	NA	NA	miRDB
WEE1	mRNA_set2	NA	NA	miRDB
SH3GL1	mRNA_set2	NA	NA	miRDB
DHX15	mRNA_set2	NA	NA	miRDB
GRM1	mRNA_set2	NA	NA	miRDB
SETD9	mRNA_set2	NA	NA	miRDB
ELF3	mRNA_set2	NA	NA	miRDB
PDLIM4	mRNA_set2	NA	NA	miRDB
SMCO1	mRNA_set2	NA	NA	miRDB
OPHN1	mRNA_set2	NA	NA	miRDB
NAV1	mRNA_set2	NA	NA	miRDB
NLN	mRNA_set2	NA	NA	miRDB
METRNL	mRNA_set2	NA	NA	miRDB
RHOB	mRNA_set2	NA	NA	miRDB
CLDN12	mRNA_set2	NA	NA	miRDB
ZNF391	mRNA_set2	NA	NA	miRDB
TMEM59	mRNA_set2	NA	NA	miRDB
C9orf72	mRNA_set2	NA	NA	miRDB
MRPS10	mRNA_set2	NA	NA	miRDB
KIDINS220	mRNA_set2	NA	NA	miRDB
TOM1L2	mRNA_set2	NA	NA	miRDB
MDGA2	mRNA_set2	NA	NA	miRDB
ADGRL3	mRNA_set2	NA	NA	miRDB
CDH7	mRNA_set2	NA	NA	miRDB

GLIPR1	mRNA_set2	NA	NA	miRDB
TMEM232	mRNA_set2	NA	NA	miRDB
RDX	mRNA_set2	NA	NA	miRDB
ACOX1	mRNA_set2	NA	NA	miRDB
GABRG1	mRNA_set2	NA	NA	miRDB
WDR33	mRNA_set2	NA	NA	miRDB
JAKMIP3	mRNA_set2	NA	NA	miRDB
PTS	mRNA_set2	NA	NA	miRDB
ENC1	mRNA_set2	NA	NA	miRDB
CCNF	mRNA_set2	NA	NA	miRDB
DIP2A	mRNA_set2	NA	NA	miRDB
BEND7	mRNA_set2	NA	NA	miRDB
LRP6	mRNA_set2	NA	NA	miRDB
RNF125	mRNA_set2	NA	NA	miRDB
FZD6	mRNA_set2	NA	NA	miRDB
CTSS	mRNA_set2	NA	NA	miRDB
DERL1	mRNA_set2	NA	NA	miRDB
YIPF5	mRNA_set2	NA	NA	miRDB
CTTN	mRNA_set2	NA	NA	miRDB
ERLIN1	mRNA_set2	NA	NA	miRDB
CFAP300	mRNA_set2	NA	NA	miRDB
HELZ	mRNA_set2	NA	NA	miRDB
C7orf33	mRNA_set2	NA	NA	miRDB
ZNF705A	mRNA_set2	NA	NA	miRDB
SLC1A2	mRNA_set2	NA	NA	miRDB
CYP1B1	mRNA_set2	NA	NA	miRDB
FASTKD5	mRNA_set2	NA	NA	miRDB
CRTC1	mRNA_set2	NA	NA	miRDB

EDIL3	mRNA_set2	NA	NA	miRDB
SCN2A	mRNA_set2	NA	NA	miRDB
PLD6	mRNA_set2	NA	NA	miRDB
KRTAP13-2	mRNA_set2	NA	NA	miRDB
CDC25B	mRNA_set2	NA	NA	miRDB
SEMA4C	mRNA_set2	NA	NA	miRDB
HRASLS5	mRNA_set2	NA	NA	miRDB
GSK3B	mRNA_set2	NA	NA	miRDB
VPS26B	mRNA_set2	NA	NA	miRDB
KLHDC3	mRNA_set2	NA	NA	miRDB
HCN4	mRNA_set2	NA	NA	miRDB
GOSR2	mRNA_set2	NA	NA	miRDB
DPYD	mRNA_set2	NA	NA	miRDB
TRABD2B	mRNA_set2	NA	NA	miRDB
JDP2	mRNA_set2	NA	NA	miRDB
DLGAP3	mRNA_set2	NA	NA	miRDB
PRR16	mRNA_set2	NA	NA	miRDB
SYNPO2	mRNA_set2	NA	NA	miRDB
NFKBID	mRNA_set2	NA	NA	miRDB
HMGA2	mRNA_set2	NA	NA	miRDB
ZNF445	mRNA_set2	NA	NA	miRDB
NUP133	mRNA_set2	NA	NA	miRDB
ZNF736	mRNA_set2	NA	NA	miRDB
UBXN1	mRNA_set2	NA	NA	miRDB
RUFY2	mRNA_set2	NA	NA	miRDB
NUCKS1	mRNA_set2	NA	NA	miRDB
TMEM187	mRNA_set2	NA	NA	miRDB
IL6ST	mRNA_set2	NA	NA	miRDB

NOVA2	mRNA_set2	NA	NA	miRDB
DST	mRNA_set2	NA	NA	miRDB
CLN5	mRNA_set2	NA	NA	miRDB
C3orf80	mRNA_set2	NA	NA	miRDB
IKBKB	mRNA_set2	NA	NA	miRDB
GLUL	mRNA_set2	NA	NA	miRDB
TKT	mRNA_set2	NA	NA	miRDB
KNOP1	mRNA_set2	NA	NA	miRDB
H3F3C	mRNA_set2	NA	NA	miRDB
PPM1H	mRNA_set2	NA	NA	miRDB
ABCC5	mRNA_set2	NA	NA	miRDB
DBN1	mRNA_set2	NA	NA	miRDB
PDF	mRNA_set2	NA	NA	miRDB
MICAL3	mRNA_set2	NA	NA	miRDB
ACVR2B	mRNA_set2	NA	NA	miRDB
BICD2	mRNA_set2	NA	NA	miRDB
LRAT	mRNA_set2	NA	NA	miRDB
BCL2L2	mRNA_set2	NA	NA	miRDB
DIAPH1	mRNA_set2	NA	NA	miRDB
LRRC46	mRNA_set2	NA	NA	miRDB
TLR5	mRNA_set2	NA	NA	miRDB
ZNF827	mRNA_set2	NA	NA	miRDB
FAM122B	mRNA_set2	NA	NA	miRDB
ZNF561	mRNA_set2	NA	NA	miRDB
GRIN2A	mRNA_set2	NA	NA	miRDB
SRSF10	mRNA_set2	NA	NA	miRDB
ZNF117	mRNA_set2	NA	NA	miRDB
SPATA17	mRNA_set2	NA	NA	miRDB

SEPHS1	mRNA_set2	NA	NA	miRDB
FAM204A	mRNA_set2	NA	NA	miRDB
PPM1B	mRNA_set2	NA	NA	miRDB
ATG5	mRNA_set2	NA	NA	miRDB
LTN1	mRNA_set2	NA	NA	miRDB
IMPG2	mRNA_set2	NA	NA	miRDB
NRG1	mRNA_set2	NA	NA	miRDB
SORT1	mRNA_set2	NA	NA	miRDB
EMC4	mRNA_set2	NA	NA	miRDB
FGFR4	mRNA_set2	NA	NA	miRDB
PRELID3B	mRNA_set2	NA	NA	miRDB
SRGN	mRNA_set2	NA	NA	miRDB
VPS4B	mRNA_set2	NA	NA	miRDB
PEX5L	mRNA_set2	NA	NA	miRDB
ITCH	mRNA_set2	NA	NA	miRDB
ETNK1	mRNA_set2	NA	NA	miRDB
RANBP3L	mRNA_set2	NA	NA	miRDB
TMED4	mRNA_set2	NA	NA	miRDB
STARD3NL	mRNA_set2	NA	NA	miRDB
NAA25	mRNA_set2	NA	NA	miRDB
SELENON	mRNA_set2	NA	NA	miRDB
PAK6	mRNA_set2	NA	NA	miRDB
ELL2	mRNA_set2	NA	NA	miRDB
SENP1	mRNA_set2	NA	NA	miRDB
CSNK1D	mRNA_set2	NA	NA	miRDB
GTF2I	mRNA_set2	NA	NA	miRDB
SMIM17	mRNA_set2	NA	NA	miRDB
YPEL3	mRNA_set2	NA	NA	miRDB

C14orf93	mRNA_set2	NA	NA	miRDB
ASB7	mRNA_set2	NA	NA	miRDB
FAM200B	mRNA_set2	NA	NA	miRDB
ZNF81	mRNA_set2	NA	NA	miRDB
LONRF2	mRNA_set2	NA	NA	miRDB
ARID2	mRNA_set2	NA	NA	miRDB
GRP	mRNA_set2	NA	NA	miRDB
SLC5A3	mRNA_set2	NA	NA	miRDB
RFC3	mRNA_set2	NA	NA	miRDB
RBMXL1	mRNA_set2	NA	NA	miRDB
GLYATL1	mRNA_set2	NA	NA	miRDB
WDR35	mRNA_set2	NA	NA	miRDB
GALNT12	mRNA_set2	NA	NA	miRDB
TDP1	mRNA_set2	NA	NA	miRDB
MAGIX	mRNA_set2	NA	NA	miRDB
STAP1	mRNA_set2	NA	NA	miRDB
CNIH4	mRNA_set2	NA	NA	miRDB
KCNH1	mRNA_set2	NA	NA	miRDB
RUNX1T1	mRNA_set2	NA	NA	miRDB
PRDM15	mRNA_set2	NA	NA	miRDB
MRPL37	mRNA_set2	NA	NA	miRDB
6-Sep	mRNA_set2	NA	NA	miRDB
PPP1R3B	mRNA_set2	NA	NA	miRDB
TRANK1	mRNA_set2	NA	NA	miRDB
NME1-NME2	mRNA_set2	NA	NA	miRDB
FER	mRNA_set2	NA	NA	miRDB
PASK	mRNA_set2	NA	NA	miRDB
SH3PXD2B	mRNA_set2	NA	NA	miRDB

SLC30A5	mRNA_set2	NA	NA	miRDB
RRBP1	mRNA_set2	NA	NA	miRDB
SPTLC3	mRNA_set2	NA	NA	miRDB
AMOT	mRNA_set2	NA	NA	miRDB
PABPC1L2A	mRNA_set2	NA	NA	miRDB
AOAH	mRNA_set2	NA	NA	miRDB
DBT	mRNA_set2	NA	NA	miRDB
ELK1	mRNA_set2	NA	NA	miRDB
AGAP9	mRNA_set2	NA	NA	miRDB
LCE3E	mRNA_set2	NA	NA	miRDB
DDX11	mRNA_set2	NA	NA	miRDB
MIEF1	mRNA_set2	NA	NA	miRDB
AGAP3	mRNA_set2	NA	NA	miRDB
DYNLRB2	mRNA_set2	NA	NA	miRDB
DNAJC11	mRNA_set2	NA	NA	miRDB
CALN1	mRNA_set2	NA	NA	miRDB
SSH2	mRNA_set2	NA	NA	miRDB
RAPGEF6	mRNA_set2	NA	NA	miRDB
CCDC89	mRNA_set2	NA	NA	miRDB
NIT2	mRNA_set2	NA	NA	miRDB
ITGA10	mRNA_set2	NA	NA	miRDB
DNAJC15	mRNA_set2	NA	NA	miRDB
HIKESHI	mRNA_set2	NA	NA	miRDB
OPRM1	mRNA_set2	NA	NA	miRDB
MIB1	mRNA_set2	NA	NA	miRDB
UNC5C	mRNA_set2	NA	NA	miRDB
RAI14	mRNA_set2	NA	NA	miRDB
SLITRK6	mRNA_set2	NA	NA	miRDB

GABRA1	mRNA_set2	NA	NA	miRDB
CDC37L1	mRNA_set2	NA	NA	miRDB
ABCC9	mRNA_set2	NA	NA	miRDB
CCT5	mRNA_set2	NA	NA	miRDB
CA7	mRNA_set2	NA	NA	miRDB
TNKS	mRNA_set2	NA	NA	miRDB
WDR44	mRNA_set2	NA	NA	miRDB
ATP13A3	mRNA_set2	NA	NA	miRDB
EEPD1	mRNA_set2	NA	NA	miRDB
ACACA	mRNA_set2	NA	NA	miRDB
CTCF	mRNA_set2	NA	NA	miRDB
ANKRD17	mRNA_set2	NA	NA	miRDB
ARID5B	mRNA_set2	NA	NA	miRDB
BCL3	mRNA_set2	NA	NA	miRDB
PRDM11	mRNA_set2	NA	NA	miRDB
E2F6	mRNA_set2	NA	NA	miRDB
CASC3	mRNA_set2	NA	NA	miRDB
KCTD4	mRNA_set2	NA	NA	miRDB
IGLON5	mRNA_set2	NA	NA	miRDB
ZNF783	mRNA_set2	NA	NA	miRDB
SUMO2	mRNA_set2	NA	NA	miRDB
SHB	mRNA_set2	NA	NA	miRDB
VASH2	mRNA_set2	NA	NA	miRDB
FBXL20	mRNA_set2	NA	NA	miRDB
EPG5	mRNA_set2	NA	NA	miRDB
SIGLEC8	mRNA_set2	NA	NA	miRDB
UGT8	mRNA_set2	NA	NA	miRDB
ISM2	mRNA_set2	NA	NA	miRDB

HSPA4L	mRNA_set2	NA	NA	miRDB
EPDR1	mRNA_set2	NA	NA	miRDB
CCDC85A	mRNA_set2	NA	NA	miRDB
FLT1	mRNA_set2	NA	NA	miRDB
BHLHE41	mRNA_set2	NA	NA	miRDB
IPCEF1	mRNA_set2	NA	NA	miRDB
TGFBRAP1	mRNA_set2	NA	NA	miRDB
ZNF605	mRNA_set2	NA	NA	miRDB
C16orf91	mRNA_set2	NA	NA	miRDB
MPLKIP	mRNA_set2	NA	NA	miRDB
BET1	mRNA_set2	NA	NA	miRDB
TEX35	mRNA_set2	NA	NA	miRDB
LHX9	mRNA_set2	NA	NA	miRDB
YPEL2	mRNA_set2	NA	NA	miRDB
PARVA	mRNA_set2	NA	NA	miRDB
GAS7	mRNA_set2	NA	NA	miRDB
GAK	mRNA_set2	NA	NA	miRDB
DENND1B	mRNA_set2	NA	NA	miRDB
UBN1	mRNA_set2	NA	NA	miRDB
GALNT16	mRNA_set2	NA	NA	miRDB
SOX3	mRNA_set2	NA	NA	miRDB
SAXO1	mRNA_set2	NA	NA	miRDB
PAPPA	mRNA_set2	NA	NA	miRDB
SH3BP5L	mRNA_set2	NA	NA	miRDB
ZNF618	mRNA_set2	NA	NA	miRDB
TRIM26	mRNA_set2	NA	NA	miRDB
RBMX	mRNA_set2	NA	NA	miRDB
SERTAD4	mRNA_set2	NA	NA	miRDB

SUSD6	mRNA_set2	NA	NA	miRDB
UBTF	mRNA_set2	NA	NA	miRDB
SLC37A2	mRNA_set2	NA	NA	miRDB
AGAP4	mRNA_set2	NA	NA	miRDB
AAK1	mRNA_set2	NA	NA	miRDB
MYD88	mRNA_set2	NA	NA	miRDB
ARHGEF19	mRNA_set2	NA	NA	miRDB
MFAP5	mRNA_set2	NA	NA	miRDB
USP14	mRNA_set2	NA	NA	miRDB
KMO	mRNA_set2	NA	NA	miRDB
SH3KBP1	mRNA_set2	NA	NA	miRDB
NMBR	mRNA_set2	NA	NA	miRDB
LOC100132813	mRNA_set2	NA	NA	miRDB
GUCY1A1	mRNA_set2	NA	NA	miRDB
RFC5	mRNA_set2	NA	NA	miRDB
ZFC3H1	mRNA_set2	NA	NA	miRDB
PHC3	mRNA_set2	NA	NA	miRDB
RPL22	mRNA_set2	NA	NA	miRDB
IL2	mRNA_set2	NA	NA	miRDB
ASCC3	mRNA_set2	NA	NA	miRDB
NAIF1	mRNA_set2	NA	NA	miRDB
NCKAP1	mRNA_set2	NA	NA	miRDB
PJVK	mRNA_set2	NA	NA	miRDB
IL31	mRNA_set2	NA	NA	miRDB
CCDC82	mRNA_set2	NA	NA	miRDB
KSR2	mRNA_set2	NA	NA	miRDB
CADM2	mRNA_set2	NA	NA	miRDB
MAP2	mRNA_set2	NA	NA	miRDB

UXS1	mRNA_set2	NA	NA	miRDB
ERLEC1	mRNA_set2	NA	NA	miRDB
HDC	mRNA_set2	NA	NA	miRDB
ZNF594	mRNA_set2	NA	NA	miRDB
TMEM167B	mRNA_set2	NA	NA	miRDB
TOX	mRNA_set2	NA	NA	miRDB
RIMKLB	mRNA_set2	NA	NA	miRDB
SLC14A1	mRNA_set2	NA	NA	miRDB
SAP30	mRNA_set2	NA	NA	miRDB
ANO5	mRNA_set2	NA	NA	miRDB
DIAPH3	mRNA_set2	NA	NA	miRDB
ERP44	mRNA_set2	NA	NA	miRDB
TPBG	mRNA_set2	NA	NA	miRDB
LRRIQ1	mRNA_set2	NA	NA	miRDB
GABRB3	mRNA_set2	NA	NA	miRDB
LUZP2	mRNA_set2	NA	NA	miRDB
HNRNPR	mRNA_set2	NA	NA	miRDB
PPFIA1	mRNA_set2	NA	NA	miRDB
ABHD3	mRNA_set2	NA	NA	miRDB
ADIPOR2	mRNA_set2	NA	NA	miRDB
MAPK10	mRNA_set2	NA	NA	miRDB
PREPL	mRNA_set2	NA	NA	miRDB
PPM1K	mRNA_set2	NA	NA	miRDB
CHMP2B	mRNA_set2	NA	NA	miRDB
NCKAP5	mRNA_set2	NA	NA	miRDB
N4BP2L1	mRNA_set2	NA	NA	miRDB
CLEC1A	mRNA_set2	NA	NA	miRDB
LHX3	mRNA_set2	NA	NA	miRDB

NFIB	mRNA_set2	NA	NA	miRDB
LPIN2	mRNA_set2	NA	NA	miRDB
SLC12A2	mRNA_set2	NA	NA	miRDB
UBE2F	mRNA_set2	NA	NA	miRDB
C18orf65	mRNA_set2	NA	NA	miRDB
TTC39B	mRNA_set2	NA	NA	miRDB
ARSJ	mRNA_set2	NA	NA	miRDB
ADD3	mRNA_set2	NA	NA	miRDB
CDS2	mRNA_set2	NA	NA	miRDB
EMCN	mRNA_set2	NA	NA	miRDB
TET3	mRNA_set2	NA	NA	miRDB
MAN2A1	mRNA_set2	NA	NA	miRDB
CDYL	mRNA_set2	NA	NA	miRDB
IQCH	mRNA_set2	NA	NA	miRDB
HLA-DRA	mRNA_set2	NA	NA	miRDB
UTP23	mRNA_set2	NA	NA	miRDB
FRY	mRNA_set2	NA	NA	miRDB
PHF24	mRNA_set2	NA	NA	miRDB
SBNO1	mRNA_set2	NA	NA	miRDB
UHRF1	mRNA_set2	NA	NA	miRDB
NCOR1	mRNA_set2	NA	NA	miRDB
ATP2A2	mRNA_set2	NA	NA	miRDB
RAB9B	mRNA_set2	NA	NA	miRDB
WDR19	mRNA_set2	NA	NA	miRDB
PARPBP	mRNA_set2	NA	NA	miRDB
PSIP1	mRNA_set2	NA	NA	miRDB
NME2	mRNA_set2	NA	NA	miRDB
C6orf89	mRNA_set2	NA	NA	miRDB

WASL	mRNA_set2	NA	NA	miRDB
STUM	mRNA_set2	NA	NA	miRDB
RAB43	mRNA_set2	NA	NA	miRDB
CTBP2	mRNA_set2	NA	NA	miRDB
MTSS1	mRNA_set2	NA	NA	miRDB
LMBR1	mRNA_set2	NA	NA	miRDB
WNT3	mRNA_set2	NA	NA	miRDB
ELOVL2	mRNA_set2	NA	NA	miRDB
GEN1	mRNA_set2	NA	NA	miRDB
HNRNPA1L2	mRNA_set2	NA	NA	miRDB
ASH1L	mRNA_set2	NA	NA	miRDB
FRAS1	mRNA_set2	NA	NA	miRDB
PLEKHO2	mRNA_set2	NA	NA	miRDB
SLC30A9	mRNA_set2	NA	NA	miRDB
VAMP2	mRNA_set2	NA	NA	miRDB
ZNF516	mRNA_set2	NA	NA	miRDB
IFI44L	mRNA_set2	NA	NA	miRDB
COL5A1	mRNA_set2	NA	NA	miRDB
IQSEC2	mRNA_set2	NA	NA	miRDB
AGAP5	mRNA_set2	NA	NA	miRDB
ARHGEF25	mRNA_set2	NA	NA	miRDB
SRARP	mRNA_set2	NA	NA	miRDB
ZCRB1	mRNA_set2	NA	NA	miRDB
LEP	mRNA_set2	NA	NA	miRDB
ZNF550	mRNA_set2	NA	NA	miRDB
TSBP1	mRNA_set2	NA	NA	miRDB
SPRED2	mRNA_set2	NA	NA	miRDB
ZNF791	mRNA_set2	NA	NA	miRDB

KRTAP9-9	mRNA_set2	NA	NA	miRDB
RIMBP2	mRNA_set2	NA	NA	miRDB
GIN1	mRNA_set2	NA	NA	miRDB
PDCD6IP	mRNA_set2	NA	NA	miRDB
HFE	mRNA_set2	NA	NA	miRDB
ACTR2	mRNA_set2	NA	NA	miRDB
CUL4A	mRNA_set2	NA	NA	miRDB
SLC27A2	mRNA_set2	NA	NA	miRDB
SPAG9	mRNA_set2	NA	NA	miRDB
SOX30	mRNA_set2	NA	NA	miRDB
PEX19	mRNA_set2	NA	NA	miRDB
KPNA1	mRNA_set2	NA	NA	miRDB
LATS1	mRNA_set2	NA	NA	miRDB
G2E3	mRNA_set2	NA	NA	miRDB
MAFK	mRNA_set2	NA	NA	miRDB
TMEM106B	mRNA_set2	NA	NA	miRDB
APC	mRNA_set2	NA	NA	miRDB
HS6ST2	mRNA_set2	NA	NA	miRDB
FAM177A1	mRNA_set2	NA	NA	miRDB
PTH2R	mRNA_set2	NA	NA	miRDB
PPHLN1	mRNA_set2	NA	NA	miRDB
HNRNPF	mRNA_set2	NA	NA	miRDB
ALG10	mRNA_set2	NA	NA	miRDB
SV2C	mRNA_set2	NA	NA	miRDB
STIMATE	mRNA_set2	NA	NA	miRDB
FSIP1	mRNA_set2	NA	NA	miRDB
RGPD3	mRNA_set2	NA	NA	miRDB
CEP85L	mRNA_set2	NA	NA	miRDB

RPN1	mRNA_set2	NA	NA	miRDB
ZNF106	mRNA_set2	NA	NA	miRDB
RGS17	mRNA_set2	NA	NA	miRDB
ANKRD36C	mRNA_set2	NA	NA	miRDB
PAQR5	mRNA_set2	NA	NA	miRDB
ARL13B	mRNA_set2	NA	NA	miRDB
HGF	mRNA_set2	NA	NA	miRDB
TMEM126B	mRNA_set2	NA	NA	miRDB
SNX30	mRNA_set2	NA	NA	miRDB
RAD54L2	mRNA_set2	NA	NA	miRDB
GYS1	mRNA_set2	NA	NA	miRDB
PM20D1	mRNA_set2	NA	NA	miRDB
ARL4D	mRNA_set2	NA	NA	miRDB
SYS1	mRNA_set2	NA	NA	miRDB
SFXN4	mRNA_set2	NA	NA	miRDB
MAGEC1	mRNA_set2	NA	NA	miRDB
RXYLT1	mRNA_set2	NA	NA	miRDB
KLC2	mRNA_set2	NA	NA	miRDB
TRAK1	mRNA_set2	NA	NA	miRDB
RXFP1	mRNA_set2	NA	NA	miRDB
PWWP3B	mRNA_set2	NA	NA	miRDB
WAPL	mRNA_set2	NA	NA	miRDB
NR2E1	mRNA_set2	NA	NA	miRDB
CNOT6	mRNA_set2	NA	NA	miRDB
SLC22A5	mRNA_set2	NA	NA	miRDB
MLEC	mRNA_set2	NA	NA	miRDB
TRIM45	mRNA_set2	NA	NA	miRDB
BCCIP	mRNA_set2	NA	NA	miRDB

NECTIN3	mRNA_set2	NA	NA	miRDB
STEAP4	mRNA_set2	NA	NA	miRDB
C14orf132	mRNA_set2	NA	NA	miRDB
GRK3	mRNA_set2	NA	NA	miRDB
SAMD10	mRNA_set2	NA	NA	miRDB
FSTL1	mRNA_set2	NA	NA	miRDB
HOOK1	mRNA_set2	NA	NA	miRDB
CXCL6	mRNA_set2	NA	NA	miRDB
IPO9	mRNA_set2	NA	NA	miRDB
GAS2L1	mRNA_set2	NA	NA	miRDB
KALRN	mRNA_set2	NA	NA	miRDB
SLC16A3	mRNA_set2	NA	NA	miRDB
CHD6	mRNA_set2	NA	NA	miRDB
TTR	mRNA_set2	NA	NA	miRDB
PABPC4L	mRNA_set2	NA	NA	miRDB
SHANK2	mRNA_set2	NA	NA	miRDB
CACNA1C	mRNA_set2	NA	NA	miRDB
CHAC1	mRNA_set2	NA	NA	miRDB
PDZD11	mRNA_set2	NA	NA	miRDB
FXN	mRNA_set2	NA	NA	miRDB
C1QTNF7	mRNA_set2	NA	NA	miRDB
DAB2IP	mRNA_set2	NA	NA	miRDB
TUB	mRNA_set2	NA	NA	miRDB
DCAF10	mRNA_set2	NA	NA	miRDB
TMEM87A	mRNA_set2	NA	NA	miRDB
DIMT1	mRNA_set2	NA	NA	miRDB
FAT1	mRNA_set2	NA	NA	miRDB
POU2F1	mRNA_set2	NA	NA	miRDB

SLC4A1AP	mRNA_set2	NA	NA	miRDB
FASN	mRNA_set2	NA	NA	miRDB
METTL4	mRNA_set2	NA	NA	miRDB
NOX4	mRNA_set2	NA	NA	miRDB
ZNF99	mRNA_set2	NA	NA	miRDB
ZWILCH	mRNA_set2	NA	NA	miRDB
MYCN	mRNA_set2	NA	NA	miRDB
PJA2	mRNA_set2	NA	NA	miRDB
ZNF644	mRNA_set2	NA	NA	miRDB
AGAP1	mRNA_set2	NA	NA	miRDB
WDR11	mRNA_set2	NA	NA	miRDB
CSNK2B	mRNA_set2	NA	NA	miRDB
CCDC181	mRNA_set2	NA	NA	miRDB
DSG3	mRNA_set2	NA	NA	miRDB
CRNKL1	mRNA_set2	NA	NA	miRDB
SRP72	mRNA_set2	NA	NA	miRDB
NUDCD1	mRNA_set2	NA	NA	miRDB
ZNF75D	mRNA_set2	NA	NA	miRDB
RAB22A	mRNA_set2	NA	NA	miRDB
SMARCA4	mRNA_set2	NA	NA	miRDB
PET117	mRNA_set2	NA	NA	miRDB
TMBIM4	mRNA_set2	NA	NA	miRDB
HNRNPD	mRNA_set2	NA	NA	miRDB
PANK2	mRNA_set2	NA	NA	miRDB
TC2N	mRNA_set2	NA	NA	miRDB
MTX3	mRNA_set2	NA	NA	miRDB
MAP7	mRNA_set2	NA	NA	miRDB
SP6	mRNA_set2	NA	NA	miRDB

CSMD1	mRNA_set2	NA	NA	miRDB
CLEC12A	mRNA_set2	NA	NA	miRDB
DMXL2	mRNA_set2	NA	NA	miRDB
NCOA3	mRNA_set2	NA	NA	miRDB
NEUROD6	mRNA_set2	NA	NA	miRDB
TMUB1	mRNA_set2	NA	NA	miRDB
DHTKD1	mRNA_set2	NA	NA	miRDB
BAG2	mRNA_set2	NA	NA	miRDB
SLC4A8	mRNA_set2	NA	NA	miRDB
NSA2	mRNA_set2	NA	NA	miRDB
CCNYL1	mRNA_set2	NA	NA	miRDB
RRP12	mRNA_set2	NA	NA	miRDB
CCR9	mRNA_set2	NA	NA	miRDB
TLNRD1	mRNA_set2	NA	NA	miRDB
ERI3	mRNA_set2	NA	NA	miRDB
ZNF431	mRNA_set2	NA	NA	miRDB
GNA13	mRNA_set2	NA	NA	miRDB
FAM222A	mRNA_set2	NA	NA	miRDB
RGS6	mRNA_set2	NA	NA	miRDB
ZNF510	mRNA_set2	NA	NA	miRDB
PTGER4	mRNA_set2	NA	NA	miRDB
TMEM25	mRNA_set2	NA	NA	miRDB
TMEM41B	mRNA_set2	NA	NA	miRDB
RNF19B	mRNA_set2	NA	NA	miRDB
CACNA1A	mRNA_set2	NA	NA	miRDB
PNRC2	mRNA_set2	NA	NA	miRDB
SYNC	mRNA_set2	NA	NA	miRDB
KAT14	mRNA_set2	NA	NA	miRDB

TMEM132B	mRNA_set2	NA	NA	miRDB
RELCH	mRNA_set2	NA	NA	miRDB
CHMP4B	mRNA_set2	NA	NA	miRDB
ZNF704	mRNA_set2	NA	NA	miRDB
ELMO1	mRNA_set2	NA	NA	miRDB
PHF21B	mRNA_set2	NA	NA	miRDB
ABHD12	mRNA_set2	NA	NA	miRDB
NCLN	mRNA_set2	NA	NA	miRDB
SLC25A46	mRNA_set2	NA	NA	miRDB
PAPSS2	mRNA_set2	NA	NA	miRDB
KLK13	mRNA_set2	NA	NA	miRDB
CDC42	mRNA_set2	NA	NA	miRDB
SMG7	mRNA_set2	NA	NA	miRDB
C15orf62	mRNA_set2	NA	NA	miRDB
TBC1D5	mRNA_set2	NA	NA	miRDB
UBXN10	mRNA_set2	NA	NA	miRDB
NADK	mRNA_set2	NA	NA	miRDB
BCLAF3	mRNA_set2	NA	NA	miRDB
TECPR2	mRNA_set2	NA	NA	miRDB
MTRNR2L3	mRNA_set2	NA	NA	miRDB
ZNF771	mRNA_set2	NA	NA	miRDB
CYP4B1	mRNA_set2	NA	NA	miRDB
H3F3B	mRNA_set2	NA	NA	miRDB
MAN1C1	mRNA_set2	NA	NA	miRDB
AGPAT1	mRNA_set2	NA	NA	miRDB
ISY1-RAB43	mRNA_set2	NA	NA	miRDB
SNTB2	mRNA_set2	NA	NA	miRDB
GH2	mRNA_set2	NA	NA	miRDB

TGIF1	mRNA_set2	NA	NA	miRDB
MYCL	mRNA_set2	NA	NA	miRDB
HMGA2-AS1	mRNA_set2	NA	NA	miRDB
HEYL	mRNA_set2	NA	NA	miRDB
RP9	mRNA_set2	NA	NA	miRDB
TMEM186	mRNA_set2	NA	NA	miRDB
GINS3	mRNA_set2	NA	NA	miRDB
SEC14L5	mRNA_set2	NA	NA	miRDB
BDKRB2	mRNA_set2	NA	NA	miRDB
C17orf80	mRNA_set2	NA	NA	miRDB
DUSP3	mRNA_set2	NA	NA	miRDB
KLK15	mRNA_set2	NA	NA	miRDB
RWDD2A	mRNA_set2	NA	NA	miRDB
COG8	mRNA_set2	NA	NA	miRDB
RAD51B	mRNA_set2	NA	NA	miRDB
PRKD1	mRNA_set2	NA	NA	miRDB
GALM	mRNA_set2	NA	NA	miRDB
IL5RA	mRNA_set2	NA	NA	miRDB
PHF14	mRNA_set2	NA	NA	miRDB
TTC33	mRNA_set2	NA	NA	miRDB
FAM110B	mRNA_set2	NA	NA	miRDB
MEGF9	mRNA_set2	NA	NA	miRDB
PCK1	mRNA_set2	NA	NA	miRDB
MACF1	mRNA_set2	NA	NA	miRDB
CASC4	mRNA_set2	NA	NA	miRDB
CEP76	mRNA_set2	NA	NA	miRDB
SPAG6	mRNA_set2	NA	NA	miRDB
MLF2	mRNA_set2	NA	NA	miRDB

FUCA2	mRNA_set2	NA	NA	miRDB
RIMS2	mRNA_set2	NA	NA	miRDB
ADAMTS1	mRNA_set2	NA	NA	miRDB
R3HDM1	mRNA_set2	NA	NA	miRDB
TMEM263	mRNA_set2	NA	NA	miRDB
GPATCH2	mRNA_set2	NA	NA	miRDB
LRRC7	mRNA_set2	NA	NA	miRDB
FAM78B	mRNA_set2	NA	NA	miRDB
RAP1GDS1	mRNA_set2	NA	NA	miRDB
MEX3D	mRNA_set2	NA	NA	miRDB
ISPD	mRNA_set2	NA	NA	miRDB
RNF126	mRNA_set2	NA	NA	miRDB
CYP2U1	mRNA_set2	NA	NA	miRDB
MICU3	mRNA_set2	NA	NA	miRDB
PRR11	mRNA_set2	NA	NA	miRDB
EFNA2	mRNA_set2	NA	NA	miRDB
SLC16A12	mRNA_set2	NA	NA	miRDB
PVR	mRNA_set2	NA	NA	miRDB
LSM12	mRNA_set2	NA	NA	miRDB
UBE2G1	mRNA_set2	NA	NA	miRDB
NDST3	mRNA_set2	NA	NA	miRDB
MMGT1	mRNA_set2	NA	NA	miRDB
RALYL	mRNA_set2	NA	NA	miRDB
PGR	mRNA_set2	NA	NA	miRDB
GABRP	mRNA_set2	NA	NA	miRDB
FBXO36	mRNA_set2	NA	NA	miRDB
PKNOX2	mRNA_set2	NA	NA	miRDB
FBLN2	mRNA_set2	NA	NA	miRDB

ID2	mRNA_set2	NA	NA	miRDB
ENAH	mRNA_set2	NA	NA	miRDB
KRTAP4-5	mRNA_set2	NA	NA	miRDB
SNRPD1	mRNA_set2	NA	NA	miRDB
ADPRH	mRNA_set2	NA	NA	miRDB
KRBOX4	mRNA_set2	NA	NA	miRDB
RASGRP1	mRNA_set2	NA	NA	miRDB
NOL10	mRNA_set2	NA	NA	miRDB
ZBTB1	mRNA_set2	NA	NA	miRDB
GLG1	mRNA_set2	NA	NA	miRDB
HLA-DQB2	mRNA_set2	NA	NA	miRDB
TNFRSF6B	mRNA_set2	NA	NA	miRDB
FBP1	mRNA_set2	NA	NA	miRDB
CDC42BPA	mRNA_set2	NA	NA	miRDB
H6PD	mRNA_set2	NA	NA	miRDB
C2orf68	mRNA_set2	NA	NA	miRDB
LEF1	mRNA_set2	NA	NA	miRDB
WDFY1	mRNA_set2	NA	NA	miRDB
CDH8	mRNA_set2	NA	NA	miRDB
MYO15A	mRNA_set2	NA	NA	miRDB
KANK1	mRNA_set2	NA	NA	miRDB
ABI2	mRNA_set2	NA	NA	miRDB
GDF11	mRNA_set2	NA	NA	miRDB
PIANP	mRNA_set2	NA	NA	miRDB
MTERF1	mRNA_set2	NA	NA	miRDB
RIOK2	mRNA_set2	NA	NA	miRDB
FOXS1	mRNA_set2	NA	NA	miRDB
NFIC	mRNA_set2	NA	NA	miRDB

ANKFY1	mRNA_set2	NA	NA	miRDB
ZNF410	mRNA_set2	NA	NA	miRDB
PPM1F	mRNA_set2	NA	NA	miRDB
OGFRL1	mRNA_set2	NA	NA	miRDB
KIAA0825	mRNA_set2	NA	NA	miRDB
MMP24	mRNA_set2	NA	NA	miRDB
MINDY1	mRNA_set2	NA	NA	miRDB
CIAO2A	mRNA_set2	NA	NA	miRDB
CALHM5	mRNA_set2	NA	NA	miRDB
UBXN8	mRNA_set2	NA	NA	miRDB
ZNF143	mRNA_set2	NA	NA	miRDB
LARP1	mRNA_set2	NA	NA	miRDB
ATF7IP	mRNA_set2	NA	NA	miRDB
CDC42SE2	mRNA_set2	NA	NA	miRDB
IL21	mRNA_set2	NA	NA	miRDB
COL4A3BP	mRNA_set2	NA	NA	miRDB
BST1	mRNA_set2	NA	NA	miRDB
DIDO1	mRNA_set2	NA	NA	miRDB
PRMT2	mRNA_set2	NA	NA	miRDB
C12orf66	mRNA_set2	NA	NA	miRDB
TRMT12	mRNA_set2	NA	NA	miRDB
TTPAL	mRNA_set2	NA	NA	miRDB
PNRC1	mRNA_set2	NA	NA	miRDB
SYT4	mRNA_set2	NA	NA	miRDB
WDR26	mRNA_set2	NA	NA	miRDB
ARL6IP6	mRNA_set2	NA	NA	miRDB
ARHGAP12	mRNA_set2	NA	NA	miRDB
CCNL1	mRNA_set2	NA	NA	miRDB

EREG	mRNA_set2	NA	NA	miRDB
WFDC8	mRNA_set2	NA	NA	miRDB
HECTD2	mRNA_set2	NA	NA	miRDB
STK38L	mRNA_set2	NA	NA	miRDB
KRT6C	mRNA_set2	NA	NA	miRDB
HOXB8	mRNA_set2	NA	NA	miRDB
CUBN	mRNA_set2	NA	NA	miRDB
RASEF	mRNA_set2	NA	NA	miRDB
ZNF619	mRNA_set2	NA	NA	miRDB
MYSM1	mRNA_set2	NA	NA	miRDB
RNF6	mRNA_set2	NA	NA	miRDB
SLC27A4	mRNA_set2	NA	NA	miRDB
CASC10	mRNA_set2	NA	NA	miRDB
SIN3A	mRNA_set2	NA	NA	miRDB
ADRA1A	mRNA_set2	NA	NA	miRDB
ZNF140	mRNA_set2	NA	NA	miRDB
AGO2	mRNA_set2	NA	NA	miRDB
SLC26A2	mRNA_set2	NA	NA	miRDB
NSG1	mRNA_set2	NA	NA	miRDB
AARD	mRNA_set2	NA	NA	miRDB
CAPRIN2	mRNA_set2	NA	NA	miRDB
DOK6	mRNA_set2	NA	NA	miRDB
ERCC8	mRNA_set2	NA	NA	miRDB
RNF180	mRNA_set2	NA	NA	miRDB
COL27A1	mRNA_set2	NA	NA	miRDB
INIP	mRNA_set2	NA	NA	miRDB
AOX1	mRNA_set2	NA	NA	miRDB
AMOTL1	mRNA_set2	NA	NA	miRDB

HERC3	mRNA_set2	NA	NA	miRDB
BMPR1A	mRNA_set2	NA	NA	miRDB
ARL2BP	mRNA_set2	NA	NA	miRDB
SHPK	mRNA_set2	NA	NA	miRDB
MED28	mRNA_set2	NA	NA	miRDB
ZPLD1	mRNA_set2	NA	NA	miRDB
MYO19	mRNA_set2	NA	NA	miRDB
COL10A1	mRNA_set2	NA	NA	miRDB
MTF1	mRNA_set2	NA	NA	miRDB
YTHDC1	mRNA_set2	NA	NA	miRDB
PDE4B	mRNA_set2	NA	NA	miRDB
MAF1	mRNA_set2	NA	NA	miRDB
RPL36A	mRNA_set2	NA	NA	miRDB
C5orf24	mRNA_set2	NA	NA	miRDB
DCAF5	mRNA_set2	NA	NA	miRDB
CYB5B	mRNA_set2	NA	NA	miRDB
OSTF1	mRNA_set2	NA	NA	miRDB
HNRNPK	mRNA_set2	NA	NA	miRDB
PTMA	mRNA_set2	NA	NA	miRDB
SYT15	mRNA_set2	NA	NA	miRDB
LDAH	mRNA_set2	NA	NA	miRDB
FGD4	mRNA_set2	NA	NA	miRDB
VTI1A	mRNA_set2	NA	NA	miRDB
EID1	mRNA_set2	NA	NA	miRDB
SHROOM3	mRNA_set2	NA	NA	miRDB
FAM83A	mRNA_set2	NA	NA	miRDB
MED1	mRNA_set2	NA	NA	miRDB
CSH2	mRNA_set2	NA	NA	miRDB

SUZ12	mRNA_set2	NA	NA	miRDB
KANK3	mRNA_set2	NA	NA	miRDB
NDEL1	mRNA_set2	NA	NA	miRDB
C9orf170	mRNA_set2	NA	NA	miRDB
ALG1	mRNA_set2	NA	NA	miRDB
ZIK1	mRNA_set2	NA	NA	miRDB
PPM1G	mRNA_set2	NA	NA	miRDB
XAF1	mRNA_set2	NA	NA	miRDB
RNF41	mRNA_set2	NA	NA	miRDB
PRPH2	mRNA_set2	NA	NA	miRDB
GRIP2	mRNA_set2	NA	NA	miRDB
SLTM	mRNA_set2	NA	NA	miRDB
LRP1	mRNA_set2	NA	NA	miRDB
SYVN1	mRNA_set2	NA	NA	miRDB
HNF4G	mRNA_set2	NA	NA	miRDB
PALM2-AKAP2	mRNA_set2	NA	NA	miRDB
ZFP90	mRNA_set2	NA	NA	miRDB
TRPC5OS	mRNA_set2	NA	NA	miRDB
SEMA5A	mRNA_set2	NA	NA	miRDB
SH3PXD2A	mRNA_set2	NA	NA	miRDB
AKAP2	mRNA_set2	NA	NA	miRDB
FAM13A	mRNA_set2	NA	NA	miRDB
GPATCH2L	mRNA_set2	NA	NA	miRDB
CP	mRNA_set2	NA	NA	miRDB
DRAM2	mRNA_set2	NA	NA	miRDB
CASP7	mRNA_set2	NA	NA	miRDB
F5	mRNA_set2	NA	NA	miRDB
NUFIP2	mRNA_set2	NA	NA	miRDB

PLPBP	mRNA_set2	NA	NA	miRDB
TNFSF11	mRNA_set2	NA	NA	miRDB
GABRA6	mRNA_set2	NA	NA	miRDB
MKRN2	mRNA_set2	NA	NA	miRDB
PTER	mRNA_set2	NA	NA	miRDB
STK26	mRNA_set2	NA	NA	miRDB
CAMK4	mRNA_set2	NA	NA	miRDB
YIPF6	mRNA_set2	NA	NA	miRDB
ABRAXAS2	mRNA_set2	NA	NA	miRDB
THSD7A	mRNA_set2	NA	NA	miRDB
RNF13	mRNA_set2	NA	NA	miRDB
PM20D2	mRNA_set2	NA	NA	miRDB
MCTS1	mRNA_set2	NA	NA	miRDB
MAPK8	mRNA_set2	NA	NA	miRDB
SLC9A2	mRNA_set2	NA	NA	miRDB
CEP57L1	mRNA_set2	NA	NA	miRDB
TJP1	mRNA_set2	NA	NA	miRDB
SLC30A8	mRNA_set2	NA	NA	miRDB
IGSF3	mRNA_set2	NA	NA	miRDB
OSTM1	mRNA_set2	NA	NA	miRDB
CNTNAP4	mRNA_set2	NA	NA	miRDB
GLRX	mRNA_set2	NA	NA	miRDB
PGBD5	mRNA_set2	NA	NA	miRDB
ACTR8	mRNA_set2	NA	NA	miRDB
RIT1	mRNA_set2	NA	NA	miRDB
ASIC1	mRNA_set2	NA	NA	miRDB
GORASP1	mRNA_set2	NA	NA	miRDB
POP1	mRNA_set2	NA	NA	miRDB

KCNJ1	mRNA_set2	NA	NA	miRDB
EI24	mRNA_set2	NA	NA	miRDB
NKAPD1	mRNA_set2	NA	NA	miRDB
GCNT2	mRNA_set2	NA	NA	miRDB
FGL2	mRNA_set2	NA	NA	miRDB
PRKCH	mRNA_set2	NA	NA	miRDB
KIAA1549	mRNA_set2	NA	NA	miRDB
ZFX	mRNA_set2	NA	NA	miRDB
DAP3	mRNA_set2	NA	NA	miRDB
ZBED4	mRNA_set2	NA	NA	miRDB
ADGRE1	mRNA_set2	NA	NA	miRDB
PANX1	mRNA_set2	NA	NA	miRDB
BBC3	mRNA_set2	NA	NA	miRDB
MBOAT2	mRNA_set2	NA	NA	miRDB
PSMD1	mRNA_set2	NA	NA	miRDB
C11orf53	mRNA_set2	NA	NA	miRDB
LRPPRC	mRNA_set2	NA	NA	miRDB
S1PR2	mRNA_set2	NA	NA	miRDB
PRRG4	mRNA_set2	NA	NA	miRDB
ERC2	mRNA_set2	NA	NA	miRDB
EMX2	mRNA_set2	NA	NA	miRDB
EMG1	mRNA_set2	NA	NA	miRDB
COLEC12	mRNA_set2	NA	NA	miRDB
MAB21L3	mRNA_set2	NA	NA	miRDB
RBM38	mRNA_set2	NA	NA	miRDB
CDON	mRNA_set2	NA	NA	miRDB
ASAP1	mRNA_set2	NA	NA	miRDB
CCND1	mRNA_set2	NA	NA	miRDB

C19orf44	mRNA_set2	NA	NA	miRDB
PTPRS	mRNA_set2	NA	NA	miRDB
POLR2K	mRNA_set2	NA	NA	miRDB
YES1	mRNA_set2	NA	NA	miRDB
PML	mRNA_set2	NA	NA	miRDB
MED31	mRNA_set2	NA	NA	miRDB
NR4A2	mRNA_set2	NA	NA	miRDB
CBX6	mRNA_set2	NA	NA	miRDB
HNRNPA1	mRNA_set2	NA	NA	miRDB
UBXN2B	mRNA_set2	NA	NA	miRDB
POTEM	mRNA_set2	NA	NA	miRDB
RALGAPA1	mRNA_set2	NA	NA	miRDB
ADGRV1	mRNA_set2	NA	NA	miRDB
FANCI	mRNA_set2	NA	NA	miRDB
NLGN4X	mRNA_set2	NA	NA	miRDB
VWA1	mRNA_set2	NA	NA	miRDB
FBLN7	mRNA_set2	NA	NA	miRDB
TAF9B	mRNA_set2	NA	NA	miRDB
MAPK1IP1L	mRNA_set2	NA	NA	miRDB
GALNTL6	mRNA_set2	NA	NA	miRDB
ZNF527	mRNA_set2	NA	NA	miRDB
TMCC3	mRNA_set2	NA	NA	miRDB
PNMA8A	mRNA_set2	NA	NA	miRDB
FAM111A	mRNA_set2	NA	NA	miRDB
C4orf33	mRNA_set2	NA	NA	miRDB
CBLN4	mRNA_set2	NA	NA	miRDB
CHST11	mRNA_set2	NA	NA	miRDB
SLC7A14	mRNA_set2	NA	NA	miRDB

CDH19	mRNA_set2	NA	NA	miRDB
PLCXD3	mRNA_set2	NA	NA	miRDB
ENAM	mRNA_set2	NA	NA	miRDB
EXOC5	mRNA_set2	NA	NA	miRDB
SERTAD2	mRNA_set2	NA	NA	miRDB
C1orf56	mRNA_set2	NA	NA	miRDB
CDH9	mRNA_set2	NA	NA	miRDB
DOCK8	mRNA_set2	NA	NA	miRDB
PCGF5	mRNA_set2	NA	NA	miRDB
DNAJA2	mRNA_set2	NA	NA	miRDB
KLRC2	mRNA_set2	NA	NA	miRDB
HLTF	mRNA_set2	NA	NA	miRDB
KLRC1	mRNA_set2	NA	NA	miRDB
TCAIM	mRNA_set2	NA	NA	miRDB
NXPH1	mRNA_set2	NA	NA	miRDB
HSBP1L1	mRNA_set2	NA	NA	miRDB
SCAMP1	mRNA_set2	NA	NA	miRDB
SDC2	mRNA_set2	NA	NA	miRDB
GNL3L	mRNA_set2	NA	NA	miRDB
ZC3H12D	mRNA_set2	NA	NA	miRDB
NOLC1	mRNA_set2	NA	NA	miRDB
SLC6A6	mRNA_set2	NA	NA	miRDB
SMAD1	mRNA_set2	NA	NA	miRDB
GIGYF2	mRNA_set2	NA	NA	miRDB
CALM3	mRNA_set2	NA	NA	miRDB
MAP7D3	mRNA_set2	NA	NA	miRDB
ARHGEF9	mRNA_set2	NA	NA	miRDB
ZNF350	mRNA_set2	NA	NA	miRDB

GLP1R	mRNA_set2	NA	NA	miRDB
CIAO1	mRNA_set2	NA	NA	miRDB

Supplementary Table 2. The panel of miRNAs predicted to be regulated by hsa_circRNA_0040462 as reversely assessed from mRNA_set1 and directly via Miranda prediction. Results obtained from reverse assessment from mRNA_set1 were denoted as 'set1', and from Miranda prediction were denoted as 'set2'. The definitions of mRNA_set1, mRNA_set2, miRNA_set1, miRNA_set2 were illustrated in Figure 4A.

miRNA	Data source	Prediction tool
hsa-miR-27a-3p	miRNA_set2	Miranda
hsa-miR-32-3p	miRNA_set2	Miranda
hsa-miR-103a-2-5p	miRNA_set2	Miranda
hsa-miR-148a-3p	miRNA_set2	Miranda
hsa-miR-212-5p	miRNA_set2	Miranda
hsa-miR-1-3p	miRNA_set2	Miranda
hsa-miR-15b-3p	miRNA_set2	Miranda
hsa-miR-27b-3p	miRNA_set2	Miranda
hsa-miR-124-3p	miRNA_set1	starBase
hsa-miR-146a-5p	miRNA_set1	starBase
hsa-miR-942-5p	miRNA_set1	starBase
hsa-miR-502-5p	miRNA_set1	starBase
hsa-miR-128-3p	miRNA_set1	starBase
hsa-miR-1-3p	miRNA_set1	starBase
hsa-miR-181a-5p	miRNA_set1	starBase
hsa-miR-26b-5p	miRNA_set1	starBase
hsa-miR-8057	miRNA_set1	starBase
hsa-miR-24-3p	miRNA_set1	starBase
hsa-miR-4284	miRNA_set1	starBase
hsa-miR-4772-3p	miRNA_set1	starBase

hsa-miR-1304-3p	miRNA_set1	starBase
hsa-miR-6887-3p	miRNA_set1	starBase
hsa-miR-4726-3p	miRNA_set1	starBase
hsa-miR-6764-5p	miRNA_set1	starBase
hsa-miR-1915-3p	miRNA_set1	starBase
hsa-miR-6840-3p	miRNA_set1	starBase
hsa-miR-6890-3p	miRNA_set1	starBase
hsa-miR-6878-3p	miRNA_set1	starBase
hsa-miR-640	miRNA_set1	starBase
hsa-miR-4757-5p	miRNA_set1	starBase
hsa-miR-6744-3p	miRNA_set1	starBase
hsa-miR-6829-3p	miRNA_set1	starBase
hsa-miR-6791-3p	miRNA_set1	starBase
hsa-miR-5009-3p	miRNA_set1	starBase
hsa-miR-212-5p	miRNA_set1	starBase
hsa-miR-3934-5p	miRNA_set1	starBase
hsa-miR-764	miRNA_set1	starBase
hsa-miR-125a-3p	miRNA_set1	starBase
hsa-miR-4796-3p	miRNA_set1	starBase
hsa-miR-93-5p	miRNA_set1	starBase
hsa-miR-526b-3p	miRNA_set1	starBase
hsa-miR-519d-3p	miRNA_set1	starBase
hsa-miR-20b-5p	miRNA_set1	starBase
hsa-miR-20a-5p	miRNA_set1	starBase
hsa-miR-17-5p	miRNA_set1	starBase
hsa-miR-106b-5p	miRNA_set1	starBase
hsa-miR-106a-5p	miRNA_set1	starBase
hsa-miR-548ah-5p	miRNA_set1	starBase

hsa-miR-3609	miRNA_set1	starBase
hsa-miR-378a-5p	miRNA_set1	starBase
hsa-miR-5589-5p	miRNA_set1	starBase
hsa-miR-4731-5p	miRNA_set1	starBase
hsa-miR-6821-3p	miRNA_set1	starBase
hsa-miR-6790-3p	miRNA_set1	starBase
hsa-miR-6506-5p	miRNA_set1	starBase
hsa-miR-619-5p	miRNA_set1	starBase
hsa-miR-3653-5p	miRNA_set1	starBase
hsa-miR-512-3p	miRNA_set1	starBase
hsa-miR-520e	miRNA_set1	starBase
hsa-miR-520d-3p	miRNA_set1	starBase
hsa-miR-520c-3p	miRNA_set1	starBase
hsa-miR-520b	miRNA_set1	starBase
hsa-miR-520a-3p	miRNA_set1	starBase
hsa-miR-373-3p	miRNA_set1	starBase
hsa-miR-372-3p	miRNA_set1	starBase
hsa-miR-302e	miRNA_set1	starBase
hsa-miR-302d-3p	miRNA_set1	starBase
hsa-miR-302c-3p	miRNA_set1	starBase
hsa-miR-302b-3p	miRNA_set1	starBase
hsa-miR-302a-3p	miRNA_set1	starBase
hsa-miR-744-5p	miRNA_set1	starBase
hsa-miR-5698	miRNA_set1	starBase
hsa-miR-6805-3p	miRNA_set1	starBase
hsa-miR-5691	miRNA_set1	starBase
hsa-miR-143-5p	miRNA_set1	starBase
hsa-miR-6879-3p	miRNA_set1	starBase

hsa-miR-4430	miRNA_set1	starBase
hsa-miR-3652	miRNA_set1	starBase
hsa-miR-3622b-5p	miRNA_set1	starBase
hsa-miR-122-5p	miRNA_set1	starBase
hsa-miR-504-3p	miRNA_set1	starBase
hsa-miR-3135b	miRNA_set1	starBase
hsa-miR-3194-3p	miRNA_set1	starBase
hsa-miR-6499-3p	miRNA_set1	starBase
hsa-miR-4772-5p	miRNA_set1	starBase
hsa-miR-383-5p	miRNA_set1	starBase
hsa-miR-215-3p	miRNA_set1	starBase
hsa-miR-6773-3p	miRNA_set1	starBase
hsa-miR-5693	miRNA_set1	starBase
hsa-miR-500b-3p	miRNA_set1	starBase
hsa-miR-1260a	miRNA_set1	starBase
hsa-miR-4314	miRNA_set1	starBase
hsa-miR-4486	miRNA_set1	starBase
hsa-miR-6860	miRNA_set1	starBase
hsa-miR-612	miRNA_set1	starBase
hsa-miR-5189-5p	miRNA_set1	starBase
hsa-miR-3187-5p	miRNA_set1	starBase
hsa-miR-1285-3p	miRNA_set1	starBase
hsa-miR-532-3p	miRNA_set1	starBase
hsa-miR-1224-3p	miRNA_set1	starBase
hsa-miR-6877-3p	miRNA_set1	starBase
hsa-miR-6819-3p	miRNA_set1	starBase
hsa-miR-5189-3p	miRNA_set1	starBase
hsa-miR-6872-3p	miRNA_set1	starBase

hsa-miR-6884-3p	miRNA_set1	starBase
hsa-miR-1972	miRNA_set1	starBase
hsa-miR-622	miRNA_set1	starBase
hsa-miR-15a-3p	miRNA_set1	starBase
hsa-miR-543	miRNA_set1	starBase
hsa-miR-4746-3p	miRNA_set1	starBase
hsa-miR-4792	miRNA_set1	starBase
hsa-miR-6086	miRNA_set1	starBase
hsa-miR-377-5p	miRNA_set1	starBase
hsa-miR-4537	miRNA_set1	starBase
hsa-miR-887-5p	miRNA_set1	starBase
hsa-miR-6780a-5p	miRNA_set1	starBase
hsa-miR-6779-5p	miRNA_set1	starBase
hsa-miR-3689c	miRNA_set1	starBase
hsa-miR-3689b-3p	miRNA_set1	starBase
hsa-miR-3689a-3p	miRNA_set1	starBase
hsa-miR-30b-3p	miRNA_set1	starBase
hsa-miR-1273h-5p	miRNA_set1	starBase
hsa-miR-6788-5p	miRNA_set1	starBase
hsa-miR-30c-2-3p	miRNA_set1	starBase
hsa-miR-30c-1-3p	miRNA_set1	starBase
hsa-miR-7977	miRNA_set1	starBase
hsa-miR-6799-5p	miRNA_set1	starBase
hsa-miR-6883-5p	miRNA_set1	starBase
hsa-miR-6785-5p	miRNA_set1	starBase
hsa-miR-4728-5p	miRNA_set1	starBase
hsa-miR-149-3p	miRNA_set1	starBase
hsa-miR-7106-5p	miRNA_set1	starBase

hsa-miR-383-3p	miRNA_set1	starBase
hsa-miR-3614-5p	miRNA_set1	starBase
hsa-miR-6809-3p	miRNA_set1	starBase
hsa-miR-6833-3p	miRNA_set1	starBase
hsa-miR-4768-5p	miRNA_set1	starBase
hsa-miR-4777-3p	miRNA_set1	starBase
hsa-miR-10a-5p	miRNA_set1	starBase
hsa-let-7d-5p	miRNA_set1	starBase
hsa-miR-599	miRNA_set1	starBase
hsa-miR-3161	miRNA_set1	starBase
hsa-miR-3180-5p	miRNA_set1	starBase
hsa-miR-525-5p	miRNA_set1	starBase
hsa-miR-520a-5p	miRNA_set1	starBase
hsa-miR-1264	miRNA_set1	starBase
hsa-miR-3159	miRNA_set1	starBase
hsa-miR-3929	miRNA_set1	starBase
hsa-miR-4329	miRNA_set1	starBase
hsa-miR-4419b	miRNA_set1	starBase
hsa-miR-4433a-3p	miRNA_set1	starBase
hsa-miR-4459	miRNA_set1	starBase
hsa-miR-4478	miRNA_set1	starBase
hsa-miR-4524a-3p	miRNA_set1	starBase
hsa-miR-4524b-3p	miRNA_set1	starBase
hsa-miR-4695-5p	miRNA_set1	starBase
hsa-miR-4768-3p	miRNA_set1	starBase
hsa-miR-485-5p	miRNA_set1	starBase
hsa-miR-506-5p	miRNA_set1	starBase
hsa-miR-665	miRNA_set1	starBase

hsa-miR-6884-5p	miRNA_set1	starBase
hsa-miR-6894-5p	miRNA_set1	starBase
hsa-miR-892c-5p	miRNA_set1	starBase
hsa-miR-550a-3p	miRNA_set1	starBase
hsa-miR-200c-5p	miRNA_set1	starBase
hsa-miR-4680-5p	miRNA_set1	starBase
hsa-miR-1255b-2-3p	miRNA_set1	starBase
hsa-miR-618	miRNA_set1	starBase
hsa-miR-4803	miRNA_set1	starBase
hsa-miR-4309	miRNA_set1	starBase
hsa-miR-183-5p	miRNA_set1	starBase
hsa-miR-3198	miRNA_set1	starBase
hsa-miR-4294	miRNA_set1	starBase
hsa-miR-1289	miRNA_set1	starBase
hsa-miR-4663	miRNA_set1	starBase
hsa-miR-610	miRNA_set1	starBase
hsa-miR-4267	miRNA_set1	starBase
hsa-miR-7702	miRNA_set1	starBase
hsa-miR-6514-5p	miRNA_set1	starBase
hsa-miR-874-3p	miRNA_set1	starBase
hsa-miR-7158-3p	miRNA_set1	starBase
hsa-miR-151a-3p	miRNA_set1	starBase
hsa-miR-6799-3p	miRNA_set1	starBase
hsa-miR-146b-3p	miRNA_set1	starBase
hsa-miR-7976	miRNA_set1	starBase
hsa-miR-3173-5p	miRNA_set1	starBase
hsa-miR-4539	miRNA_set1	starBase
hsa-miR-18a-3p	miRNA_set1	starBase

hsa-miR-4463	miRNA_set1	starBase
hsa-miR-4499	miRNA_set1	starBase
hsa-miR-4724-5p	miRNA_set1	starBase
hsa-let-7b-3p	miRNA_set1	starBase
hsa-miR-1179	miRNA_set1	starBase
hsa-miR-1290	miRNA_set1	starBase
hsa-miR-142-3p	miRNA_set1	starBase
hsa-miR-182-5p	miRNA_set1	starBase
hsa-miR-23a-3p	miRNA_set1	starBase
hsa-miR-29b-3p	miRNA_set1	starBase
hsa-miR-29c-3p	miRNA_set1	starBase
hsa-miR-3179	miRNA_set1	starBase
hsa-miR-3619-5p	miRNA_set1	starBase
hsa-miR-522-5p	miRNA_set1	starBase
hsa-miR-7-5p	miRNA_set1	starBase
hsa-miR-933	miRNA_set1	starBase
hsa-miR-23b-3p	miRNA_set1	starBase
hsa-miR-16-5p	miRNA_set1	starBase
hsa-miR-766-3p	miRNA_set1	starBase
hsa-miR-1343-3p	miRNA_set1	starBase
hsa-miR-129-2-3p	miRNA_set1	starBase
hsa-miR-210-3p	miRNA_set1	starBase
hsa-miR-212-3p	miRNA_set1	starBase
hsa-let-7a-5p	miRNA_set1	starBase
hsa-let-7b-5p	miRNA_set1	starBase
hsa-let-7c-5p	miRNA_set1	starBase
hsa-let-7e-5p	miRNA_set1	starBase
hsa-let-7f-5p	miRNA_set1	starBase

hsa-let-7g-5p	miRNA_set1	starBase
hsa-let-7i-5p	miRNA_set1	starBase
hsa-miR-1243	miRNA_set1	starBase
hsa-miR-126-5p	miRNA_set1	starBase
hsa-miR-130a-3p	miRNA_set1	starBase
hsa-miR-130b-3p	miRNA_set1	starBase
hsa-miR-142-5p	miRNA_set1	starBase
hsa-miR-15a-5p	miRNA_set1	starBase
hsa-miR-15b-5p	miRNA_set1	starBase
hsa-miR-2114-5p	miRNA_set1	starBase
hsa-miR-218-5p	miRNA_set1	starBase
hsa-miR-22-5p	miRNA_set1	starBase
hsa-miR-301a-3p	miRNA_set1	starBase
hsa-miR-301b-3p	miRNA_set1	starBase
hsa-miR-330-3p	miRNA_set1	starBase
hsa-miR-34a-5p	miRNA_set1	starBase
hsa-miR-361-5p	miRNA_set1	starBase
hsa-miR-378a-3p	miRNA_set1	starBase
hsa-miR-424-5p	miRNA_set1	starBase
hsa-miR-4461	miRNA_set1	starBase
hsa-miR-561-5p	miRNA_set1	starBase
hsa-miR-576-3p	miRNA_set1	starBase
hsa-miR-590-3p	miRNA_set1	starBase
hsa-miR-769-5p	miRNA_set1	starBase
hsa-miR-98-5p	miRNA_set1	starBase
hsa-miR-103a-3p	miRNA_set1	starBase
hsa-miR-107	miRNA_set1	starBase
hsa-miR-195-5p	miRNA_set1	starBase

hsa-miR-27a-3p	miRNA_set1	starBase
hsa-miR-192-5p	miRNA_set1	starBase
hsa-miR-147a	miRNA_set1	starBase
hsa-miR-224-5p	miRNA_set1	starBase
hsa-miR-429	miRNA_set1	starBase
hsa-miR-191-5p	miRNA_set1	starBase
hsa-miR-31-5p	miRNA_set1	starBase
hsa-miR-22-3p	miRNA_set1	starBase
hsa-miR-29a-3p	miRNA_set1	starBase
hsa-miR-30a-3p	miRNA_set1	starBase
hsa-miR-30b-5p	miRNA_set1	starBase
hsa-miR-30c-5p	miRNA_set1	starBase
hsa-miR-30d-5p	miRNA_set1	starBase
hsa-miR-30e-3p	miRNA_set1	starBase
hsa-miR-30e-5p	miRNA_set1	starBase
hsa-miR-3617-5p	miRNA_set1	starBase
hsa-miR-603	miRNA_set1	starBase
hsa-miR-641	miRNA_set1	starBase
hsa-miR-376c-3p	miRNA_set1	starBase
hsa-miR-494-3p	miRNA_set1	starBase
hsa-miR-101-3p	miRNA_set1	starBase
hsa-miR-10b-5p	miRNA_set1	starBase
hsa-miR-155-5p	miRNA_set1	starBase
hsa-miR-26a-5p	miRNA_set1	starBase
hsa-miR-941	miRNA_set1	starBase
hsa-miR-138-5p	miRNA_set1	starBase
hsa-miR-200b-3p	miRNA_set1	starBase
hsa-miR-203a-3p	miRNA_set1	starBase

hsa-miR-206	miRNA_set1	starBase
hsa-miR-21-3p	miRNA_set1	starBase
hsa-miR-221-3p	miRNA_set1	starBase
hsa-miR-27a-5p	miRNA_set1	starBase
hsa-miR-27b-3p	miRNA_set1	starBase
hsa-miR-374a-5p	miRNA_set1	starBase
hsa-miR-133a-3p	miRNA_set1	starBase
hsa-miR-9-5p	miRNA_set1	starBase
hsa-miR-2467-5p	miRNA_set1	starBase
hsa-miR-548ax	miRNA_set1	starBase
hsa-miR-378b	miRNA_set1	starBase
hsa-miR-378c	miRNA_set1	starBase

Supplementary Table 3. KEGG pathways and GO terms enriched by the union of mRNA_set1 and mRNA_set2, and by mRNA_set2. Results obtained using the union of mRNA_set1 and mRNA_set2 were denoted as 'set1+2', and from mRNA_set2 were denoted as 'set2'. The definitions of mRNA_set1, mRNA_set2, miRNA_set1, miRNA_set2 were illustrated in Figure 4A.

ID	Description	Data source	Prediction tool	Gene Ratio _set2	GeneRatio_set1+2	BgRatio _set2	BgRatio_ set1+2	pvalue _set2	pvalue _set1+2	p.adjust _set2	p.adjust _set1+2
hsa04151	PI3K-Akt signaling pathway	set1+2, set2	KEGG	102/1412	102/1419	354/8086	354/8096	4.68E-08	5.69E-08	3.78E-06	4.59E-06
hsa04010	MAPK signaling pathway	set1+2, set2	KEGG	89/1412	89/1419	294/8086	294/8096	2.87E-08	3.44E-08	3.09E-06	3.70E-06
hsa04144	Endocytosis	set1+2, set2	KEGG	73/1412	73/1419	252/8086	252/8096	3.21E-06	3.70E-06	8.65E-05	9.64E-05
hsa05205	Proteoglycans in cancer	set1+2, set2	KEGG	71/1412	71/1419	205/8086	205/8096	1.59E-09	1.88E-09	5.15E-07	6.09E-07
hsa04014	Ras signaling pathway	set1+2, set2	KEGG	70/1412	70/1419	232/8086	232/8096	1.02E-06	1.17E-06	3.28E-05	3.78E-05
hsa04810	Regulation of actin cytoskeleton	set1+2, set2	KEGG	68/1412	69/1419	218/8086	218/8096	3.75E-07	1.92E-07	2.02E-05	1.24E-05
hsa04510	Focal adhesion	set1+2, set2	KEGG	64/1412	64/1419	201/8086	201/8096	3.66E-07	4.20E-07	2.02E-05	2.26E-05
hsa04068	FoxO signaling pathway	set1+2, set2	KEGG	51/1412	51/1419	131/8086	131/8096	3.81E-09	4.35E-09	6.16E-07	7.03E-07
hsa04390	Hippo signaling	set2	KEGG	50/1412	NA	157/8086	NA	6.84E-06	NA	0.000134087	NA

	pathway										
hsa04910	Insulin signaling pathway	set2	KEGG	47/141 2	NA	137/8086	NA	1.28E-06	NA	3.75E-05	NA
hsa04550	Signaling pathways regulating pluripotency of stem cells	set2	KEGG	46/141 2	NA	143/8086	NA	1.18E-05	NA	0.0002008 95	NA
hsa04722	Neurotrophin signaling pathway	set2	KEGG	43/141 2	NA	119/8086	NA	7.35E-07	NA	2.64E-05	NA
hsa04919	Thyroid hormone signaling pathway	set2	KEGG	40/141 2	NA	121/8086	NA	2.12E-05	NA	0.0003395 82	NA
hsa04931	Insulin resistance	set1+2, set2	KEGG	38/141 2	38/1419	108/8086	108/8096	6.67E-06	7.30E-06	0.0001340 87	0.0001455 45
hsa05032	Morphine addiction	set1+2, set2	KEGG	36/141 2	36/1419	91/8086	91/8096	4.72E-07	5.18E-07	2.18E-05	2.39E-05
hsa04012	ErbB signaling pathway	set1+2, set2	KEGG	34/141 2	34/1419	85/8086	85/8096	7.21E-07	7.89E-07	2.64E-05	2.93E-05
hsa04211	Longevity regulating pathway	set2	KEGG	33/141 2	NA	89/8086	NA	7.47E-06	NA	0.0001340 87	NA
hsa04727	GABAergic synapse	set2	KEGG	33/141 2	NA	89/8086	NA	7.47E-06	NA	0.0001340 87	NA

hsa01521	EGFR tyrosine kinase inhibitor resistance	set1+2, set2	KEGG	31/141 2	31/1419	79/8086	79/8096	3.58E-06	3.88E-06	8.90E-05	9.64E-05
hsa05211	Renal cell carcinoma	set1+2, set2	KEGG	28/141 2	28/1419	69/8086	69/8096	4.90E-06	5.28E-06	0.0001129 85	0.0001217 19
GO:0050 804	modulation of chemical synaptic transmission	set1+2, set2	GO	145/30 90	145/310 6	454/18866	454/18866	9.48E-17	1.49E-16	3.69E-13	5.80E-13
GO:0099 177	regulation of trans-synaptic signaling	set1+2, set2	GO	145/30 90	145/310 6	455/18866	455/18866	1.17E-16	1.84E-16	3.69E-13	5.80E-13
GO:0007 409	axonogenesis	set1+2, set2	GO	145/30 90	145/310 6	482/18866	482/18866	2.45E-14	3.74E-14	5.13E-11	7.86E-11
GO:0031 346	positive regulation of cell projection organization	set1+2, set2	GO	124/30 90	124/310 6	394/18866	394/18866	5.46E-14	7.99E-14	8.59E-11	1.26E-10
GO:0022 604	regulation of cell morphogenesi s	set1+2, set2	GO	147/30 90	147/310 6	499/18866	499/18866	1.05E-13	1.60E-13	1.32E-10	2.01E-10
GO:0030 900	forebrain development	set1+2, set2	GO	122/30 90	122/310 6	391/18866	391/18866	1.72E-13	2.50E-13	1.81E-10	2.63E-10
GO:0016	dendrite	set1+2,	GO	86/309	86/3106	247/18866	247/18866	9.87E-13	1.32E-12	8.88E-10	1.19E-09

358	development	set2		0							
GO:0050<br>808	synapse<br>organization	set1+2,<br>set2	GO	129/30<br>90	129/310<br>6	433/18866	433/18866	1.44E-12	2.10E-12	1.14E-09	1.65E-09
GO:0045<br>666	positive<br>regulation of<br>neuron<br>differentiatio<br>n	set1+2,<br>set2	GO	116/30<br>90	116/310<br>6	380/18866	380/18866	3.42E-12	4.83E-12	2.39E-09	3.38E-09
GO:0050<br>769	positive<br>regulation of<br>neurogenesis	set1+2,<br>set2	GO	139/30<br>90	139/310<br>6	485/18866	485/18866	4.68E-12	6.88E-12	2.95E-09	4.33E-09
GO:0060<br>537	muscle tissue<br>development	set1+2,<br>set2	GO	122/30<br>90	122/310<br>6	409/18866	409/18866	5.36E-12	7.63E-12	3.07E-09	4.37E-09
GO:0010<br>976	positive<br>regulation of<br>neuron<br>projection<br>development	set1+2,<br>set2	GO	94/309<br>0	94/3106	290/18866	290/18866	9.82E-12	1.32E-11	4.77E-09	6.58E-09
GO:0007<br>265	Ras protein<br>signal<br>transduction	set1+2,<br>set2	GO	107/30<br>90	107/310<br>6	346/18866	346/18866	9.84E-12	1.36E-11	4.77E-09	6.58E-09
GO:0021<br>537	telencephalon<br>development	set1+2,<br>set2	GO	85/309<br>0	85/3106	259/18866	259/18866	4.67E-11	6.13E-11	2.10E-08	2.75E-08
GO:0010<br>769	regulation of<br>cell<br>morphogenesi<br>s involved in<br>differentiatio	set1+2,<br>set2	GO	96/309<br>0	96/3106	310/18866	310/18866	1.05E-10	1.40E-10	4.39E-08	5.86E-08

	n										
GO:0098978	glutamatergic synapse	set1+2, set2	GO	128/3196	128/3213	361/19559	361/19559	4.19E-19	6.58E-19	3.11E-16	4.89E-16
GO:0031252	cell leading edge	set1+2, set2	GO	140/3196	140/3213	421/19559	421/19559	5.85E-18	9.35E-18	2.17E-15	3.47E-15
GO:0099572	postsynaptic specialization	set1+2, set2	GO	119/3196	120/3213	361/19559	361/19559	3.80E-15	2.17E-15	9.16E-13	4.02E-13
GO:0014069	postsynaptic density	set1+2, set2	GO	113/3196	114/3213	337/19559	337/19559	4.93E-15	2.70E-15	9.16E-13	4.02E-13
GO:0098984	neuron to neuron synapse	set1+2, set2	GO	120/3196	121/3213	368/19559	368/19559	7.09E-15	4.11E-15	9.35E-13	4.45E-13
GO:0032279	asymmetric synapse	set1+2, set2	GO	114/3196	115/3213	343/19559	343/19559	7.55E-15	4.19E-15	9.35E-13	4.45E-13
GO:0097060	synaptic membrane	set1+2, set2	GO	124/3196	126/3213	387/19559	387/19559	1.09E-14	2.57E-15	1.15E-12	4.02E-13
GO:0045211	postsynaptic membrane	set1+2, set2	GO	93/3196	95/3213	280/19559	280/19559	2.38E-12	4.61E-13	2.21E-10	4.28E-11
GO:0030427	site of polarized growth	set1+2, set2	GO	68/3196	68/3213	191/19559	191/19559	6.79E-11	8.63E-11	5.61E-09	7.13E-09
GO:0150034	distal axon	set1+2, set2	GO	96/3196	96/3213	309/19559	309/19559	7.62E-11	1.03E-10	5.66E-09	7.59E-09
GO:0004674	protein serine/threonine kinase activity	set1+2, set2	GO	126/3159	126/3177	435/18352	435/18352	5.89E-10	8.50E-10	6.78E-07	9.29E-07
GO:0140	DNA-binding	set1+2,	GO	104/31	105/317	347/18352	347/18352	2.50E-09	1.61E-09	1.44E-06	9.29E-07

297	transcription factor binding	set2		59	7						
GO:0061629	RNA polymerase II-specific DNA-binding transcription factor binding	set1+2, set2	GO	81/3159	82/3177	267/18352	267/18352	7.94E-08	4.71E-08	3.05E-05	1.81E-05
GO:0060589	nucleoside-triphosphatase regulator activity	set1+2, set2	GO	99/3159	100/3177	348/18352	348/18352	1.06E-07	7.12E-08	3.06E-05	2.05E-05
GO:0001227	DNA-binding transcription repressor activity, RNA polymerase II-specific	set1+2, set2	GO	95/3159	95/3177	335/18352	335/18352	2.24E-07	2.93E-07	3.99E-05	4.89E-05
GO:0001217	DNA-binding transcription repressor activity	set1+2, set2	GO	95/3159	95/3177	336/18352	336/18352	2.60E-07	3.40E-07	3.99E-05	4.89E-05
GO:0004879	nuclear receptor activity	set1+2, set2	GO	25/3159	25/3177	52/18352	52/18352	2.78E-07	3.11E-07	3.99E-05	4.89E-05
GO:0098531	ligand-activated transcription	set1+2, set2	GO	25/3159	25/3177	52/18352	52/18352	2.78E-07	3.11E-07	3.99E-05	4.89E-05

	factor activity										
GO:0003712	transcription coregulator activity	set1+2, set2	GO	129/3159	129/3177	498/18352	498/18352	5.04E-07	6.91E-07	6.02E-05	8.06E-05
GO:0031267	small GTPase binding	set1+2, set2	GO	114/3159	114/3177	428/18352	428/18352	5.23E-07	7.01E-07	6.02E-05	8.06E-05
GO:0017016	Ras GTPase binding	set1+2, set2	GO	110/3159	110/3177	415/18352	415/18352	1.06E-06	1.41E-06	0.000111227	0.000134822
GO:0030695	GTPase regulator activity	set1+2, set2	GO	86/3159	87/3177	307/18352	307/18352	1.44E-06	9.41E-07	0.00013768	9.85E-05
GO:0005096	GTPase activator activity	set1+2, set2	GO	78/3159	78/3177	275/18352	275/18352	2.60E-06	3.24E-06	0.000230405	0.00028726
GO:0035257	nuclear hormone receptor binding	set1+2, set2	GO	47/3159	47/3177	144/18352	144/18352	4.63E-06	5.43E-06	0.000380778	0.000416359
GO:0042578	phosphoric ester hydrolase activity	set1+2, set2	GO	97/3159	98/3177	369/18352	369/18352	6.58E-06	4.73E-06	0.0004982	0.000388742
